# Prescription and switching patterns of direct oral anticoagulants in patients with atrial fibrillation

**DOI:** 10.1016/j.rpth.2024.102544

**Published:** 2024-08-09

**Authors:** Tim A.C. de Vries, Roisin Bavalia, Gordon Chu, Helen Xiong, Kayleigh M. van de Wiel, Hanne van Ballegooijen, Menno V. Huisman, Martin E.W. Hemels, Saskia Middeldorp, Joris R. de Groot

**Affiliations:** 1 Heart Center, Department of Clinical and Experimental Cardiology and Cardiothoracic Surgery, Amsterdam University Medical Centers location University of Amsterdam, Amsterdam, the Netherlands; 2 Heart Failure and Arrhythmias, Amsterdam Cardiovascular Sciences, Amsterdam, the Netherlands; 3 Heart Center, Department of Cardiology, Amsterdam University Medical Centers location Vrije Universiteit Amsterdam, Amsterdam, the Netherlands; 4 Department of Cardiology, Rijnstate Hospital, Arnhem, the Netherlands; 5 Department of Vascular Medicine, Amsterdam University Medical Centers location University of Amsterdam, Amsterdam, the Netherlands; 6 Department of Cardiology, Amphia Hospital, Breda, the Netherlands; 7 Department of Thrombosis and Hemostasis, Leiden University Medical Center, Leiden, the Netherlands; 8 IQVIA Nederland, Amsterdam, the Netherlands; 9 Department of Cardiology, Radboudumc, Nijmegen, the Netherlands; 10 Department of Internal Medicine, Radboudumc, Nijmegen, the Netherlands

**Keywords:** anticoagulants, atrial fibrillation, big data, drug substitution, thromboembolism

## Abstract

**Background:**

The patterns of direct oral anticoagulant (DOAC) selection and switching to a different oral anticoagulant (OAC) in patients with atrial fibrillation (AF) are unknown.

**Objectives:**

To describe temporal patterns in first DOAC prescriptions, estimate the incidence, and identify predictors of switching to a different OAC within 1 year in OAC-naive AF patients.

**Methods:**

In this retrospective cohort study, using a near-nationwide prescription registry (IQVIA, the Netherlands), we determined the number of patients per month initiated on each DOAC and identified predictors of switching within 1 year with robust Poisson regression.

**Results:**

We included 94,874 patients. From November 2015 to November 2019, the monthly use of apixaban (*n* = 366 to *n* = 1066, +191%), rivaroxaban (*n* = 379 to *n* = 868, +129%), and edoxaban (*n* = 2 to *n* = 305, +15,150%) increased, whereas that of dabigatran decreased (*n* = 317 to *n* = 179, −44%). In the 66,090 patients with ≥1 year of available calendar time, 7% switched to a different OAC within 1 year. Strong predictors of switching to a different DOAC were using dabigatran (adjusted risk ratio [aRR], 3.33; 95% CI, 3.02-3.66) or edoxaban (aRR, 1.56; 95% CI, 1.34-1.82) rather than apixaban and using a standard DOAC dose (aRR, 2.54; 95% CI, 2.23-2.88). Strong predictors of switching to a vitamin K antagonist were using rivaroxaban (aRR, 1.36; 95% CI, 1.19-1.54 vs apixaban) and using a standard DOAC dose (aRR, 1.49; 95% CI, 1.26-1.77).

**Conclusion:**

In the Netherlands, factor Xa inhibitors are increasingly being selected for OAC-naive AF patients. Seven percent of patients switch to a different OAC within 1 year, and the initial DOAC type and dose are strong predictors of switching.

## Essentials

• Prescription and switching patterns of oral anticoagulants for atrial fibrillation are unknown.
• We performed a retrospective cohort study using a near-nationwide pharmacy database.
• The use of the factor Xa inhibitors is increasing steadily.
• Direct oral anticoagulant type and dose predict switching to another oral anticoagulant.

## Graphical abstract

**Figure:**
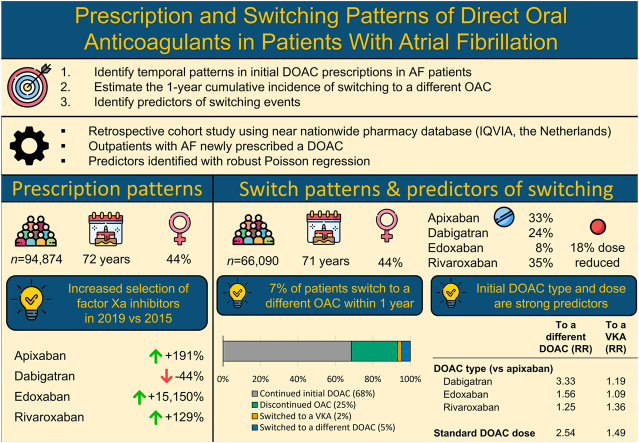

## 1 Introduction

Since their introduction, direct oral anticoagulants (DOACs) have replaced vitamin K antagonists (VKAs) as the preferred oral anticoagulant (OAC) for most patients with atrial fibrillation (AF) [1,2]. This preference is mainly due to 2 reasons. First, DOACs are at least as effective as VKAs in most patients, while causing fewer bleeding complications. Second, DOACs are more convenient because they can be given in fixed doses [[1], [2], [3], [4]]. Based on these considerations, authors of guidelines recommend using DOACs over VKAs in nearly all patients with AF. However, because their relative efficacy is uncertain due to the lack of direct comparisons, there is limited guidance on selecting a specific DOAC (ie, apixaban, dabigatran, edoxaban, or edoxaban) [[3], [4], [5]].

When selecting a DOAC, clinicians must consider the patient’s preference for once vs twice-daily dosing, the size of the pill, need for digestion with food, the dominant mechanism of clearance (renal vs hepatic), the rates of stroke or bleeding associated with their use, the availability of a reversal agent, and other factors [1,3]. Despite these considerations, some patients may be unsatisfied with the selected DOAC. For instance, because of treatment failure (a thromboembolic event despite therapy or a major or recurrent bleeding event while on anticoagulant treatment) or side effects [1]. In such circumstances, clinicians and their patients must decide whether to continue the current DOAC (at the same or different dose) or to switch to a different OAC. Clinicians may also recommend switching to a different OAC for other reasons, such as need for treatment with interacting drugs, or worsening of the renal or hepatic function [3].

Using a retrospective cohort study design, we sought to (i) describe the changes over time in the selection of initial DOAC prescriptions for OAC-naive AF patients in the Netherlands, (ii) estimate the incidence, and (iii) identify predictors of switching to a different OAC within 1 year in these patients.

## 2 Methods

### 2.1 Study design

In this retrospective cohort study, we used data from IQVIA’s Real-World Data Longitudinal Prescription (LRx) database. IQVIA is a provider of advanced analytics, technology solutions, and clinical research services to the life sciences industry. We adhered to the Strengthening the Reporting of Observational studies in Epidemiology recommendations (Supplementary Table S1) [6].

### 2.2 Database characteristics and protection of privacy

The LRx is a database on routinely collected prescriptions from approximately 1500 pharmacies. Included pharmacies are either retail, outpatient polyclinic, or those associated with a general practitioner’s office, and together cover nearly 65% of all outpatient prescriptions dispensed within the Netherlands.

Each pharmacy collects information on personal identifiers and dispensed prescriptions (eg, name, dose, number of units supplied, and date of the prescription dispensation) of individual patients. The data are pseudonymized twice and then sent to IQVIA encrypted by a trusted third party. The use of a unique patient identifier was first implemented on October 1, 2014, and allows for longitudinal follow-up of each patient even when they collected prescriptions at different pharmacies.

### 2.3 Consent

The Medical Ethics Assessment Committee Amsterdam University Medical Center (MEC AMC 018) waived the requirement to obtain informed consent from study participants. We performed this study in compliance with the Declaration of Helsinki.

### 2.4 Health care insurance and coverage

In the Netherlands, all citizens are required to have health insurance and pay an annual premium [7]. In addition, they must meet an annual deductible for most medical expenses, including the dispensation of DOACs, before their insurance covers further costs [7]. Individuals with lower incomes are eligible for financial assistance from the government that may cover both the premium and deductible [7].

### 2.5 Study period, participants, and sample size

The study period was November 1, 2014 (ie, 12 months prior the first included patient) to November 1, 2021 (ie, latest moment of follow-up). To determine if a patient was new to OAC therapy (defined as no use of any OAC in the 12 months prior to the first dispensed DOAC prescription), we only included patients from November 1, 2015. The last included patients collected their first DOAC prescription on October 31, 2019.

We defined 2 sample populations, one to describe the changes in the selection of the initial DOAC prescription over time (ie, the cross-sectional analysis cohort) and another to determine the incidence and identify predictors of switching events (ie, the longitudinal analysis cohort). The final sample size of both cohorts was determined solely by the availability of eligible patients.

Patients who collected a DOAC during the study period were eligible for inclusion. For the cross-sectional analysis cohort, we excluded the patients who collected (i) any OAC in the 12 months prior to the first DOAC prescription fill; (ii) their prescription at pharmacies that inconsistently provided data to IQVIA during the study period; (iii) a DOAC dosing regimen not approved for use in AF in Europe (Supplementary Table S2) [2]; and (iv) more than 1 type of OAC on the day of the initial DOAC prescription fill. We also excluded (v) nonadults (ie, age <18 years) and (vi) those for whom the initial DOAC prescription was for a different indication than AF. To estimate the indication of the DOAC prescription, we developed a decision tree model based on the absolute dose, dosing frequency, and treatment durations of each DOAC as well as pretreatment with low-molecular-weight heparin (Supplementary Figure S1) [2,3,8].

For the longitudinal analysis cohort, we excluded additional patients to ensure sufficient follow-up time was available to determine switching to a different OAC or discontinuation of OAC treatment, and to only select those at risk of switching. We therefore further excluded patients who collected their first DOAC prescription on or after November 1, 2018, and those who only picked up a DOAC prescription once. We also excluded the patients who collected more than 1 type of OAC on the day of the first switching event.

### 2.6 Data collection and variable definitions

We defined baseline as the date of the first DOAC prescription fill. We extracted the baseline patient demographics (ie, age and sex), and use of medication of interest (eg, generic and brand name, dosage, treatment frequency, prescription date, and number of pills dispensed) from the eligible records. We operationalized sex as female or male [9]. The treatment duration of DOAC prescriptions was determined based on the treatment frequency and number of pills supplied.

In accordance with the designs of the randomized trials comparing the DOACs with warfarin, we classified the dosing regimens as either standard or reduced for apixaban, edoxaban, and rivaroxaban, and as higher or lower dose for dabigatran. However, to allow for direct comparisons across the 4 DOACs, we considered the higher and standard dose, and the lower and reduced dose to be analogous (Supplementary Table S2) [3].

Definitions of comedication classes of interest used in the 12 months before or on the same day as the initial DOAC prescription fill were based on the European Pharmaceutical Market Research Association Anatomical Classification Guidelines 2018 or were individual molecules (Supplementary Table S3). We then used these medication classes to estimate the presence of preexisting cardiovascular and mental comorbid conditions (Supplementary Table S4), as well as to calculate a modified Congestive heart failure, Hypertension, Age ≥75 years, Diabetes mellitus, Stroke, Vascular disease, Age 65-74 years, Sex category (female) (CHA_2_DS_2_-VASc) score (Supplementary Table S5). The CHA_2_DS_2_-VASc score predicts the annual risk of stroke for individual patients with AF and is primarly used to guide the initial decision to start treatment with an OAC [3,5].

**Figure 1:**
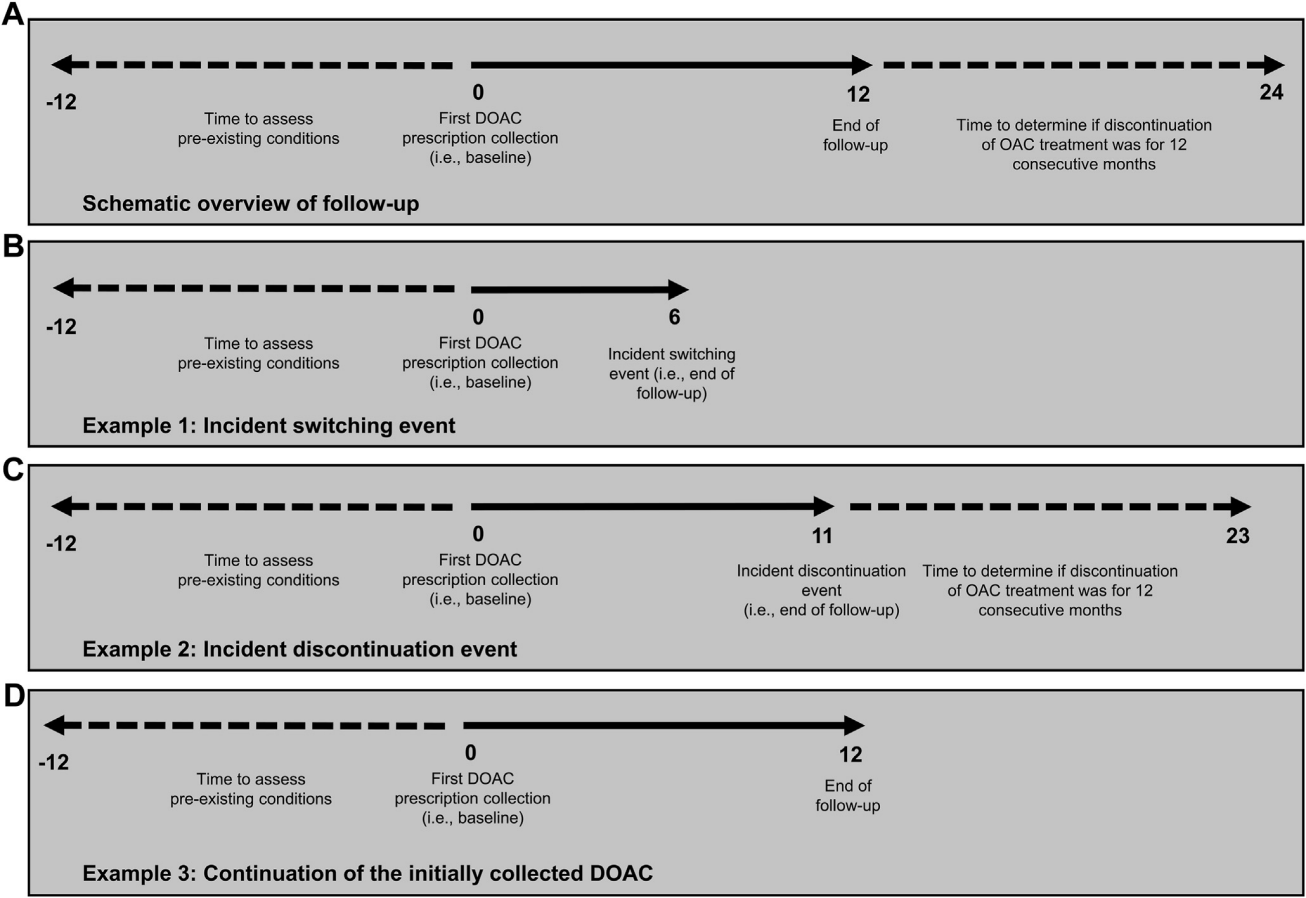
Schematic overview of follow-up time. (A) All patients were followed for 12 months or until the occurrence of an incident event of interest. We identified preexisting comorbid conditions using the prescriptions collected in the 12 months prior to the first dispensed direct oral anticoagulant (DOAC) prescription (Supplementary Tables S3–S5). (B) Example of an incident switching event at 6 months. (C) Example of an incident discontinuation event at 11 months. We used the 12 months hereafter to determine if such an event was for 12 consecutive months. (D) Example of continuation of the initially collected DOAC (ie, absence of an event). DOAC, direct oral anticoagulant; OAC, oral anticoagulant.

We defined incident switching events as the first collection of (i) a different DOAC or (ii) a VKA after the index DOAC prescription. Discontinuation of OAC treatment was defined as the absence of a refill of any OAC prescription for 12 consecutive months, which includes discontinuation of any OAC but ongoing collection of other prescriptions and discontinuation of all prescription collections. All patients in the longitudinal analysis cohort were followed until a first switching or discontinuation event occurred, or until 1 year of calendar time had passed without the occurrence an event of interest (Figure 1).

### 2.7 Statistical analyses

To summarize the baseline patient characteristics of patients in the 2 analysis cohorts, we used summary statistics appropriate for the expected distribution of the data, both irrespective and stratified by the initially dispensed DOAC type.

To describe the patterns in the selection of initial DOAC prescriptions over time, we determined the total number of patients who collected a DOAC in each month of the study period. We did this both irrespective of and stratified by the initial DOAC type. We also described these patterns by age (ie, ≥75, ≥65 to <75, and <65 years) and sex categories.

To quantify the occurrence of switching to a different OAC, we calculated the 1-year cumulative incidence of switching to a different DOAC or to a VKA, and estimated their 95% confidence intervals (CIs) [10]. We assessed the consistency of our findings by also estimating the 6-month cumulative incidences. We also determined the 1-year cumulative incidence stratified by age (ie, ≥75, ≥65 to <75, and <65 years) and sex categories.

To identify potential predictors of switching to a different OAC within 1 year, we performed univariable and multivariable robust Poisson regression analyses [11,12]. We created 2 models if age (the only continuous covariable) had a nonlinear relationship with the outcome to facilitate the interpretation of the models, one with age modeled with a restricted cubic spline function [13] and another with age categorized in accordance with the knot locations of the optimal fit.

We selected potential predictors based solely on available literature or clinical rationale and then applied the full model approach [[14], [15], [16], [17]]. To avoid high multicollinearity among some of the predictors [18], we created at least 2 full-fit models per outcome of interest, one with all preselected variables included except our modified CHA_2_DS_2_-VASc score and another with this variable included but omitting the underlying covariables (Supplementary Table S5).

We performed a post hoc defined subgroup analysis identifying potential predictors of switching in each subgroup of initially collected DOAC type.

All analyses were performed in R version 4.2.2 on a Windows device [19]. A more detailed description of our statistical analyses is provided in Supplementary Table S6.

## 3 Results

### 3.1 Patient selection

**Figure 2:**
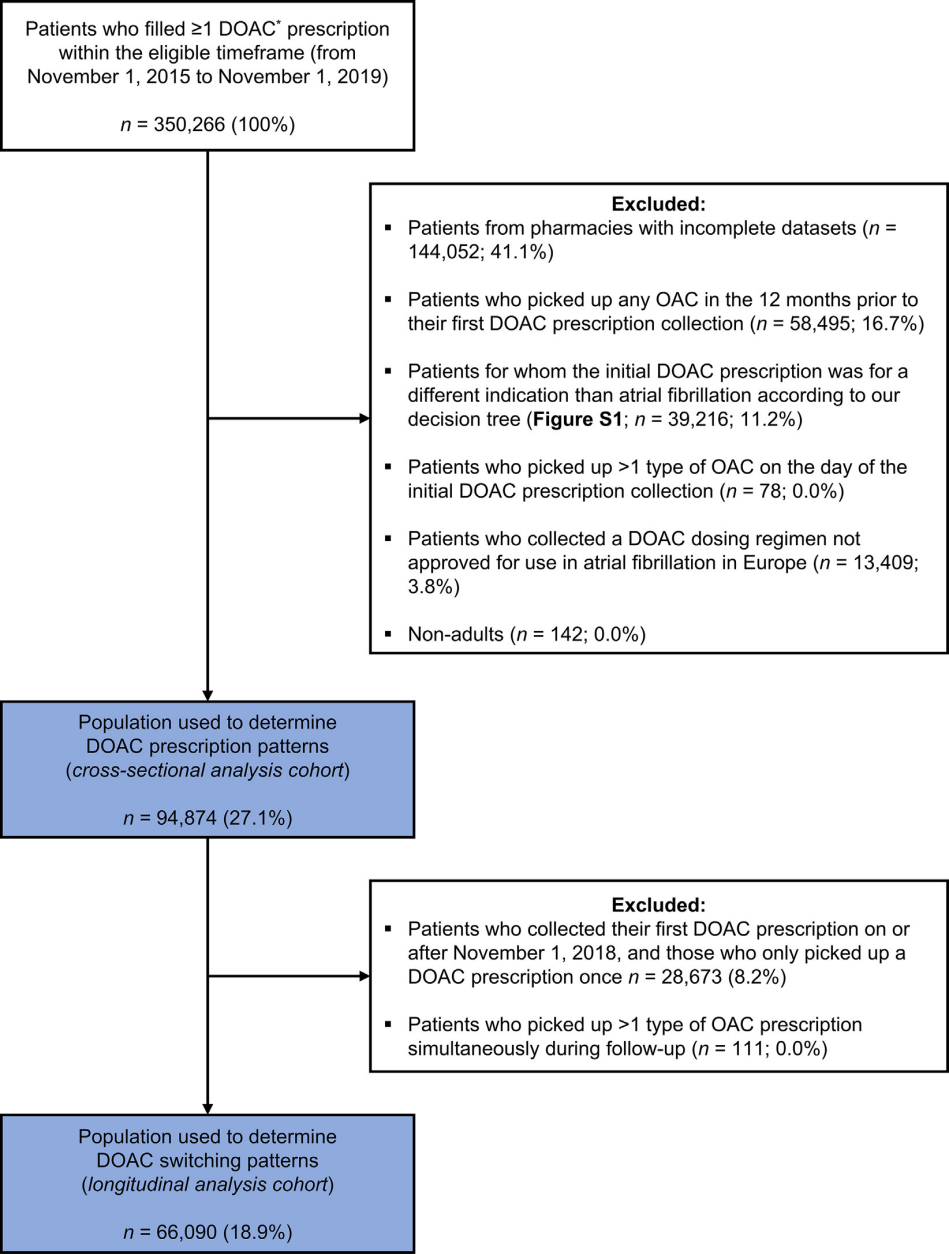
Patient flow. DOAC, direct oral anticoagulant; OAC, oral anticoagulant. ∗Patients treated with rivaroxaban 2.5 mg twice daily were already excluded.

An overview of the patient flow is presented in Figure 2. We identified 350,266 patients who filled at least one DOAC prescription within the eligible timeframe (ie, from November 1, 2015, to November 1, 2019). After exclusion of ineligible patients, 94,874 (27%) were included in the cross-sectional analysis cohort and 66,090 (19%) in the longitudinal analysis cohort.

### 3.2 Baseline participant characteristics

The baseline characteristics of patients in the cross-sectional analysis cohort are summarized in the Table. Of the 94,874 patients, 33,788 (36%) were treated with apixaban, 19,326 (20%) with dabigatran, 8596 (9%) with edoxaban, and 33,164 (35%) with rivaroxaban. Female patients represented a little less than half (*n* = 41,848, 44%) of all patients, and the median age was 72 (IQR, 64-79) years.

**Table:** Baseline characteristics of patients in the cross-sectional analysis cohort.

Characteristic	Total (*n* = 94,874)	Stratified by initially collected DOAC type			
		Apixaban (*n* = 33,788)	Dabigatran (*n* = 19,326)	Edoxaban (*n* = 8596)	Rivaroxaban (*n* = 33,164)
Age (y)	72 (64-79)	72 (64-80)	71 (64-78)	71 (63-79)	71 (63-79)
≥75	37,102 (39.1%)	14,140 (41.8%)	6961 (36.0%)	3257 (37.9%)	12,744 (38.4%)
65-74	31,909 (33.6%)	10,882 (32.2%)	7091 (36.7%)	2897 (33.7%)	11,039 (33.3%)
≤64	25,863 (27.3%)	8766 (25.9%)	5274 (27.3%)	2442 (28.4%)	9381 (28.3%)
Female sex	41,484 (43.7%)	15,171 (44.9%)	8070 (41.8%)	3647 (42.4%)	14,596 (44.0%)
Reduced DOAC dosing regimen	15,644 (16.5%)	3309 (9.8%)	5752 (29.8%)	1554 (18.1%)	5029 (15.2%)
Clinical pattern of atrial fibrillation					
Permanent	54,071 (57.0%)	19,290 (57.1%)	11,461 (59.3%)	4992 (58.1%)	18,328 (55.3%)
Persistent or paroxysmal	12,765 (13.5%)	4438 (13.1%)	2862 (14.8%)	1059 (12.3%)	4406 (13.3%)
Uncertain type	28,038 (29.6%)	10,060 (29.8%)	5003 (25.9%)	2545 (29.6%)	10,430 (31.4%)
Atherosclerotic disease	36,581 (38.6%)	13,414 (39.7%)	7288 (37.7%)	3328 (38.7%)	12,551 (37.8%)
Hypertension or congestive heart failure	52,008 (54.8%)	19,065 (56.4%)	10,426 (53.9%)	4671 (54.3%)	17,846 (53.8%)
Diabetes mellitus	14,509 (15.3%)	5320 (15.7%)	2745 (14.2%)	1310 (15.2%)	5134 (15.5%)
Modified CHA_2_DS_2_VASc score	3 (2-4)	3 (2-5)	3 (2-4)	3 (2-4)	3 (2-4)
High risk (≥2 in males; ≥3 in females)	65,680 (69.2%)	23,966 (70.9%)	13,208 (68.3%)	5845 (68.0%)	22,661 (68.3%)
Medium risk (1 in males; 2 in females)	16,473 (17.4%)	5451 (16.1%)	3582 (18.5%)	1588 (18.5%)	5852 (17.6%)
Low risk (0 in males; 1 in females)	12,721 (13.4%)	4371 (12.9%)	2536 (13.1%)	1163 (13.5%)	4651 (14.0%)
Parkinson’s disease	1599 (1.7%)	582 (1.7%)	277 (1.4%)	137 (1.6%)	603 (1.8%)
Mental disorders necessitating treatment with psycholeptics	18,364 (19.4%)	6605 (19.5%)	3462 (17.9%)	1679 (19.5%)	6618 (20.0%)
Mental disorders necessitating treatment with psychoanaleptics	9887 (10.4%)	3588 (10.6%)	1851 (9.6%)	860 (10.0%)	3588 (10.8%)

In this table, the baseline characteristics of the patients in the cross-sectional analysis cohort are summarized. Presented values are number (%) of patients or median (IQR).

CHA_2_DS_2_-VASc, Congestive heart failure, Hypertension, Age ≥75 years, Diabetes mellitus, Stroke, Vascular disease, Age 65-74 years, Sex category (female); DOAC, direct oral anticoagulant.

The lower dose was prescribed most often to those initiated on dabigatran (*n* = 5752, 30%), and the reduced dose most often to those on edoxaban (*n* = 1554, 18%) and rivaroxaban (*n* = 5029, 15%), and least (*n* = 3309, 10%) to those who collected apixaban as their first DOAC. Other covariables were similar across the 4 DOAC type subgroups.

The distributions of the comedication groups dispensed in the 12 months prior to the initial DOAC prescription fill in the cross-sectional analysis cohort were similar across the 4 DOAC types (Supplementary Table S7). The baseline characteristics of the patients in the longitudinal analysis cohort were near-identical to those of the patients included in the cross-sectional analysis cohort (Supplementary Tables S8 and S9).

### 3.3 Change in DOAC prescriptions over time

**Figure 3:**
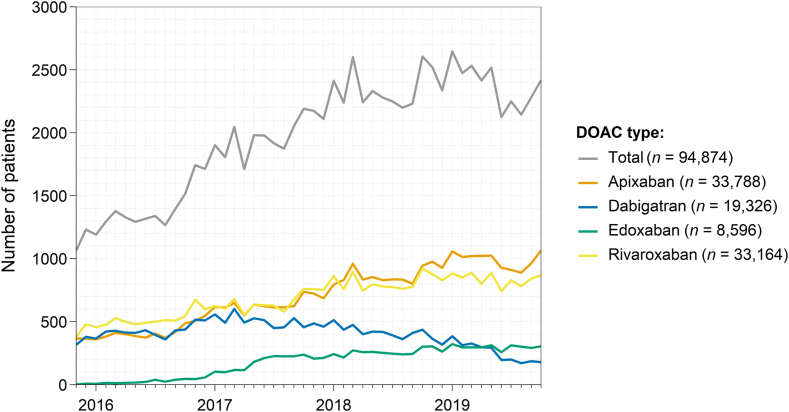
Changes in the collection of direct oral anticoagulants (DOACs) over time. This figure illustrates the number of oral anticoagulant–naive patients per month in the cross-sectional analysis cohort (*n* = 94,874) who collected their first DOAC prescription (from November 1st, 2015 to November 1st, 2019). DOAC, direct oral anticoagulant.

The changes over time in the prescription patterns of DOACs are illustrated in Figure 3. The number of patients newly treated with a DOAC per month increased gradually from 1064 in November 2015 to 2418 in October 2019.

During this period, the number of patients per month initiated on apixaban (*n* = 366 to *n* = 1066, +191%), rivaroxaban (*n* = 379 to *n* = 868, +129%), and edoxaban (*n* = 2 to *n* = 305, +15,150%) increased, whereas that of dabigatran decreased (*n* = 317 to *n* = 179, −44%). These patterns were similar across age and sex categories (Supplementary Figure S2).

### 3.4 Incidence of switching to a different OAC

**Figure 4:**
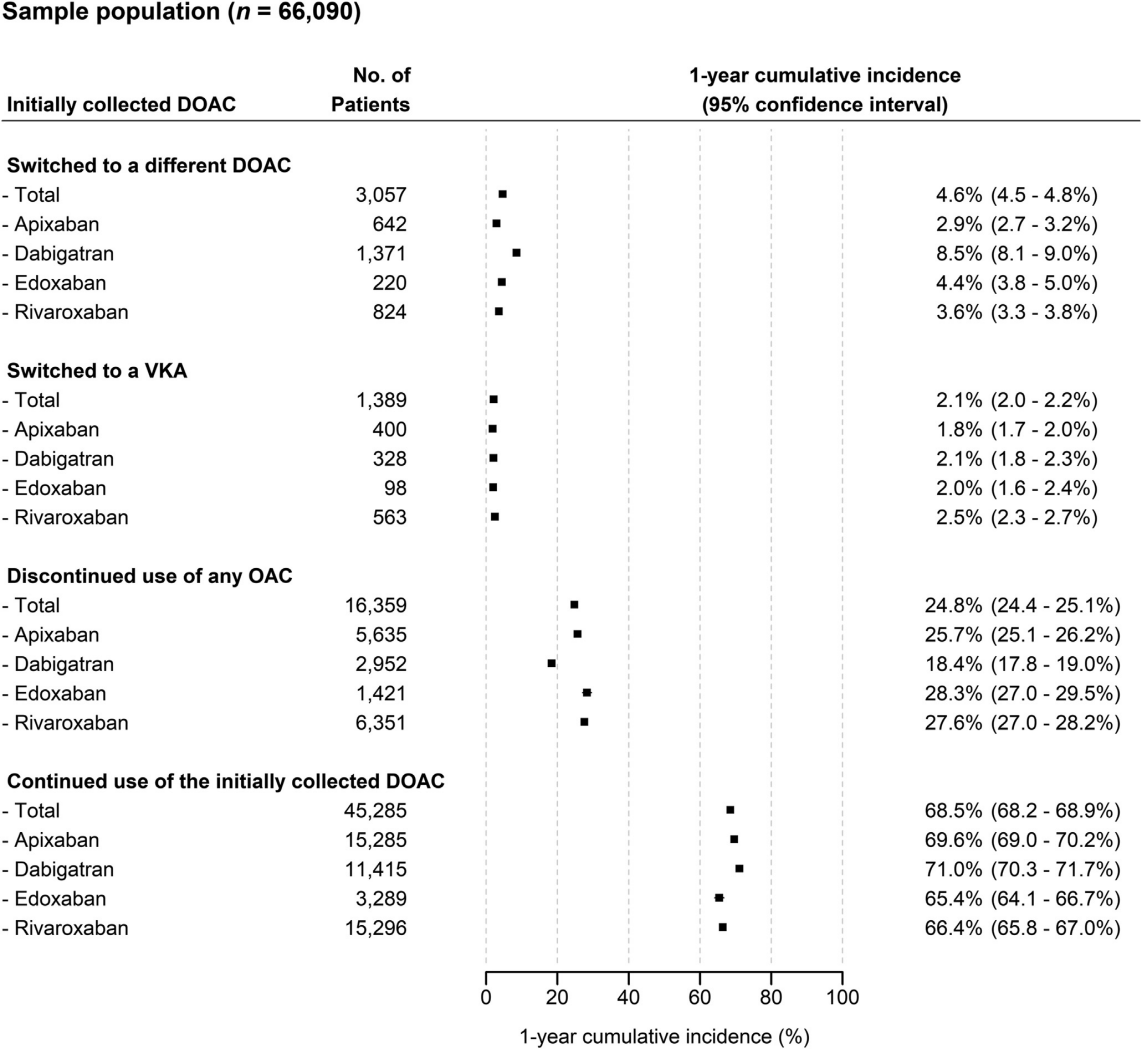
One-year cumulative incidence of switching to a different oral anticoagulant (OAC). This figure illustrates the 1-year cumulative incidences of switching to a different OAC in the longitudinal analysis cohort (*n* = 66,090). DOAC, direct oral anticoagulant; OAC, oral anticoagulant; VKA, vitamin K antagonist.

The cumulative incidences of switching to a different OAC and stratified by initial DOAC type are presented in Figure 4. During the first year of therapy, most patients continued use of their initial DOAC (68.5%; 95% CI, 68.2%-68.9%; *n* = 45,285) or discontinued use of any OAC (24.8%; 95% CI, 24.4%-25.1%; *n* = 16,359).

The 1-year cumulative incidence of switching to an alternative OAC was 6.7% (95% CI, 6.5%-6.9%; *n* = 4446), and most of these patients switched to a different DOAC (4.6%; 95% CI, 4.5%-4.8%; *n* = 3057). The incidence of switching to a different DOAC was highest for patients initiated on dabigatran (8.5%; 95% CI, 8.1%-9.0%; *n* = 1371) and lowest for those on apixaban (2.9%; 95% CI, 2.7%-3.2%; *n* = 642). The 1-year incidence of switching to a VKA was 2.1% (95% CI, 2.0%-2.2%; *n* = 1389). The incidences of such events were similar across the 4 DOAC type subgroups (ranging from 1.8% to 2.5%).

The cumulative 6-month incidence of switching to a different OAC was 4.6% (95% CI, 4.5%-4.8%; *n* = 3057). In the first half year, most of those who switched were initiated on a different DOAC (3.1%; 95% CI, 3.0%-3.3%; *n* = 2075), with patients for whom the first DOAC was dabigatran having the highest incidence of such events (5.8%; 95% CI, 5.4%-6.1%; *n* = 928) and lowest for those on apixaban (2.0%; 95% CI, 1.8%-2.1%; *n* = 427; Supplementary Figure S3). The 1-year cumulative incidence varied across the age and sex categories, but the same trends in differences across the 4 DOAC types were observed (Supplementary Figure S4). The 1-year cumulative incidences of (i) switching to individual DOACs, (ii) the incidences of discontinuation of collection of any OAC but ongoing collection of other drugs, and (iii) discontinuation of collection of any prescription, are presented in Supplementary Figure S5.

### 3.5 Predictors of switching to a different OAC

**Figure 5:**
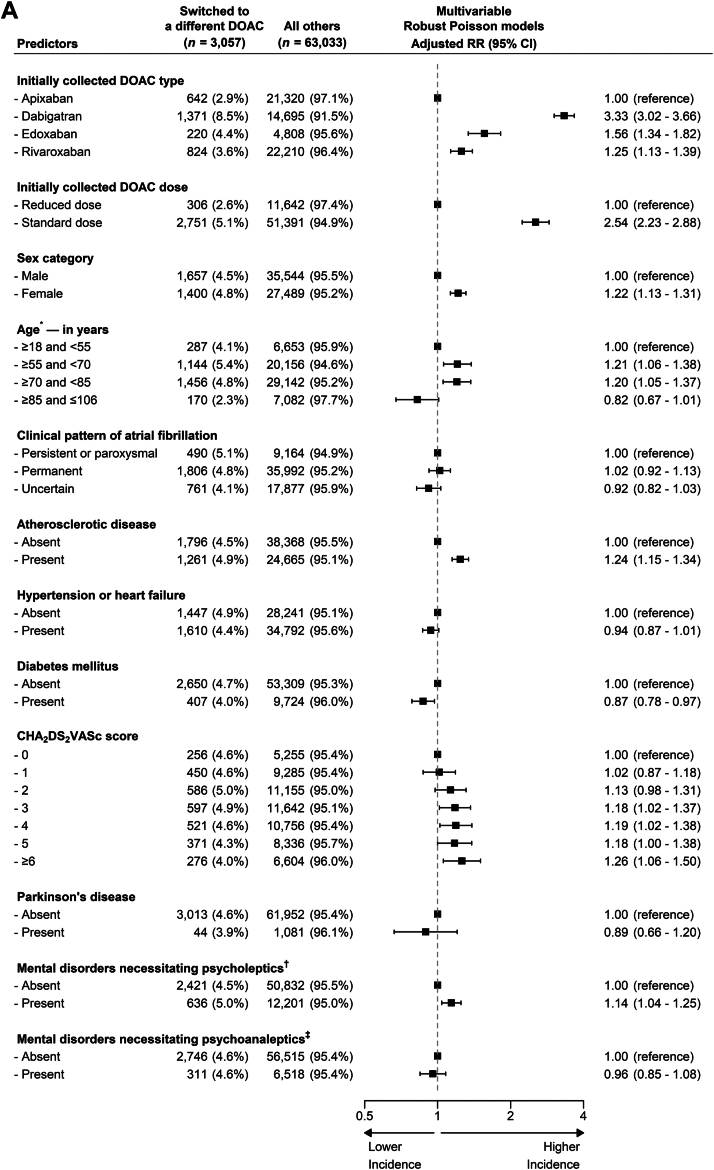

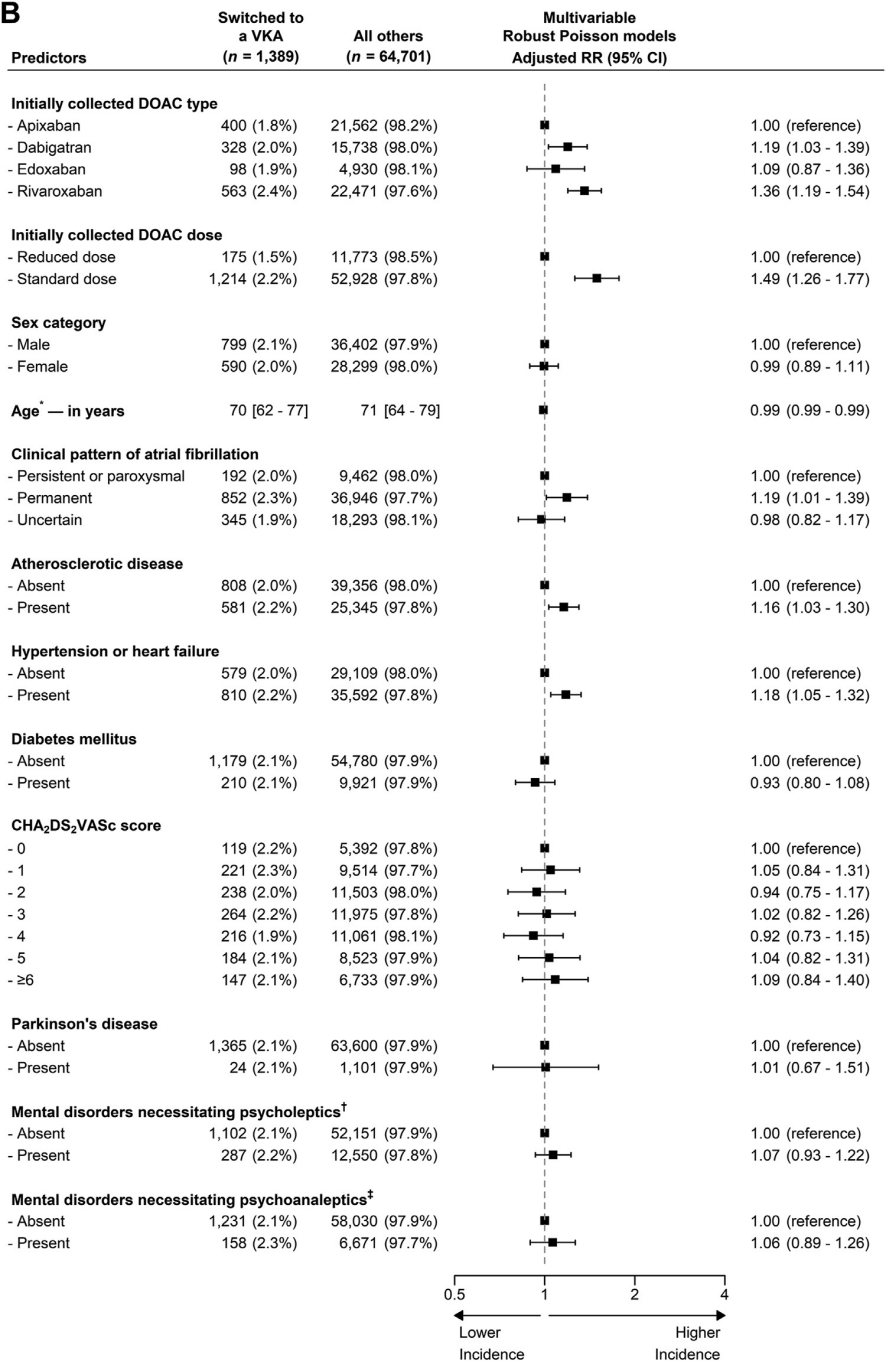
Adjusted predictors of switching within 1 year. (A) To a different direct oral anticoagulant (DOAC). This figure illustrates the adjusted associations between baseline characteristics and switching to a different DOAC within 1 year in the longitudinal analysis cohort (*n* = 66,090). Baseline characteristics are in number of patients (percentage of stratum total), and the associations are in risk ratios (RRs). CHA_2_DS_2_-VASc, Congestive heart failure, Hypertension, Age ≥75 years, Diabetes mellitus, Stroke, Vascular disease, Age 65-74 years, Sex category (female). ∗Categories were determined by the knot locations (ie, 10th, 50th, and 90th percentile) of the spline function with the optimal fit [13]. ^†^Examples are sleeping, anxiety, and psychotic disorders. ^‡^Examples are depressive and bipolar disorders. (B) To a vitamin K antagonist (VKA). This figure illustrates the adjusted associations between baseline characteristics and switching to a VKA within 1 year in the longitudinal analysis cohort (*n* = 66,090). Baseline characteristics are in number of patients (percentage of stratum total), and the associations are in RRs. ∗The relationship between age and incident switching was best fitted by a linear function in both the univariable and multivariable model. ^†^Examples are sleeping, anxiety, and psychotic disorders. ^‡^Examples are depressive and bipolar disorders.

The adjusted associations of switching to a different DOAC as well as those of to VKA are presented in Figure 5. The strongest predictors of switching to a different DOAC were use of dabigatran (adjusted risk ratio [aRR], 3.33; 95% CI, 3.02-3.66), edoxaban (aRR, 1.56; 95% CI, 1.34-1.82), or rivaroxaban (aRR, 1.25; 95% CI, 1.13-1.39) rather than apixaban, and use of a standard DOAC dose (aRR, 2.54; 95% CI, 2.23-2.88). Other predictors of such events were female sex (aRR, 1.22; 95% CI, 1.13-1.31), age between ≥55 and <70 years (aRR, 1.21; 95% CI, 1.06-1.38) and between ≥70 and <85 years (aRR, 1.20; 95% CI, 1.05-1.37), a history of atherosclerotic disease (aRR, 1.24; 95% CI, 1.15-1.34), a modified CHA_2_DS_2_-VASc score of ≥3 (aRRs for these scores, ≥1.18; range of 95% CIs, 1.00-1.50), and a history of mental disorder necessitating treatment with psycholeptics treatment (aRR, 1.14; 95% CI, 1.04-1.25). The unadjusted and adjusted associations were similar (Supplementary Figure S6), and fitting age as a ratio variable with a restricted cubic spline function had limited effect on the associations for the other predictors (Supplementary Figures S7 and S8).

Our post hoc defined analysis on potential predictors in the 4 subgroups of initially collected DOAC type did not confirm all predictors of switching to a different DOAC as identified in our main analysis (Supplementary Figure S9A). Female sex was a strong predictor for patients who collected dabigatran as their first DOAC (aRR, 1.40; 95% CI, 1.27-1.55), but not for those on the other DOACs. Also contrasting our main analysis was the lower incidence of switching with higher CHA_2_DS_2_-VASc scores for patients who collected apixaban as the initial DOAC (aRRs, ≤0.80; range of 95% CIs, 0.47-1.08) and an increasingly higher incidences for higher scores for those on dabigatran (aRRs, ≥1.19; range of 95% CIs, 0.95-2.54).

The strongest predictors of switching to a VKA were use of rivaroxaban (aRR, 1.36; 95% CI, 1.19-1.54), and use of a standard DOAC dose (aRR, 1.49; 95% CI, 1.26-1.77). Other predictors of these switching events were use of dabigatran (aRR, 1.19; 95% CI, 1.03-1.39), and a history of permanent AF (aRR, 1.19; 95% CI, 1.01-1.39), atherosclerotic disease (aRR, 1.16; 95% CI, 1.03-1.30) or the composite of hypertension or heart failure (aRR, 1.18; 95% CI, 1.05-1.32). The unadjusted and adjusted associations were near-identical (Supplementary Figure S6).

Our post hoc defined subgroup analysis did not suggest major differences across the 4 DOAC type subgroups for potential predictors of switching to a VKA (Supplementary Figure S9B).

## 4 Discussion

The 3 main findings of this retrospective cohort study are as follows: (i) the use of the factor (F)Xa inhibitors (ie, apixaban, rivaroxaban, and edoxaban) is steadily increasing in the Netherlands for patients with AF new to OAC treatment, whereas that of dabigatran is decreasing (Figure 3); (ii) the 1-year cumulative incidence switching to a different OAC is about 7% in these patients, and 2- to 3-fold higher for those initiated on dabigatran than those on the FⅩa inhibitors (Figure 4); and (iii) the initiated DOAC type and dose are strong predictors of switching to a different OAC (Figure 5). We discuss these and other findings in more detail below.

### 4.1 The use of the FⅩa inhibitors for AF is increasing

In each month from November 2015 to November 2019, both apixaban and rivaroxaban were most often prescribed to OAC-naive patients with AF (Figure 3). These observations are consistent with studies from other regions [20,21]. For instance, in the United States, the proportion of all OAC prescription claims in the field of cardiology that were for apixaban increased from approximately 20% (*n* = 1,000,000) in 2015 to 45% (*n* = 3,100,000), whereas that of rivaroxaban was about 25% in both 2015 and 2018 (*n* = 1,200,000 to *n* = 1,700,000). As in our study, prescription claims for dabigatran decreased during the same period (from 8%, *n* = 400,000 in 2015 to 4%, *n* = 300,000 in 2018) [20].

Clinicians may prefer apixaban because low-quality evidence indicates it may have a more favorable safety profile compared with the other DOACs [22]. Moreover, rivaroxaban is also often prescribed possibly due to its convenience of once daily dosing [1], and as supported by our findings, it being on the market for longer than edoxaban [2,8]. A novel finding in our study is the growing preference for edoxaban as the primary DOAC for patients with AF (Figure 3). Edoxaban’s increased use may be attributable to its convenience of once daily dosing and the advantage over rivaroxaban that it does not have to be taken together with food to optimize its bioavailability [1,2,8].

### 4.2 Seven in 100 patients switch to a different OAC within 1 year

In the Netherlands, about 7% of patients newly treated with a DOAC switch to a different OAC, and of those who switch, more than two-thirds are prescribed a different DOAC (Figure 4). These observations do not confirm those of another historical cohort study on similar patients treated in the United States. In this American cohort of 38,947 health-insured patients, between 8% and 15% of switched to a different OAC within 1 year, and of those who switched, most were subsequently prescribed a VKA (54% of 2939 patients) [15].

The higher percentage of switching to a VKA in the United States could be explained by differences in insurance coverage between countries [23]. Another explanation for the difference is more recent data being included in our study (inclusion of patients from 2015 onward compared with from 2009 onward) [15]. Since their introduction, the DOACs have increasingly penetrated the market and many clinicians have become more familiar with using them [1]. It is possible that, with increasing exposure, clinicians now have greater confidence in continuing the initially selected DOAC if they anticipate limited benefits from switching to another OAC, and also prioritize a different DOAC over a VKA whenever switching is contemplated.

### 4.3 The DOAC type and dosing regimen are strong predictors of switching

Adjusted for other predictors, the type of DOAC and its dosing regimen best predict switching to a different DOAC, and the same variables are also strong predictors of switching to a VKA. Other notable predictors of switching to a different DOAC are female sex, age between 55 and 85 years, a history of atherosclerotic disease, and a high CHA_2_DS_2_-VASc score (Figure 5).

These findings support previous research but also show other factors have potential predictive utility [16]. For instance, a historical cohort study on 41,864 American patients found that being initiated on apixaban (odds ratio, 0.25; 95% CI, 0.18-0.34) or rivaroxaban (odds ratio, 0.41; 95% CI, 0.38-0.44) rather than dabigatran, were inversely associated with switching to an alternative OAC. Age and sex categories were also significant predictors with the same direction and a similar magnitude of effect as observed in our cohort of patients treated in the Netherlands [16].

Our post hoc defined subgroup analysis suggests the predictive utility of the factors identified for switching to a different DOAC may not be consistent for all DOAC types. Though the validity of these analyses is affected by reduced statistical power and high probability of chance findings due to the large number of comparisons, these findings can inform future studies on the identification of predictors of switching.

Taken together, the predictors identified in our study, both the ones confirming the existing literature and those newly established, may inform future efforts to better identify the patients most likely to switch to a different OAC.

### 4.4 Limitations

Our study has 2 major limitations. The first limitation is the potential for selection bias, as we excluded many patients who picked up their initial DOAC prescription at pharmacies that did not consistently provide data to IQVIA during the study period. We considered alternative approaches, such as including only patients with complete follow-up or including all patients regardless of completeness of follow-up. However, we believe excluding data at the pharmacy level was the approach least prone to bias. Since we did not collect information on the excluded pharmacies or their patients, we cannot assess whether our sample is biased.

The second limitation is that our dataset has limited information on patient demographics (eg, race and socioeconomic factors are unavailable) and lacks direct information on medical conditions. Instead, we estimated the indication for each initial DOAC prescription (Supplementary Figure S1). Since this is an approximation, we may have erroneously included patients prescribed a DOAC for venous thromboembolism and excluded some with AF. We believe the risk of such misclassifications is highest for apixaban and lowest for rivaroxaban.

This is because we identified patients collecting a first prescription for apixaban 2.5 mg twice daily for 10 to 14 or 28 to 38 days. In the absence of subsequent dispensations, we estimated these patients to have been treated for the primary prevention of venous thromboembolism after knee or hip surgery [2,8]. Some of these patients may have had AF but were excluded from our analyses. Consequently, we might have underestimated the selection of apixaban as the initial DOAC for AF-related stroke prevention.

Furthermore, rivaroxaban has the longest initial treatment period for venous thromboembolism among all DOACs (with either a loading dose or use of a parental anticoagulant), lasting 21 days compared with 5 or 7 days [2,8]. After this period, the DOAC dosing strategy is the same as that used for preventing AF-related stroke. Since our dataset includes only outpatients, we may have misclassified some patients with venous thromboembolism who were discharged after the initial period. Due to this difference in treatment strategies, the likelihood of overestimating the use of apixaban, dabigatran, or edoxaban as the initial DOAC for AF is higher compared with rivaroxaban. The impact of these misclassifications on the incidence and predictors of switching is unpredictable and depends on multiple factors.

Because our database did not contain direct information on medical conditions, we also estimated the presence of comorbid conditions using drugs collected ≤12 months prior to the initial DOAC prescription fill (Supplementary Tables S3–S5). We have therefore missed all conditions for which no pharmaceutical treatment was dispensed. Pooling patients without comorbid conditions with those diagnosed but not receiving pharmaceutical treatment may have weakened the associations for predictors of switching events. Additionally, we were unable to determine if the selected DOAC dose was off-label or identify reasons for switching anticoagulants, such as patient preference, thromboembolic or bleeding events, side effects, or changes in kidney or liver function. These limitations complicate translating our findings to clinical practice.

## 5 Conclusion

In the Netherlands, FⅩa inhibitors are increasingly being selected for OAC-naive AF patients, while dabigatran’s use is declining. Seven in 100 of such patients switch to a different OAC within 1 year, and the initial DOAC type and dose are strong predictors of such events.
