# Supplementary material for: Prescription and switching patterns of direct oral anticoagulants in patients with atrial fibrillation
Source: Res Pract Thromb Haemost. 2024 Aug 9;8(6):102544. doi: 10.1016/j.rpth.2024.102544 (PMC11404132; doi:10.1016/j.rpth.2024.102544)
Supplement: Supporting Information [file mmc1.pdf]

Supporting Information

Prescription and Switching Patterns of Direct Oral Anticoagulants in Patients With Atrial Fibrillation

August 25, 2024

de Vries T.A.C. & Bavalia R. et al

Table of contents

Table S1. STROBE Statement — checklist of items that should be included in reports of observational studies 4

Table S2. Standard and reduced doses of direct oral anticoagulants 9

Table S3. Definitions of medication classes used in the 12 months before or on the same day as the initial direct oral anticoagulant prescription collection 10

Table S4. Definitions of comorbidities at baseline as defined by medications classes used at or in the 12 months prior to the initial direct oral anticoagulant prescription collection 13

Table S5. Definitions of the categories used to estimate the modified CHA_2DS_2-VASc score 15

Figure S1. Decision tree to estimate the indication for the initially dispensed direct oral anticoagulant prescription 16

Table S6. Detailed description of the employed statistical analyses 17

Table S7. Concomitant drug groups used in the 12 months before or on the same day as the initial direct oral anticoagulant prescription collection in the cross-sectional analysis cohort 20

Table S8. Comedication groups used in the 12 months before or on the same day as the initial direct oral anticoagulant prescription collection in the longitudinal cohort 22

Table S9. Baseline characteristics of patients in the longitudinal analysis cohort 24

Figure S2. Changes in the collection of direct oral anticoagulants over time across different subgroups 26

A. Stratified by age and initially collected direct oral anticoagulant type 26

B. Stratified by sex category and initially collected DOAC type 27

Figure S3. Twelve- and six-month cumulative incidence of switching to a different oral anticoagulant 28

A. In the first 12 months of follow-up 28

B. In the first 6 months of follow-up 29

Figure S4. One-year cumulative incidence of switching to a different oral anticoagulant in different subgroups 30

A. Stratified by age 30

I. 75 years of age or older 30

II. 65 up to 75 years of age 31

III. Up to 65 years of age 32
B. Stratified by sex category 33
I. Male patients 33
II. Female patients 34
Figure S5. One-year cumulative incidences of switching among the individual direct oral anticoagulants subgroups 35
Figure S6. Adjusted and unadjusted predictors of incident switching events within the first 12 months 36
A. Switching to a different direct oral anticoagulant 36
B. Switching to a vitamin K antagonist 38
Figure S7. Predictors of switching to a different direct oral anticoagulant within the first 12 months with age fitted with a restricted cubic spline function 40
Figure S8. The adjusted nonlinear relationship between age and switching to a different direct oral anticoagulant as fitted with a restricted cubic spline function 41
Figure S9. Adjusted and unadjusted predictors of incident switching events within the first 12 months, stratified by initially collected DOAC type 42
A. Switching to a different direct oral anticoagulant 42
I. Apixaban 42
II. Dabigatran 44
III. Edoxaban 45
IV. Rivaroxaban 46
B. Switching to a vitamin K antagonist 47
I. Apixaban 47
II. Dabigatran 48
III. Edoxaban 49
IV. Rivaroxaban 50
References 51

Table S1. STROBE Statement — checklist of items that should be included in reports of observational studies

	Item No	Recommendation	Check
**Title and abstract**	1	(*a*) Indicate the study's design with a commonly used term in the title or the abstract	✓
		(*b*) Provide in the abstract an informative and balanced summary of what was done and what was found	✓
**Introduction**			
Background/rationale	2	Explain the scientific background and rationale for the investigation being reported	✓
Objectives	3	State specific objectives, including any prespecified hypotheses	✓
**Methods**			
Study design	4	Present key elements of study design early in the paper	✓
Setting	5	Describe the setting, locations, and relevant dates, including periods of recruitment, exposure, follow-up, and data collection	✓
Participants	6	(*a*) *Cohort study*—Give the eligibility criteria, and the sources and methods of selection of participants. Describe methods of follow-up<br>*Case-control study*—Give the eligibility criteria, and the sources and methods of case ascertainment and control selection. Give the rationale for the choice of cases and controls<br>*Cross-sectional study*—Give the eligibility criteria, and the sources and methods of selection of participants	✓

		(*b*) *Cohort study*—For matched studies, give matching criteria and number of exposed and unexposed<br><br>*Case-control study*—For matched studies, give matching criteria and the number of controls per case	NA
Variables	7	Clearly define all outcomes, exposures, predictors, potential confounders, and effect modifiers. Give diagnostic criteria, if applicable	✓
Data sources/ measurement	8*	For each variable of interest, give sources of data and details of methods of assessment (measurement). Describe comparability of assessment methods if there is more than one group	✓
Bias	9	Describe any efforts to address potential sources of bias	✓
Study size	10	Explain how the study size was arrived at	✓
Quantitative variables	11	Explain how quantitative variables were handled in the analyses. If applicable, describe which groupings were chosen and why	✓
Statistical methods	12	(*a*) Describe all statistical methods, including those used to control for confounding	✓[a]
		(*b*) Describe any methods used to examine subgroups and interactions	✓[a]
		(*c*) Explain how missing data were addressed	✓[b]
		(*d*) *Cohort study*—If applicable, explain how loss to follow-up was addressed<br><br>*Case-control study*—If applicable, explain how matching of cases and controls was addressed	✓[b]

		Cross-sectional study—If applicable, describe analytical methods taking account of sampling strategy	
		(*e*) Describe any sensitivity analyses	✓[a]
**Results**			
Participants	13*	(a) Report numbers of individuals at each stage of study—eg numbers potentially eligible, examined for eligibility, confirmed eligible, included in the study, completing follow-up, and analysed	✓
		(b) Give reasons for non-participation at each stage	✓
		(c) Consider use of a flow diagram	✓
Descriptive data	14*	(a) Give characteristics of study participants (eg demographic, clinical, social) and information on exposures and potential confounders	✓
		(b) Indicate number of participants with missing data for each variable of interest	NA[b]
		(c) *Cohort study*—Summarise follow-up time (eg, average and total amount)	NA[c]
Outcome data	15*	*Cohort study*—Report numbers of outcome events or summary measures over time	✓
		Case-control study—Report numbers in each exposure category, or summary measures of exposure	NA
		Cross-sectional study—Report numbers of outcome events or summary measures	✓
Main results	16	(*a*) Give unadjusted estimates and, if applicable, confounder-adjusted estimates and their precision (eg, 95% confidence interval). Make clear which	✓

		confounders were adjusted for and why they were included	
		(*b*) Report category boundaries when continuous variables were categorized	✓
		(*c*) If relevant, consider translating estimates of d into absolute risk for a meaningful time period	✓d
Other analyses	17	Report other analyses done—eg analyses of subgroups and interactions, and sensitivity analyses	✓
**Discussion**			
Key results	18	Summarise key results with reference to study objectives	✓
Limitations	19	Discuss limitations of the study, taking into account sources of potential bias or imprecision. Discuss both direction and magnitude of any potential bias	✓
Interpretation	20	Give a cautious overall interpretation of results considering objectives, limitations, multiplicity of analyses, results from similar studies, and other relevant evidence	✓
Generalisability	21	Discuss the generalisability (external validity) of the study results	✓
**Other information**			
Funding	22	Give the source of funding and the role of the funders for the present study and, if applicable, for the original study on which the present article is based	✓

Note: An Explanation and Elaboration article discusses each checklist item and gives methodological background and published examples of transparent reporting. The STROBE checklist is best used in conjunction with this article (freely available on the Web sites of PLoS Medicine at http://www.plosmedicine.org/, Annals of Internal Medicine at http://www.annals.org/, and Epidemiology at http://www.epidem.com/). Information on the STROBE Initiative is available at www.strobe-statement.org [1].

STROBE Strengthening the Reporting of Observational Studies in Epidemiology; *NA* Not Applicable.

[*] Give information separately for cases and controls in case-control studies and, if applicable, for exposed and unexposed groups in cohort and cross-sectional studies;

[a] A more detailed description of the employed statistical analyses is provided in **Table S6**; [b] By design, we had no missing data because we excluded all patients who collected their prescriptions from pharmacies that did not provide complete data during the entire study duration, and because we defined discontinuation of oral anticoagulant treatment as the absence a refill of any oral anticoagulant prescription for 12 consecutive months; [c] As mentioned in the methods all patients were followed for 12 months, with an additional 12 months to ascertain if patients discontinued therapy with any oral anticoagulant; [d] We did not report absolute risk differences, but we did provide the absolute 6- and 12-month cumulative incidences of switching events for each subgroup of patients in the forest plot figures.

Table S2. Standard and reduced doses of direct oral anticoagulants

DOAC type	Standard dose	Reduced or lower dose[a]
Apixaban	5 mg twice daily	2.5 mg twice daily
Dabigatran	150 mg twice daily	Not applicable, but using the lower standard dose in selected patients at risk of bleeding is recommended
	110 mg twice daily	
Edoxaban	60 mg once daily	30 mg once daily
Rivaroxaban	20 mg once daily	15 mg once daily

The definitions used for standard and reduced dose direct oral anticoagulants in line with current European guidance documents [2].

DOAC direct oral anticoagulant.

[a] Apixaban, edoxaban, and rivaroxaban are available for use in patients with atrial fibrillation in a standard dose together with a reduced dose for selected patients at higher risk of bleeding [2,3]. In contrast, dabigatran is available in two standard doses, a higher and a lower dose [2,3]. Both doses were directly compared to a VKA in the RELY trial and were subsequently approved for use in clinical practice [2-4]. It was only after later analyses of the trial results that regulatory authorities updated their recommendation to consider using the lower dose in selected patients at elevated risk of bleeding [2]. To allow for direct comparisons across the four DOACs, we considered the higher dabigatran dose to be analogous to the standard doses and its lower dose to the reduced doses of the other DOACs.

Table S3. Definitions of medication classes used in the 12 months before or on the same day as the initial direct oral anticoagulant prescription collection

**Medical classes**	**Definition**
Antidiabetics	A10 class (Drugs used in diabetes)
Platelet aggregation inhibitors	B1C class (Platelet aggregation inhibitors)
Cardiac glycosides	Complete C1A1 class (Plain cardiac glycosides)
Anti-arrhythmic drugs	Complete C1B class (anti-arrhythmic drugs)
	Sotalol[a]
Nitrites/nitrates	C1E class (Nitrites and nitrates)
Antihypertensives (other)	C2 class (Antihypertensives)
Diuretics	C3 class (Diuretics)
	C7B1 (Beta-blocking agents, combinations with antihypertensives and/or diuretics), whenever including diuretics
	C8B1 (Calcium-antagonists, combinations with antihypertensives and/or diuretics), whenever including diuretics
	C9B1 (ACE inhibitors, combinations with antihypertensives and/or diuretics), whenever including diuretics
	C9B3 (ACE inhibitors, combinations with calcium antagonists), some included indapamide
	C9D1 (Angiotensin-II antagonists, combinations with antihypertensives and/or diuretics), whenever including diuretics
	C9D3 (Angiotensin-II antagonist combinations with calcium antagonists), some included hydrochlorothiazide
	C9X (Other agents acting on the renin-angiotensin system), whenever including diuretics

Medical classes	Definition
Betablockers (rate control)[b]	C7A (beta-blockings agents, plain), whenever including metoprolol, bisoprolol, atenolol, nebivolol, carvedilol, esmolol and/or atenolol
	C7B1 (beta-blockings agents, combinations with antihypertensives and/or diuretics), whenever including metoprolol, bisoprolol, atenolol, nebivolol, carvedilol, esmolol and/or atenolol
	C9B2 (ACE inhibitor/beta-blocker combinations), whenever including metoprolol, bisoprolol, atenolol, nebivolol, carvedilol, esmolol and/or atenolol
Betablockers (other)	Propanolol[a], labetolol[a], celiprolol[a], acebutolol[a], and/or pindolol[a]
Non-dihydropyridines calcium antagonists [b]	C8A (Calcium antagonist, plain), but not verapamil and/or diltiazem
	C9B3 (ACE inhibitors, combinations with calcium antagonists), whenever including verapamil and/or diltiazem
Dihydropyridines calcium antagonists	C8A (Calcium antagonist, plain), but not verapamil and/or diltiazem
	C8B (Calcium antagonist, combinations), but not verapamil and/or diltiazem
	C9D3 (Angiotensin-II antagonist combinations with calcium antagonists), but not verapamil and/or diltiazem
RAAS inhibitors	C9A (ACE inhibitors, plain)
	C9B1 (ACE inhibitor combinations with antihypertensives and/or diuretics)
	C9B2 (ACE inhibitor/beta-blocker combinations) in presence of perindopril
	C9B3 (ACE inhibitor combinations with calcium antagonists) whenever including perindopril, enalapril and/or trandolapril
	C9C (Angiotensin-II antagonists, plain)

Medical classes	Definition
	C9D1 (Angiotensin-II antagonist combinations with antihypertensives and/or diuretics)
	C9D3 (Angiotensin-II antagonist combinations with calcium antagonists)
	C9D9 (Angiotensin-II antagonist combinations with other drugs)
	C9X (Other agents acting on the renin-angiotensin system)
Anti-Parkinson drugs	N4 (Anti-Parkinson drugs)
Psycholeptics	N5 (Psycholeptics)[c]
Psychoanaleptics	N6 (Psychoanaleptics excluding anti-obesity preparations)[d]

Used definitions were based on the European Pharmaceutical Market Research Association Anatomical Classification Guidelines 2018 or individual molecules where required. These drug classes were used to estimate both the comorbidities (**Table S4**) and the modified CHA_2DS_2-VASc score at baseline (**Table S5**).

[a] Individual molecule; [b] Drugs commonly used for rate control in patients with atrial fibrillation [5]; [c] Major drug classes in this group are antipsychotics, hypnotics and sedatives, and tranquilizers; [d] Major drug classes in this group are anti-depressants and mood stabilizers, psychostimulants, nootropics, and neurotonics.

ACE angiotensin converting enzyme; *RAAS* renin-angiotensin-aldosterone system.

Table S4. Definitions of comorbidities at baseline as defined by medications classes used at or in the 12 months prior to the initial direct oral anticoagulant prescription collection

Comorbidities	Medication classes
Diabetes mellitus	Antidiabetics
Composite of hypertension and congestive heart failure	Diuretics; AND/OR
	RAAS inhibitors; AND/OR
	Betablockers (other); AND/OR
	Dihydropyridines calcium antagonists; AND/OR
	Antihypertensives (other)
Atherosclerotic disease	Platelet aggregation inhibitors; AND/OR
	Nitrates/nitrates
Clinical pattern of atrial fibrillation	
Persistent or paroxysmal[a]	Anti-arrhythmic drugs
Permanent[a]	Betablockers (rate control) in absence of anti-arrhythmic drugs; AND/OR
	Non-dihydropyridines calcium antagonists in absence of anti-arrhythmic drugs; AND/OR
	Cardiac glycosides in absence of anti-arrhythmic drugs
Uncertain pattern	Absence of anti-arrhythmic drugs, betablockers (rate control), and non-dihydropyridines calcium antagonists
Parkinson’s disease	Anti-Parkinson drugs
Mental disorders necessitating treatment with psycholeptics (e.g., sleeping, anxiety, and psychotic disorders)	Psycholeptics
Mental disorders necessitating treatment with psychoanaleptics (e.g., depressive and bipolar disorders)	Psychoanaleptics

This table describes the definitions of comorbidities at baseline as defined by medications classes used at or in the 12 months prior to the initial DOAC prescription collection. Definitions of the medication classes are presented in **Table S3**.

RAAS renin-angiotensin-aldosterone system.

[a] We categorized the pattern of atrial fibrillation into persistent or paroxysmal (i.e., use of antiarrhythmic drugs), permanent (i.e., use of drugs recommended for controlling the heart rate in AF in absence of any antiarrhythmic drugs), and unclear pattern (i.e., any other combination) [5]. The drug classes used to differentiate between persistent/paroxysmal and permanent atrial fibrillation are sometimes also indicated for the prevention of other cardiac arrhythmias, which include supraventricular (e.g., atrial tachycardias or atrioventricular nodal reentry tachycardia) and ventricular arrhythmias (e.g., ventricular fibrillation or sustained ventricular tachycardia).

Table S5. Definitions of the categories used to estimate the modified CHA_2DS_2-VASc score

Letter(s)	Characteristic(s)	Point(s) if present
CH	**C**ongestive heart failure, or **H**ypertension	1
	Diuretics; OR	
	RAAS inhibitors; OR	
	Betablockers, other; OR	
	Calcium antagonists, other; OR	
	Other antihypertensives	
A_2	**A**ge (≥75 years)	2
D	**D**iabetes mellitus	1
	Antidiabetics	
S_2V	**S**troke/TIA, or **V**ascular disease	2
	Platelet aggregation inhibitors; OR	
	Nitrates/nitrates	
A	**A**ge 65-74 years	1
Sc	**S**ex **c**ategory (female)	1

This table describes all categories and their corresponding score to calculate a modified CHA_2DS_2-VASc score. We used the same definitions to estimate the comorbidities at baseline (**Table S3-S4**). In contrast with the official CHA_2DS_2-VASc score, we combined 'Congestive heart failure' with 'Hypertension' as medication classes used in our definition are used for both indications. We also combined the 'Stroke/TIA' with the 'Vascular disease' category as antiplatelet therapy can be prescribed for either indication. Consequently, our modified score has a maximum of 7 points (2 less than the maximum of the official CHA_2DS_2-VASc score).

RAAS renin-angiotensin-aldosterone system; *TIA* transient ischemic attack.

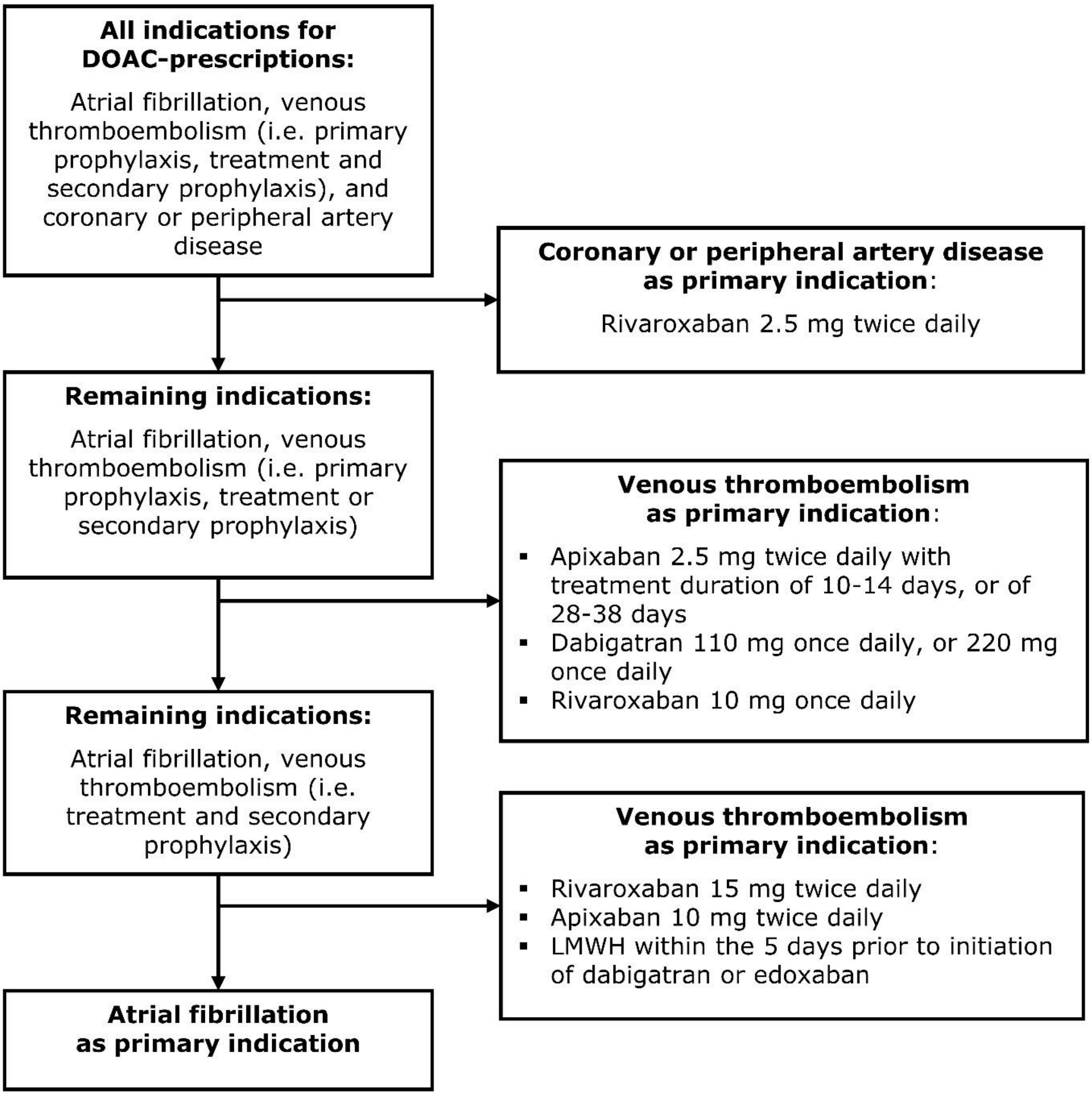

Figure S1. Decision tree to estimate the indication for the initially dispensed direct oral anticoagulant prescription

Data on approved strategies were derived from comprehensive narrative review [6], the *European Medicines Agency* (EMA) assessment document [3], and guidance documents on (i) atrial fibrillation [2,5], (ii) chronic coronary syndromes [7], (iii) primary prevention of venous thromboembolism [8], and (iv) treatment and secondary prevention of venous thromboembolism [9]. We confirmed the identified strategies by assessing the '*Farmacotherapeutisch Kompas*' webpage, a government website on drugs available in the Netherlands for human patients as registered at the '*College ter Beoordeling van Geneesmiddelen*' (CBG) or the EMA [10-15].

LMWH low-molecular weight heparin.

Table S6. Detailed description of the employed statistical analyses

Analysis	Description
Summarizing baseline characteristics	To summarize the baseline patient characteristics of patients in either analysis cohort, we used summary statistics appropriate for expected distribution of the data, both irrespective and categorized into four subgroups defined by the initially dispensed DOAC type. We used the mean with standard deviation for ratio or interval variables if normally distributed, and with median and interquartile range if not. Ordinal variables were summarized with median and interquartile range or number and proportion of patients. Nominal variables were presented as the number and proportion of patients.
Assessing for patterns in initial DOAC prescriptions over time	To describe the patterns in the selection of initial DOAC prescriptions over time, we determined the total number of patients who collected a DOAC in each month of the study period, both irrespective of and stratified by initially collected DOAC type. We also described these patterns by age (i.e., ≥75, ≥65 to <75, and <65 years) and sex categories.
Determining the cumulative incidence of switching to a different DOAC or a VKA and of discontinuations of the initially collected DOAC without substitution with another OAC	To determine the one-year cumulative incidence of switching to a different DOAC, we calculated the number and proportion of patients who switched from their initial DOAC to another DOAC within the first year after commencing treatment, and we did the same for switching to a VKA. In both analyses we only considered incident events. We determined the 95% confidence intervals (95%-CIs) using the Agresti-Coull interval method [16]. To assess for consistency in our findings, we repeated these sets of analyses but instead determined the six-month cumulative incidence. We also determined the one-year cumulative incidence stratified by age (i.e., ≥75, ≥65 to <75, and <65 years) and sex categories.

Analysis	Description
Identifying predictors of switching events	To identify potential predictors of switching to a different OAC within one year, we performed univariable and multivariable robust Poisson regression analyses [17,18]. Robust standard errors and 95%-CIs were estimated with the fifth heteroskedasticity-consistent formula (i.e., HC5) [18,19]. For ratio or interval predictors, we assessed if their relationship with each outcome of interest was nonlinear by using a restricted cubic spline function [20]. The fit of more complex nonlinear relationships were assessed by iteratively increasing the number of knots of the spline function. Knot locations were determined solely by the number of knots [21]. We defined the model with the best fit as the one with the lowest Bayesian Information Criterion value [22]. We created two models if age (the only continuous co-variable of interest) had a nonlinear relationship with the outcome to facilitate the interpretation of the models, one with age modelled with a restricted cubic spline function [20,21], and another with age categorized in accordance with the knot locations of the optimal fit. Interactions between predictors were not considered.<br><br>To reduce the risk of overfitting, we selected potential predictors based solely on available literature [23-27], or clinical rationale and then applied the full model approach.[28] However, to avoid high multicollinearity among some of the predictors [29], we created at least two full-fit models per outcome of interest, one with all preselected variables included except the modified CHA_2DS_2-VASc score and another with this score included but omitting the underlying covariables (i.e., age, sex category, and the three variables on cardiovascular comorbidities).<br><br>We did not consider the events per degrees of freedom ratio in any of these analyses because we anticipated this ratio to be well above any proposed cutoff due to our large sample size and the anticipated incidence of switching events based on prior reports [23-28].

Analysis	Description
	We performed a post-hoc defined subgroup analysis identifying potential predictors of switching in each subgroup of initially collected DOAC type.
Other comments	All analyses were performed in R version 4.2.2 on a Windows device [30].

This table provides a detailed description of the performed statistical analyses.

DOAC direct oral anticoagulant, *CI* confidence interval, *VKA* vitamin K antagonist, *HC5* fifth heteroskedasticity-consistent formula.

Table S7. Concomitant drug groups used in the 12 months before or on the same day as the initial direct oral anticoagulant prescription collection in the cross-sectional analysis cohort

		Stratified by initially collected DOAC type			
	**Total (*n* = 94,874)**	**Apixaban (*n* = 33,788)**	**Dabigatran (*n* = 19,326)**	**Edoxaban (*n* = 8,596)**	**Rivaroxaban (*n* = 33,164)**
Cardiovascular comedication					
Antidiabetics	14,509 (15.3%)	5,320 (15.7%)	2,745 (14.2%)	1,310 (15.2%)	5,134 (15.5%)
Platelet aggregation inhibitors	35,451 (37.4%)	13,006 (38.5%)	7,091 (36.7%)	3,229 (37.6%)	12,125 (36.6%)
Nitrites or nitrates	9,033 (9.5%)	3,448 (10.2%)	1,641 (8.5%)	818 (9.5%)	3,126 (9.4%)
Glycosides	8,824 (9.3%)	3,323 (9.8%)	1,759 (9.1%)	781 (9.1%)	2,961 (8.9%)
Anti-arrhythmic drugs (non-sotalol)	8,959 (9.4%)	3,298 (9.8%)	1,926 (10.0%)	687 (8.0%)	3,048 (9.2%)
Sotalol	89,934 (94.8%)	32,246 (95.4%)	18,148 (93.9%)	8,134 (94.6%)	31,406 (94.7%)
Betablocking agents (rate control)	55,931 (59.0%)	19,889 (58.9%)	12,016 (62.2%)	5,157 (60.0%)	18,869 (56.9%)
Betablocking agents (other)	1,476 (1.6%)	578 (1.7%)	294 (1.5%)	104 (1.2%)	500 (1.5%)
Dihydropyridines calcium antagonists	19,331 (20.4%)	7,106 (21.0%)	3,808 (19.7%)	1,772 (20.6%)	6,645 (20.0%)
Non-dihydropyridines calcium antagonists	4,212 (4.4%)	1,472 (4.4%)	800 (4.1%)	361 (4.2%)	1,579 (4.8%)

	Total (*n* = 94,874)	Stratified by initially collected DOAC type			
		Apixaban (*n* = 33,788)	Dabigatran (*n* = 19,326)	Edoxaban (*n* = 8,596)	Rivaroxaban (*n* = 33,164)
Diuretics	34,095 (35.9%)	12,665 (37.5%)	6,660 (34.5%)	3,077 (35.8%)	11,693 (35.3%)
RAASi	24,846 (26.2%)	9,141 (27.1%)	4,982 (25.8%)	2,240 (26.1%)	8,483 (25.6%)
Antihypertensives (other)	1,461 (1.5%)	553 (1.6%)	270 (1.4%)	148 (1.7%)	490 (1.5%)
Psychopharmaceuticals					
Anti-Parkinson drugs	1,599 (1.7%)	582 (1.7%)	277 (1.4%)	137 (1.6%)	603 (1.8%)
Psycholeptics	18,364 (19.4%)	6,605 (19.5%)	3,462 (17.9%)	1,679 (19.5%)	6,618 (20.0%)
Psychoanaleptics	9,887 (10.4%)	3,588 (10.6%)	1,851 (9.6%)	860 (10.0%)	3,588 (10.8%)

This table describes the number (%) of patients in the cross-sectional analysis cohort who collected specific comedication in the 12 months before or on the same day as the initial DOAC prescription collection. The definition of each co-medication group is presented in **Table S3**. Note that the subclasses of drugs do not always add up as patients could be treated with multiple drugs within each class. For instance, a patient being treated with a betablocking agent used for rate control could also have used a betablocker not associated with rate control in the defined period.

DOAC direct oral anticoagulant; *RAASi* renin-angiotensin-aldosterone system inhibitor.

Table S8. Comedication groups used in the 12 months before or on the same day as the initial direct oral anticoagulant prescription collection in the longitudinal cohort

		Stratified by initially collected DOAC type			
	**Total (*n* = 66,090)**	**Apixaban (*n* = 21,962)**	**Dabigatran (*n* = 16,066)**	**Edoxaban (*n* = 5,028)**	**Rivaroxaban (*n* = 23,034)**
Cardiovascular comedication					
Antidiabetics	10,131 (15.3%)	3,553 (16.2%)	2,314 (14.4%)	733 (14.6%)	3,531 (15.3%)
Platelet aggregation inhibitors	25,147 (38.0%)	8,644 (39.4%)	6,013 (37.4%)	1,922 (38.2%)	8,568 (37.2%)
Nitrites or nitrates	6,308 (9.5%)	2,224 (10.1%)	1,504 (9.4%)	442 (8.8%)	2,138 (9.3%)
Glycosides	6,763 (10.2%)	2,352 (10.7%)	1,684 (10.5%)	462 (9.2%)	2,265 (9.8%)
Anti-arrhythmic drugs (non-sotalol)	3,765 (5.7%)	1,116 (5.1%)	1,017 (6.3%)	279 (5.5%)	1,353 (5.9%)
Sotalol	6,340 (9.6%)	2,287 (10.4%)	1,388 (8.6%)	480 (9.5%)	2,185 (9.5%)
Betablocking agents (rate control)	23,911 (36.2%)	8,409 (38.3%)	5,603 (34.9%)	1,770 (35.2%)	8,129 (35.3%)
Betablocking agents (other)	39,351 (59.5%)	13,065 (59.5%)	10,063 (62.6%)	3,001 (59.7%)	13,222 (57.4%)
Dihydropyridines calcium antagonists	1,037 (1.6%)	381 (1.7%)	248 (1.5%)	63 (1.3%)	345 (1.5%)
Non-dihydropyridines calcium antagonists	13,341 (20.2%)	4,629 (21.1%)	3,117 (19.4%)	1,001 (19.9%)	4,594 (19.9%)

Diuretics	3,076 (4.7%)	1,004 (4.6%)	703 (4.4%)	231 (4.6%)	1,138 (4.9%)
RAASi	17,319 (26.2%)	5,969 (27.2%)	4,155 (25.9%)	1,275 (25.4%)	5,920 (25.7%)
Antihypertensives (other)	1,018 (1.5%)	364 (1.7%)	227 (1.4%)	81 (1.6%)	346 (1.5%)
Psychopharmaceuticals					
Anti-Parkinson drugs	1,125 (1.7%)	376 (1.7%)	227 (1.4%)	76 (1.5%)	446 (1.9%)
Psycholeptics	12,837 (19.4%)	4,358 (19.8%)	2,908 (18.1%)	942 (18.7%)	4,629 (20.1%)
Psychoanaleptics	6,829 (10.3%)	2,315 (10.5%)	1,543 (9.6%)	477 (9.5%)	2,494 (10.8%)

This table describes the number (%) of patients in the longitudinal analysis cohort who collected specific comedication in the 12 months before or on the same day as the initial DOAC prescription collection. The definition of each co-medication group is presented in **Table S3**. Note that the subclasses of drugs do not always add up as patients could be treated with multiple drugs within each class. For instance, a patient being treated with a betablocking agent used for rate control could also have used a betablocker not associated with rate control in the defined period.

DOAC direct oral anticoagulant; *RAASi* renin-angiotensin-aldosterone system inhibitor.

Table S9. Baseline characteristics of patients in the longitudinal analysis cohort

		Stratified by initially collected DOAC type			
	Total (*n* = 66,090)	Apixaban (*n* = 21,962)	Dabigatran (*n* = 16,066)	Edoxaban (*n* = 5,028)	Rivaroxaban (*n* = 23,034)
Age – years	71 [64 – 79]	72 [64 – 80]	71 (64 – 78]	71 [63 – 78]	71 [63 – 79]
≥75	25,628 (38.8%)	9,217 (42.0%)	5,903 (36.7%)	1,826 (36.3%)	8,682 (37.7%)
65–74	22,513 (34.1%)	7,131 (32.5%)	5,846 (36.4%)	1,721 (34.2%)	7,815 (33.9%)
≤64	17,949 (27.2%)	5,614 (25.6%)	4,317 (26.9%)	1,481 (29.5%)	6,537 (28.4%)
Female sex	28,889 (43.7%)	9,990 (45.5%)	6,740 (42.0%)	2,111 (42.0%)	10,048 (43.6%)
Reduced DOAC dosing regimen	11,948 (18.1%)	2,575 (11.7%)	5,023 (31.3%)	899 (17.9%)	3,451 (15.0%)
Clinical pattern of atrial fibrillation					
Permanent	37,798 (57.2%)	12,588 (57.3%)	9,578 (59.6%)	2,880 (57.3%)	12,752 (55.4%)
Persistent or paroxysmal	9,654 (14.6%)	3,166 (14.4%)	2,495 (15.5%)	684 (13.6%)	3,309 (14.4%)
Uncertain type	18,638 (28.2%)	6,208 (28.3%)	3,993 (24.9%)	1,464 (29.1%)	6,973 (30.3%)
Atherosclerotic disease	25,926 (39.2%)	8,907 (40.6%)	6,177 (38.4%)	1,981 (39.4%)	8,861 (38.5%)
Hypertension or congestive heart failure	36,402 (55.1%)	12,537 (57.1%)	8,704 (54.2%)	2,699 (53.7%)	12,462 (54.1%)
Diabetes mellitus	10,131 (15.3%)	3,553 (16.2%)	2,314 (14.4%)	733 (14.6%)	3,531 (15.3%)
Modified CHA_2DS_2VASc score	3 [2 – 4]	3 [2 – 5]	3 [2 – 4]	3 [2 – 4]	3 [2 – 4]

	Total (*n* = 66,090)	Stratified by initially collected DOAC type			
		Apixaban (*n* = 21,962)	Dabigatran (*n* = 16,066)	Edoxaban (*n* = 5,028)	Rivaroxaban (*n* = 23,034)
High risk (≥2 in males, ≥3 in females)	45,931 (69.5%)	15,711 (71.5%)	11,097 (69.1%)	3,386 (67.3%)	15,737 (68.3%)
Medium risk (1 in males, 2 in females)	11,485 (17.4%)	3,525 (16.1%)	2,911 (18.1%)	944 (18.8%)	4,105 (17.8%)
Low risk (0 in males, 1 in females)	8,674 (13.1%)	2,726 (12.4%)	2,058 (12.8%)	698 (13.9%)	3,192 (13.9%)
Parkinson's disease	1,125 (1.7%)	376 (1.7%)	227 (1.4%)	76 (1.5%)	446 (1.9%)
Mental disorders necessitating treatment with psycholeptics (e.g., sleeping, anxiety, and psychotic disorders)	12,837 (19.4%)	4,358 (19.8%)	2,908 (18.1%)	942 (18.7%)	4,629 (20.1%)
Mental disorders necessitating treatment with psychoanaleptics (e.g., depressive and bipolar disorders)	6,829 (10.3%)	2,315 (10.5%)	1,543 (9.6%)	477 (9.5%)	2,494 (10.8%)

This table summarizes the baseline characteristics of the patients in the longitudinal analysis cohort. Presented values are number (%) of patients, or median [interquartile range].

DOAC direct oral anticoagulant.

Figure S2. Changes in the collection of direct oral anticoagulants over time across different subgroups

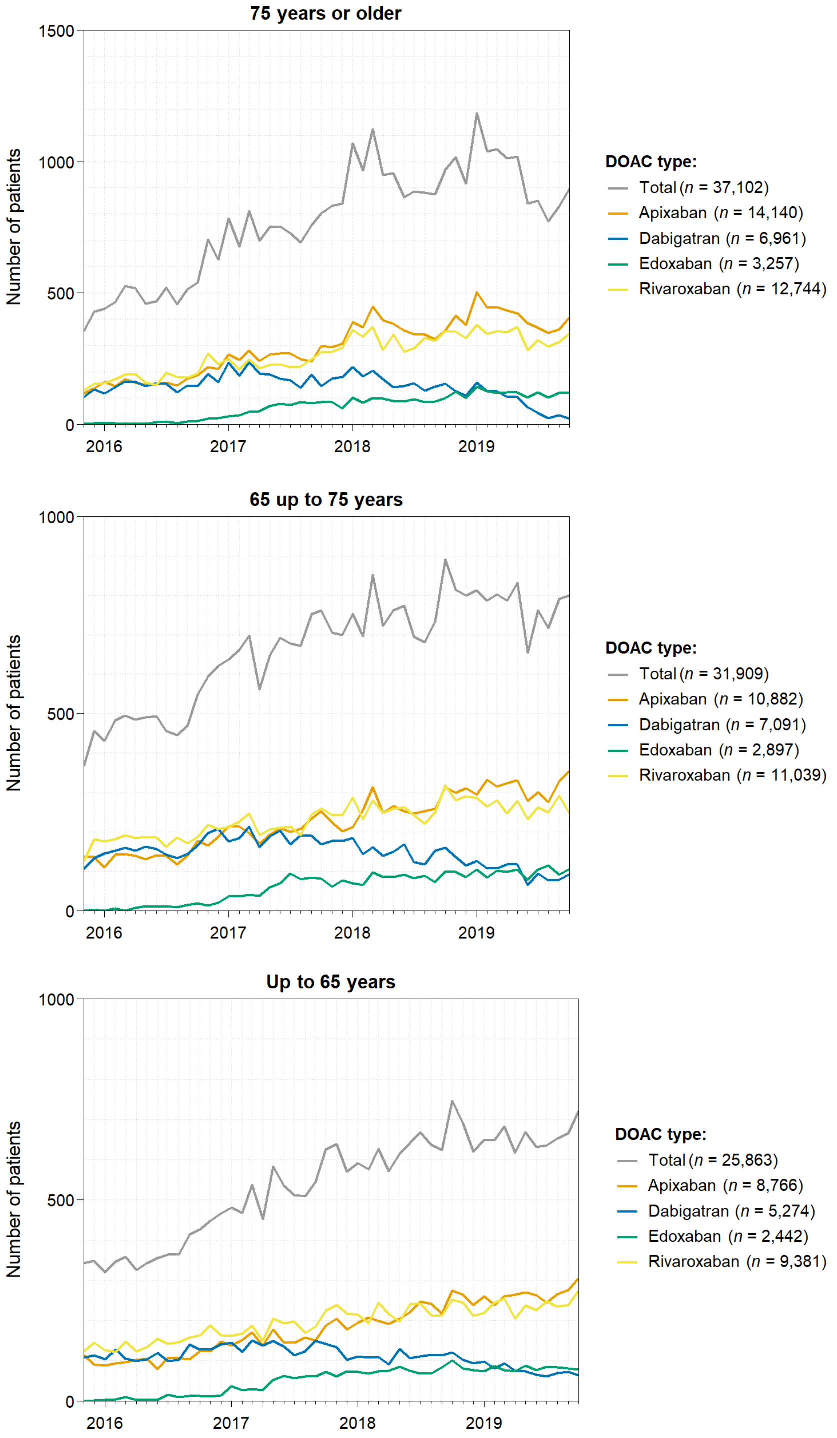

A. Stratified by age and initially collected direct oral anticoagulant type

This figure illustrates the number of oral anticoagulant naïve patients in the cross-sectional analysis cohort (*n*=94,874) per month who picked up their first DOAC prescription stratified by type of initially collected DOAC and age group.

DOACs direct oral anticoagulants.

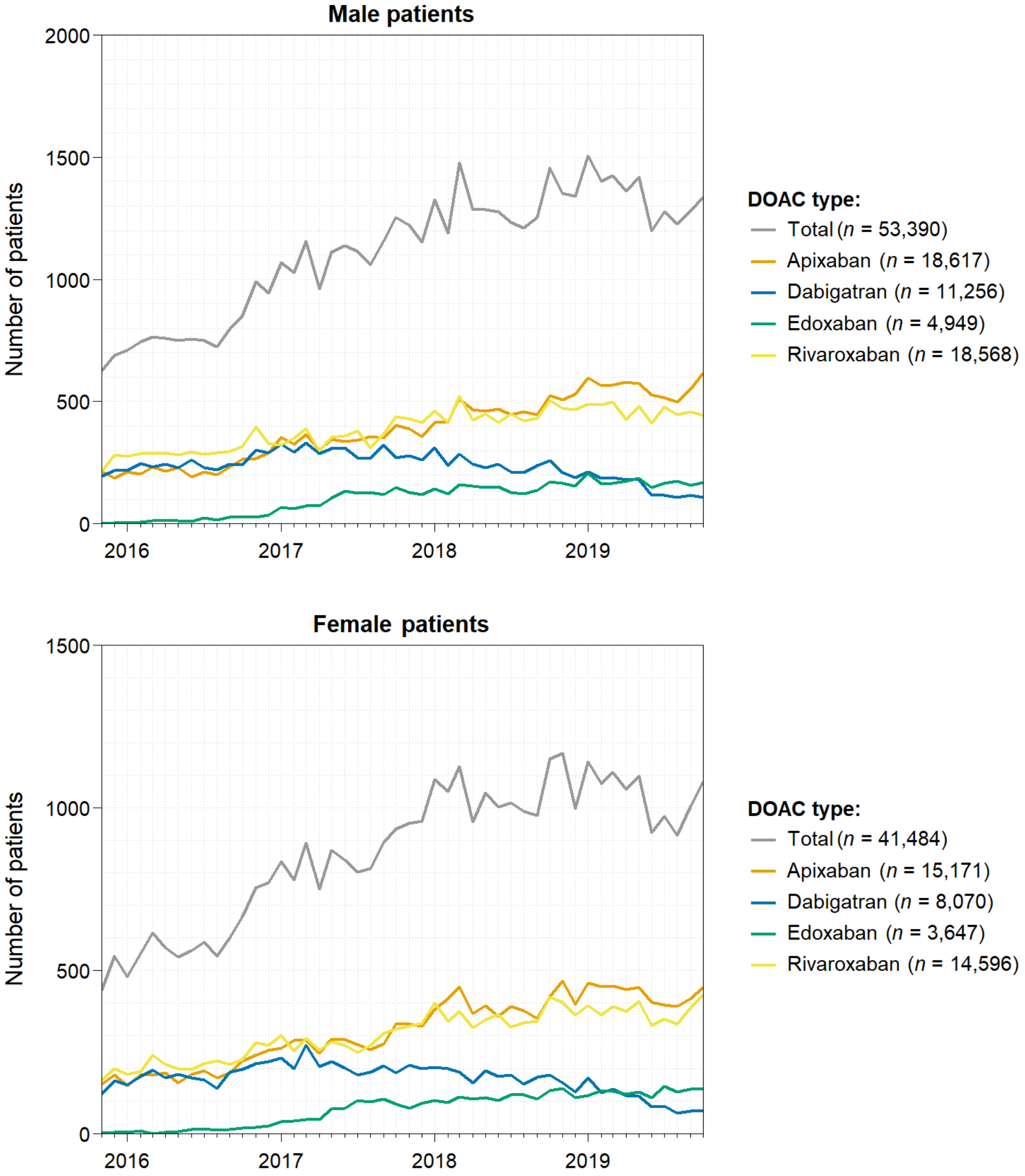

B. <u>Stratified by sex category and initially collected DOAC type</u>

This figure illustrates the number of oral anticoagulant naïve patients in the cross-sectional analysis cohort (n=94,874) per month who picked up their first DOAC prescription stratified by type of initially collected DOAC and sex category.

DOACs direct oral anticoagulants.

Figure S3. Twelve- and six-month cumulative incidence of switching to a different oral anticoagulant

Initially collected DOAC	No. of Patients	1-year cumulative incidence (95% confidence interval)
**Switched to a different DOAC**		
- Total	3,057	4.6% (4.5 - 4.8%)
- Apixaban	642	2.9% (2.7 - 3.2%)
- Dabigatran	1,371	8.5% (8.1 - 9.0%)
- Edoxaban	220	4.4% (3.8 - 5.0%)
- Rivaroxaban	824	3.6% (3.3 - 3.8%)
**Switched to a VKA**		
- Total	1,389	2.1% (2.0 - 2.2%)
- Apixaban	400	1.8% (1.7 - 2.0%)
- Dabigatran	328	2.1% (1.8 - 2.3%)
- Edoxaban	98	2.0% (1.6 - 2.4%)
- Rivaroxaban	563	2.5% (2.3 - 2.7%)
**Discontinued use of any OAC**		
- Total	16,359	24.8% (24.4 - 25.1%)
- Apixaban	5,635	25.7% (25.1 - 26.2%)
- Dabigatran	2,952	18.4% (17.8 - 19.0%)
- Edoxaban	1,421	28.3% (27.0 - 29.5%)
- Rivaroxaban	6,351	27.6% (27.0 - 28.2%)
**Continued use of the initially collected DOAC**		
- Total	45,285	68.5% (68.2 - 68.9%)
- Apixaban	15,285	69.6% (69.0 - 70.2%)
- Dabigatran	11,415	71.0% (70.3 - 71.7%)
- Edoxaban	3,289	65.4% (64.1 - 66.7%)
- Rivaroxaban	15,296	66.4% (65.8 - 67.0%)

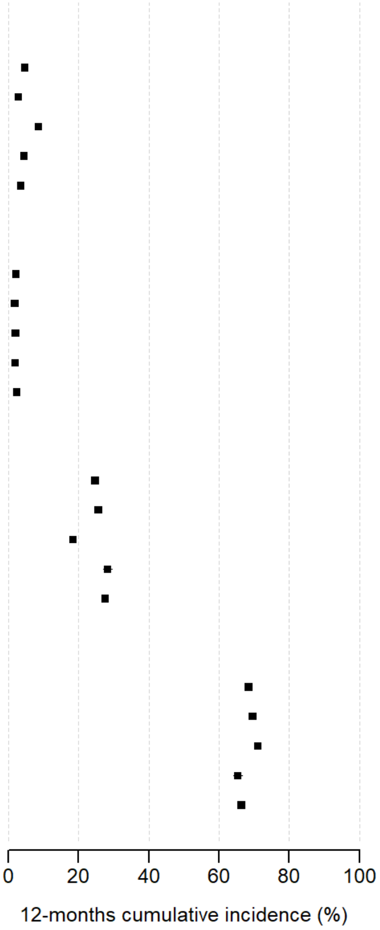

A. <u>In the first 12 months of follow-up</u>

This figure illustrates the twelve-month cumulative incidences of switching to a different OAC in the longitudinal analysis cohort (*n*=66,090) stratified by initially collected DOAC type. The sum of all four treatment statuses is 100% for each subgroup (i.e., total, apixaban, dabigatran, edoxaban, rivaroxaban).

DOAC direct oral anticoagulant; *OAC* oral anticoagulant; *VKA* vitamin K antagonist.

B. First 6 months (*n* = 66,090)

Initially collected DOAC	No. of Patients	6-month cumulative incidence (95% confidence interval)
**Switched to a different DOAC**		
- Total	2,075	3.1% (3.0 - 3.3%)
- Apixaban	427	2.0% (1.8 - 2.1%)
- Dabigatran	928	5.8% (5.4 - 6.1%)
- Edoxaban	144	2.9% (2.4 - 3.4%)
- Rivaroxaban	576	2.5% (2.3 - 2.7%)
**Switched to a VKA**		
- Total	982	1.5% (1.4 - 1.6%)
- Apixaban	276	1.3% (1.1 - 1.4%)
- Dabigatran	220	1.4% (1.2 - 1.6%)
- Edoxaban	67	1.4% (1.0 - 1.7%)
- Rivaroxaban	419	1.8% (1.7 - 2.0%)
**Discontinued use of any OAC**		
- Total	12,444	18.8% (18.5 - 19.1%)
- Apixaban	4,243	19.3% (18.8 - 19.8%)
- Dabigatran	2,057	12.8% (12.3 - 13.3%)
- Edoxaban	1,100	21.9% (20.8 - 23.0%)
- Rivaroxaban	5,044	21.9% (21.4 - 22.4%)
**Continued use of the initially collected DOAC**		
- Total	50,589	76.5% (76.2 - 76.9%)
- Apixaban	17,016	77.5% (76.9 - 78.0%)
- Dabigatran	12,861	80.0% (79.4 - 80.7%)
- Edoxaban	3,717	73.9% (72.7 - 75.1%)
- Rivaroxaban	16,995	73.8% (73.2 - 74.3%)

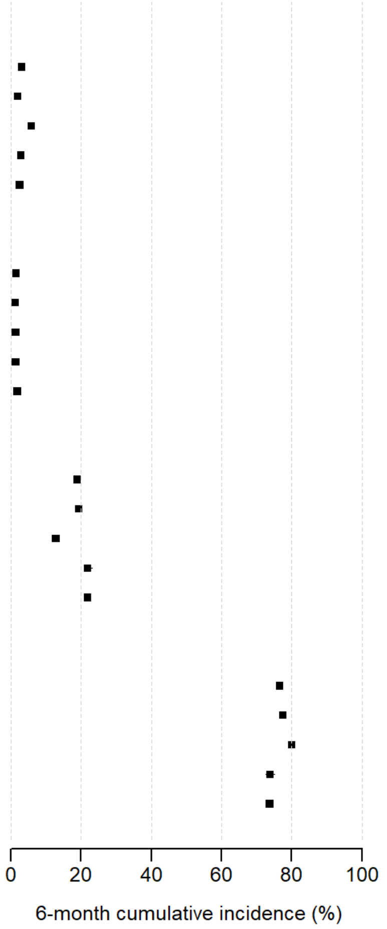

B. <u>In the first 6 months of follow-up</u>

This figure illustrates the six-month cumulative incidences of switching to a different OAC in the longitudinal analysis cohort (*n*=66,090) stratified by initially collected DOAC type. The sum of all four treatment statuses is 100% for each subgroup (i.e., total, apixaban, dabigatran, edoxaban, rivaroxaban).

DOAC direct oral anticoagulant; *OAC* oral anticoagulant; *VKA* vitamin K antagonist.

Figure S4. One-year cumulative incidence of switching to a different oral anticoagulant in different subgroups

A. Stratified by age

Initially collected DOAC	No. of Patients	1-year cumulative incidence (95% confidence interval)
**Switched to a different DOAC**		
- Total	907	3.5% (3.3 - 3.8%)
- Apixaban	204	2.2% (1.9 - 2.5%)
- Dabigatran	362	6.2% (5.5 - 6.8%)
- Edoxaban	73	4.1% (3.2 - 5.0%)
- Rivaroxaban	268	3.1% (2.7 - 3.5%)
**Switched to a VKA**		
- Total	467	1.8% (1.7 - 2.0%)
- Apixaban	133	1.5% (1.2 - 1.7%)
- Dabigatran	72	1.3% (1.0 - 1.5%)
- Edoxaban	34	2.0% (1.3 - 2.6%)
- Rivaroxaban	228	2.6% (2.3 - 3.0%)
**Discontinued use of any OAC**		
- Total	5,259	20.5% (20.0 - 21.0%)
- Apixaban	1,968	21.4% (20.5 - 22.2%)
- Dabigatran	688	11.7% (10.9 - 12.5%)
- Edoxaban	428	23.5% (21.6 - 25.4%)
- Rivaroxaban	2,175	25.1% (24.2 - 26.0%)
**Continued use of the initially collected DOAC**		
- Total	18,995	74.1% (73.6 - 74.7%)
- Apixaban	6,912	75.0% (74.1 - 75.9%)
- Dabigatran	4,781	81.0% (80.0 - 82.0%)
- Edoxaban	1,291	70.7% (68.6 - 72.7%)
- Rivaroxaban	6,011	69.2% (68.3 - 70.2%)

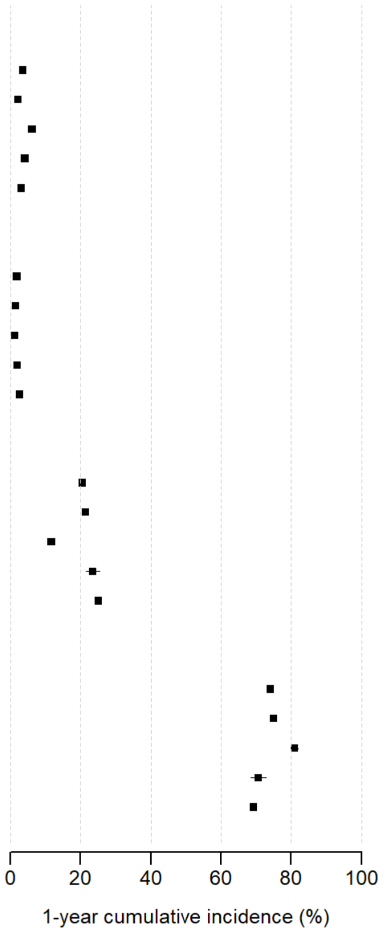

I. 75 years of age or older

This figure illustrates the one-year cumulative incidences of switching to a different OAC in the patients of 75 years of age or older (*n*=25,628) in the longitudinal analysis cohort (*n*=66,090) stratified by initially collected DOAC type. The sum of all four treatment statuses is 100% for each subgroup (i.e., total, apixaban, dabigatran, edoxaban, rivaroxaban).

DOAC direct oral anticoagulant; *OAC* oral anticoagulant; *VKA* vitamin K antagonist.

Initially collected DOAC	No. of Patients	1-year cumulative incidence (95% confidence interval)
**Switched to a different DOAC**		
- Total	1,292	5.7% (5.4 - 6.1%)
- Apixaban	244	3.4% (3.0 - 3.9%)
- Dabigatran	641	11.0% (10.2 - 11.8%)
- Edoxaban	84	5.0% (4.0 - 6.0%)
- Rivaroxaban	323	4.2% (3.7 - 4.6%)
**Switched to a VKA**		
- Total	481	2.1% (2.0 - 2.3%)
- Apixaban	137	1.9% (1.6 - 2.3%)
- Dabigatran	130	2.3% (1.9 - 2.6%)
- Edoxaban	32	2.0% (1.3 - 2.6%)
- Rivaroxaban	182	2.4% (2.0 - 2.7%)
**Discontinued use of any OAC**		
- Total	4,276	19.0% (18.5 - 19.5%)
- Apixaban	1,440	20.2% (19.3 - 21.1%)
- Dabigatran	842	14.4% (13.5 - 15.3%)
- Edoxaban	381	22.2% (20.2 - 24.2%)
- Rivaroxaban	1,613	20.7% (19.8 - 21.6%)
**Continued use of the initially collected DOAC**		
- Total	16,464	73.1% (72.5 - 73.7%)
- Apixaban	5,310	74.5% (73.4 - 75.5%)
- Dabigatran	4,233	72.4% (71.2 - 73.5%)
- Edoxaban	1,224	71.1% (68.9 - 73.2%)
- Rivaroxaban	5,697	72.9% (71.9 - 73.9%)

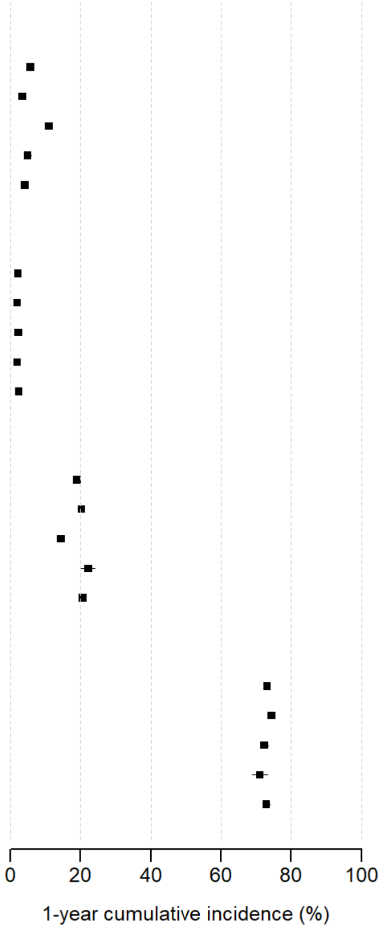

II. *65 up to 75 years of age*

This figure illustrates the one-year cumulative incidences of switching to a different OAC in the patients of 65 up to 75 years of age (*n*=22,513) in the longitudinal analysis cohort (*n*=66,090) stratified by initially collected DOAC type. The sum of all four treatment statuses is 100% for each subgroup (i.e., total, apixaban, dabigatran, edoxaban, rivaroxaban).

DOAC direct oral anticoagulant; *OAC* oral anticoagulant; *VKA* vitamin K antagonist.

Initially collected DOAC	No. of Patients	1-year cumulative incidence (95% confidence interval)
**Switched to a different DOAC**		
- Total	858	4.8% (4.5 - 5.1%)
- Apixaban	194	3.5% (3.0 - 4.0%)
- Dabigatran	368	8.6% (7.7 - 9.4%)
- Edoxaban	63	4.4% (3.3 - 5.4%)
- Rivaroxaban	233	3.6% (3.1 - 4.0%)
**Switched to a VKA**		
- Total	441	2.5% (2.2 - 2.7%)
- Apixaban	130	2.3% (2.0 - 2.7%)
- Dabigatran	126	3.0% (2.5 - 3.5%)
- Edoxaban	32	2.3% (1.5 - 3.0%)
- Rivaroxaban	153	2.4% (2.0 - 2.7%)
**Discontinued use of any OAC**		
- Total	6,824	38.0% (37.3 - 38.7%)
- Apixaban	2,227	39.7% (38.4 - 41.0%)
- Dabigatran	1,422	33.0% (31.6 - 34.4%)
- Edoxaban	612	41.3% (38.8 - 43.9%)
- Rivaroxaban	2,563	39.2% (38.0 - 40.4%)
**Continued use of the initially collected DOAC**		
- Total	9,826	54.7% (54.0 - 55.5%)
- Apixaban	3,063	54.6% (53.3 - 55.9%)
- Dabigatran	2,401	55.6% (54.1 - 57.1%)
- Edoxaban	774	52.3% (49.7 - 54.8%)
- Rivaroxaban	3,588	54.9% (53.7 - 56.1%)

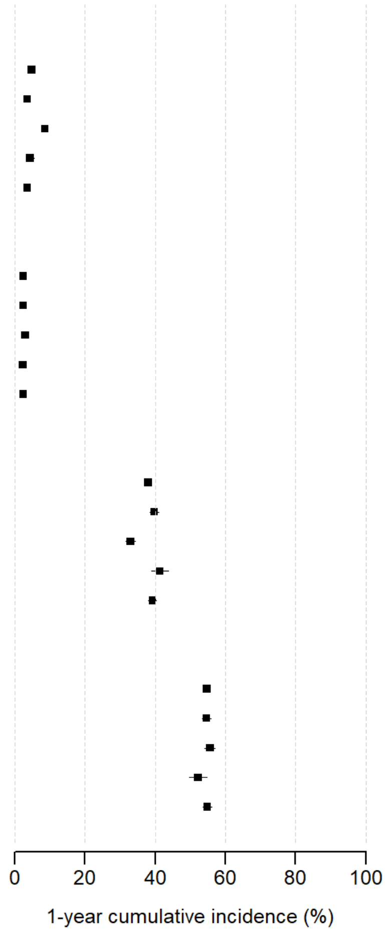

III. *Up to 65 years of age*

This figure illustrates the one-year cumulative incidences of switching to a different OAC in the patients of up to 65 years of age (*n*=17,949) in the longitudinal analysis cohort (*n*=66,090) stratified by initially collected DOAC type. The sum of all four treatment statuses is 100% for each subgroup (i.e., total, apixaban, dabigatran, edoxaban, rivaroxaban).

DOAC direct oral anticoagulant; *OAC* oral anticoagulant; *VKA* vitamin K antagonist.

B. Stratified by sex category

Initially collected DOAC	No. of Patients	1-year cumulative incidence (95% confidence interval)
**Switched to a different DOAC**		
- Total	1,657	4.5% (4.2 - 4.7%)
- Apixaban	346	2.9% (2.6 - 3.2%)
- Dabigatran	718	7.7% (7.2 - 8.3%)
- Edoxaban	127	4.4% (3.7 - 5.2%)
- Rivaroxaban	466	3.6% (3.3 - 3.9%)
**Switched to a VKA**		
- Total	799	2.2% (2.0 - 2.3%)
- Apixaban	227	1.9% (1.7 - 2.2%)
- Dabigatran	210	2.3% (2.0 - 2.6%)
- Edoxaban	52	1.8% (1.4 - 2.3%)
- Rivaroxaban	310	2.4% (2.1 - 2.7%)
**Discontinued use of any OAC**		
- Total	9,118	24.5% (24.1 - 24.9%)
- Apixaban	3,075	25.7% (24.9 - 26.5%)
- Dabigatran	1,820	19.5% (18.7 - 20.3%)
- Edoxaban	793	27.2% (25.6 - 28.8%)
- Rivaroxaban	3,430	26.4% (25.7 - 27.2%)
**Continued use of the initially collected DOAC**		
- Total	25,627	68.9% (68.4 - 69.4%)
- Apixaban	8,324	69.5% (68.7 - 70.3%)
- Dabigatran	6,578	70.5% (69.6 - 71.5%)
- Edoxaban	1,945	66.7% (64.9 - 68.4%)
- Rivaroxaban	8,780	67.6% (66.8 - 68.4%)

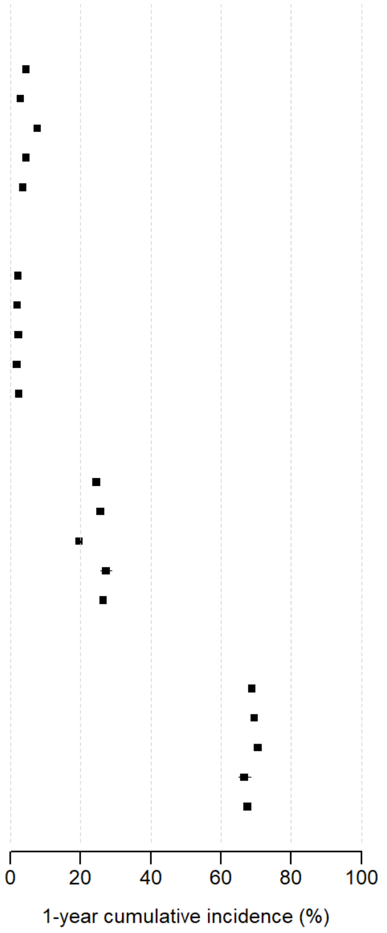

I. Male patients

This figure illustrates the one-year cumulative incidences of switching to a different OAC in the male patients (*n*=37,201) in the longitudinal analysis cohort (*n*=66,090) stratified by initially collected DOAC type. The sum of all four treatment statuses is 100% for each subgroup (i.e., total, apixaban, dabigatran, edoxaban, rivaroxaban).

DOAC direct oral anticoagulant; *OAC* oral anticoagulant; *VKA* vitamin K antagonist.

Initially collected DOAC	No. of Patients	1-year cumulative incidence (95% confidence interval)
**Switched to a different DOAC**		
- Total	1,400	4.9% (4.6 - 5.1%)
- Apixaban	296	3.0% (2.6 - 3.3%)
- Dabigatran	653	9.7% (9.0 - 10.4%)
- Edoxaban	93	4.5% (3.6 - 5.4%)
- Rivaroxaban	358	3.6% (3.2 - 3.9%)
**Switched to a VKA**		
- Total	590	2.0% (1.9 - 2.2%)
- Apixaban	173	1.8% (1.5 - 2.0%)
- Dabigatran	118	1.8% (1.5 - 2.1%)
- Edoxaban	46	2.3% (1.6 - 2.9%)
- Rivaroxaban	253	2.5% (2.2 - 2.8%)
**Discontinued use of any OAC**		
- Total	7,241	25.1% (24.6 - 25.6%)
- Apixaban	2,560	25.6% (24.8 - 26.5%)
- Dabigatran	1,132	16.8% (15.9 - 17.7%)
- Edoxaban	628	29.8% (27.8 - 31.7%)
- Rivaroxaban	2,921	29.1% (28.2 - 30.0%)
**Continued use of the initially collected DOAC**		
- Total	19,658	68.0% (67.5 - 68.6%)
- Apixaban	6,961	69.7% (68.8 - 70.6%)
- Dabigatran	4,837	71.8% (70.7 - 72.8%)
- Edoxaban	1,344	63.6% (61.6 - 65.7%)
- Rivaroxaban	6,516	64.8% (63.9 - 65.8%)

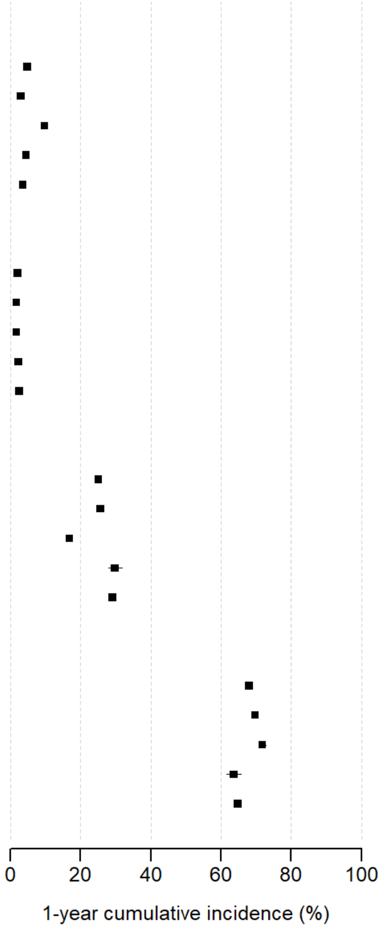

II. Female patients

This figure illustrates the one-year cumulative incidences of switching to a different OAC in the female patients (*n*=28,889) in the longitudinal analysis cohort (*n*=66,090) stratified by initially collected DOAC type. The sum of all four treatment statuses is 100% for each subgroup (i.e., total, apixaban, dabigatran, edoxaban, rivaroxaban).

DOAC direct oral anticoagulant; *OAC* oral anticoagulant; *VKA* vitamin K antagonist.

Initially collected DOAC	No. of Patients	1-year cumulative incidence (95% confidence interval)
**Switched to or continued on apixaban**		
- Total	16,265	24.6% (24.3 - 24.9%)
- Apixaban	15,285	69.6% (69.0 - 70.2%)
- Dabigatran	543	3.4% (3.1 - 3.7%)
- Edoxaban	78	1.6% (1.2 - 1.9%)
- Rivaroxaban	359	1.6% (1.4 - 1.7%)
**Switched to or continued on dabigatran**		
- Total	11,902	18.0% (17.7 - 18.3%)
- Apixaban	165	0.8% (0.6 - 0.9%)
- Dabigatran	11,415	71.0% (70.3 - 71.7%)
- Edoxaban	46	1.0% (0.7 - 1.2%)
- Rivaroxaban	276	1.2% (1.1 - 1.3%)
**Switched to or continued on edoxaban**		
- Total	3,868	5.9% (5.7 - 6.0%)
- Apixaban	182	0.8% (0.7 - 1.0%)
- Dabigatran	208	1.3% (1.1 - 1.5%)
- Edoxaban	3,289	65.4% (64.1 - 66.7%)
- Rivaroxaban	189	0.8% (0.7 - 0.9%)
**Switched to or continued on rivaroxaban**		
- Total	16,307	24.7% (24.3 - 25.0%)
- Apixaban	295	1.4% (1.2 - 1.5%)
- Dabigatran	620	3.9% (3.6 - 4.2%)
- Edoxaban	96	1.9% (1.6 - 2.3%)
- Rivaroxaban	15,296	66.4% (65.8 - 67.0%)
**Switched to a VKA**		
- Total	1,389	2.1% (2.0 - 2.2%)
- Apixaban	400	1.8% (1.7 - 2.0%)
- Dabigatran	328	2.1% (1.8 - 2.3%)
- Edoxaban	98	2.0% (1.6 - 2.4%)
- Rivaroxaban	563	2.5% (2.3 - 2.7%)
**Discontinued use of any OAC, but ongoing collection of other drugs**		
- Total	10,131	15.3% (15.1 - 15.6%)
- Apixaban	3,448	15.7% (15.2 - 16.2%)
- Dabigatran	1,870	11.6% (11.2 - 12.1%)
- Edoxaban	825	16.4% (15.4 - 17.5%)
- Rivaroxaban	3,988	17.3% (16.8 - 17.8%)
**Discontinued use of all prescriptions**		
- Total	6,228	9.4% (9.2 - 9.6%)
- Apixaban	2,187	10.0% (9.6 - 10.4%)
- Dabigatran	1,082	6.7% (6.4 - 7.1%)
- Edoxaban	596	11.9% (11.0 - 12.8%)
- Rivaroxaban	2,363	10.3% (9.9 - 10.7%)

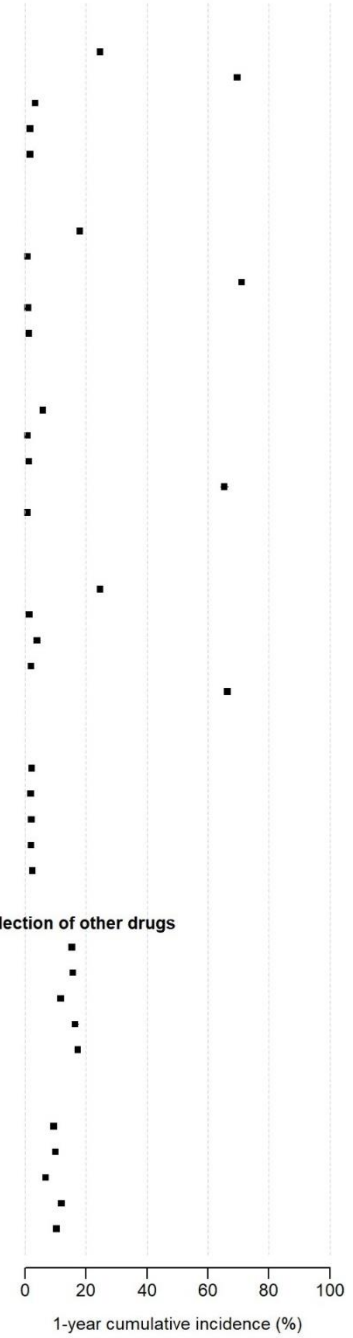

Figure S5. One-year cumulative incidences of switching among the individual direct oral anticoagulants subgroups

This figure illustrates the one-year cumulative incidences of switching to a different OAC in the longitudinal analysis cohort (*n*=66,090) stratified by initially collected DOAC type, but now providing data on switching to unique DOAC types. The sum of all four treatment statuses is 100% for each subgroup (i.e., total, apixaban, dabigatran, edoxaban, rivaroxaban).

DOAC direct oral anticoagulant; *OAC* oral anticoagulant; *VKA* vitamin K antagonist

Figure S6. Adjusted and unadjusted predictors of incident switching events within the first 12 months

Predictors	Switched to a different DOAC (*n* = 3,057)	All others (*n* = 63,033)	Multivariable Robust Poisson models Adjusted RR (95% CI)	Univariable Robust Poisson models Unadjusted RR (95% CI)
**Initially collected DOAC type**				
- Apixaban	642 (2.9%)	21,320 (97.1%)	1.00 (reference)	1.00 (reference)
- Dabigatran	1,371 (8.5%)	14,695 (91.5%)	3.33 (3.02 - 3.66)	2.92 (2.66 - 3.21)
- Edoxaban	220 (4.4%)	4,808 (95.6%)	1.56 (1.34 - 1.82)	1.50 (1.28 - 1.74)
- Rivaroxaban	824 (3.6%)	22,210 (96.4%)	1.25 (1.13 - 1.39)	1.22 (1.10 - 1.36)
**Initially collected DOAC dose**				
- Reduced dose	306 (2.6%)	11,642 (97.4%)	1.00 (reference)	1.00 (reference)
- Standard dose	2,751 (5.1%)	51,391 (94.9%)	2.54 (2.23 - 2.88)	1.98 (1.76 - 2.23)
**Sex category**				
- Male	1,657 (4.5%)	35,544 (95.5%)	1.00 (reference)	1.00 (reference)
- Female	1,400 (4.8%)	27,489 (95.2%)	1.22 (1.13 - 1.31)	1.09 (1.01 - 1.17)
**Age[a] — in years**				
- ≥18 and <55	287 (4.1%)	6,653 (95.9%)	1.00 (reference)	1.00 (reference)
- ≥55 and <70	1,144 (5.4%)	20,156 (94.6%)	1.21 (1.06 - 1.38)	1.30 (1.14 - 1.48)
- ≥70 and <85	1,456 (4.8%)	29,142 (95.2%)	1.20 (1.05 - 1.37)	1.15 (1.01 - 1.31)
- ≥85 and ≤106	170 (2.3%)	7,082 (97.7%)	0.82 (0.67 - 1.01)	0.57 (0.47 - 0.69)
**Clinical pattern of atrial fibrillation**				
- Persistent or paroxysmal	490 (5.1%)	9,164 (94.9%)	1.00 (reference)	1.00 (reference)
- Permanent	1,806 (4.8%)	35,992 (95.2%)	1.02 (0.92 - 1.13)	0.94 (0.85 - 1.04)
- Uncertain	761 (4.1%)	17,877 (95.9%)	0.92 (0.82 - 1.03)	0.80 (0.72 - 0.90)
**Atherosclerotic disease**				
- Absent	1,796 (4.5%)	38,368 (95.5%)	1.00 (reference)	1.00 (reference)
- Present	1,261 (4.9%)	24,665 (95.1%)	1.24 (1.15 - 1.34)	1.09 (1.01 - 1.17)
**Hypertension or heart failure**				
- Absent	1,447 (4.9%)	28,241 (95.1%)	1.00 (reference)	1.00 (reference)
- Present	1,610 (4.4%)	34,792 (95.6%)	0.94 (0.87 - 1.01)	0.91 (0.85 - 0.97)
**Diabetes mellitus**				
- Absent	2,650 (4.7%)	53,309 (95.3%)	1.00 (reference)	1.00 (reference)
- Present	407 (4.0%)	9,724 (96.0%)	0.87 (0.78 - 0.97)	0.85 (0.76 - 0.94)
**CHA_2DS_2VASc score**				
- 0	256 (4.6%)	5,255 (95.4%)	1.00 (reference)	1.00 (reference)
- 1	450 (4.6%)	9,285 (95.4%)	1.02 (0.87 - 1.18)	1.00 (0.85 - 1.16)
- 2	586 (5.0%)	11,155 (95.0%)	1.13 (0.98 - 1.31)	1.07 (0.93 - 1.24)
- 3	597 (4.9%)	11,642 (95.1%)	1.18 (1.02 - 1.37)	1.05 (0.91 - 1.22)
- 4	521 (4.6%)	10,756 (95.4%)	1.19 (1.02 - 1.38)	0.99 (0.86 - 1.16)
- 5	371 (4.3%)	8,336 (95.7%)	1.18 (1.00 - 1.38)	0.92 (0.78 - 1.08)
- ≥6	276 (4.0%)	6,604 (96.0%)	1.26 (1.06 - 1.50)	0.86 (0.73 - 1.02)
**Parkinson's disease**				
- Absent	3,013 (4.6%)	61,952 (95.4%)	1.00 (reference)	1.00 (reference)
- Present	44 (3.9%)	1,081 (96.1%)	0.89 (0.66 - 1.20)	0.84 (0.63 - 1.14)
**Mental disorders necessitating psycholeptics**				
- Absent	2,421 (4.5%)	50,832 (95.5%)	1.00 (reference)	1.00 (reference)
- Present	636 (5.0%)	12,201 (95.0%)	1.14 (1.04 - 1.25)	1.09 (1.00 - 1.19)
**Mental disorders necessitating psychoanaleptics**				
- Absent	2,746 (4.6%)	56,515 (95.4%)	1.00 (reference)	1.00 (reference)
- Present	311 (4.6%)	6,518 (95.4%)	0.96 (0.85 - 1.08)	0.98 (0.87 - 1.11)

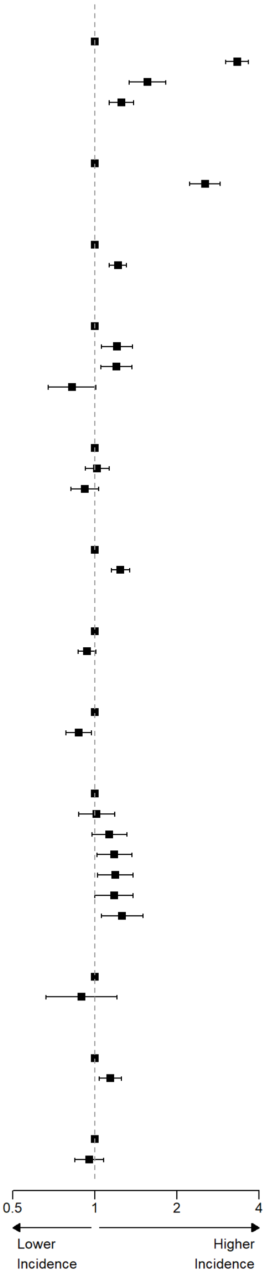

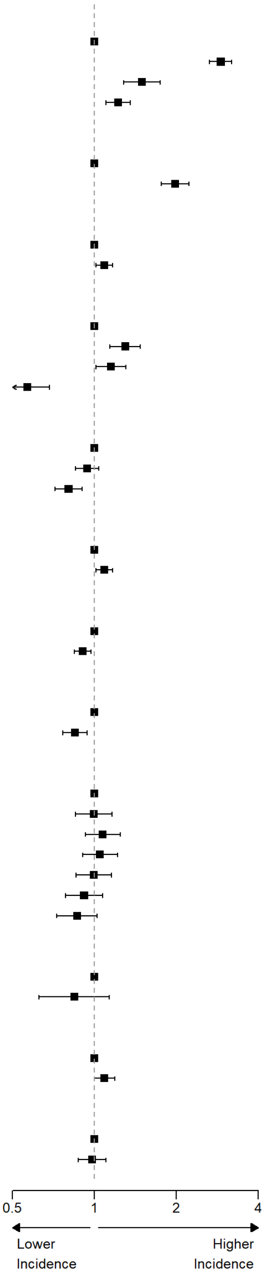

A. <u>Switching to a different direct oral anticoagulant</u>

This forest plot illustrates the adjusted and unadjusted associations between baseline characteristics and switching to a different DOAC within the first 12 months in the longitudinal analysis cohort (*n*=66,090). Baseline characteristics are presented in number of patients (% relative to stratum total), and the associations are in risk ratios.

CI confidence interval; *DOAC* direct oral anticoagulant; *RR* risk ratio.

[a] Categories were determined by the knot locations of the spline function with the optimal fit (i.e., 10th, 50th and 90th percentile) [20]. The results with age as a nonlinear term are presented in **Figure S7-S8**.

[b] Examples are sleeping, anxiety, and psychotic disorders

[c] Examples are depressive and bipolar disorders

Predictors	Switched to a VKA (*n* = 1,389)	All others (*n* = 64,701)	Multivariable Robust Poisson models Adjusted RR (95% CI)	Univariable Robust Poisson models Unadjusted RR (95% CI)
**Initially collected DOAC type**				
- Apixaban	400 (1.8%)	21,562 (98.2%)	1.00 (reference)	1.00 (reference)
- Dabigatran	328 (2.0%)	15,738 (98.0%)	1.19 (1.03 - 1.39)	1.12 (0.97 - 1.30)
- Edoxaban	98 (1.9%)	4,930 (98.1%)	1.09 (0.87 - 1.36)	1.07 (0.86 - 1.33)
- Rivaroxaban	563 (2.4%)	22,471 (97.6%)	1.36 (1.19 - 1.54)	1.34 (1.18 - 1.53)
**Initially collected DOAC dose**				
- Reduced dose	175 (1.5%)	11,773 (98.5%)	1.00 (reference)	1.00 (reference)
- Standard dose	1,214 (2.2%)	52,928 (97.8%)	1.49 (1.26 - 1.77)	1.53 (1.31 - 1.79)
**Sex category**				
- Male	799 (2.1%)	36,402 (97.9%)	1.00 (reference)	1.00 (reference)
- Female	590 (2.0%)	28,299 (98.0%)	0.99 (0.89 - 1.11)	0.95 (0.85 - 1.06)
**Age — in years**	70 [62 - 77]	71 [64 - 79]	0.99 (0.99 - 0.99)	0.99 (0.99 - 0.99)
**Clinical pattern of atrial fibrillation**				
- Persistent or paroxysmal	192 (2.0%)	9,462 (98.0%)	1.00 (reference)	1.00 (reference)
- Permanent	852 (2.3%)	36,946 (97.7%)	1.19 (1.01 - 1.39)	1.13 (0.97 - 1.33)
- Uncertain	345 (1.9%)	18,293 (98.1%)	0.98 (0.82 - 1.17)	0.93 (0.78 - 1.11)
**Atherosclerotic disease**				
- Absent	808 (2.0%)	39,356 (98.0%)	1.00 (reference)	1.00 (reference)
- Present	581 (2.2%)	25,345 (97.8%)	1.16 (1.03 - 1.30)	1.11 (1.00 - 1.24)
**Hypertension or heart failure**				
- Absent	579 (2.0%)	29,109 (98.0%)	1.00 (reference)	1.00 (reference)
- Present	810 (2.2%)	35,592 (97.8%)	1.18 (1.05 - 1.32)	1.14 (1.03 - 1.27)
**Diabetes mellitus**				
- Absent	1,179 (2.1%)	54,780 (97.9%)	1.00 (reference)	1.00 (reference)
- Present	210 (2.1%)	9,921 (97.9%)	0.93 (0.80 - 1.08)	0.98 (0.85 - 1.14)
**CHA_2DS_2VASc score**				
- 0	119 (2.2%)	5,392 (97.8%)	1.00 (reference)	1.00 (reference)
- 1	221 (2.3%)	9,514 (97.7%)	1.05 (0.84 - 1.31)	1.05 (0.84 - 1.31)
- 2	238 (2.0%)	11,503 (98.0%)	0.94 (0.75 - 1.17)	0.94 (0.75 - 1.17)
- 3	264 (2.2%)	11,975 (97.8%)	1.02 (0.82 - 1.26)	1.00 (0.80 - 1.24)
- 4	216 (1.9%)	11,061 (98.1%)	0.92 (0.73 - 1.15)	0.89 (0.71 - 1.11)
- 5	184 (2.1%)	8,523 (97.9%)	1.04 (0.82 - 1.31)	0.98 (0.78 - 1.23)
- ≥6	147 (2.1%)	6,733 (97.9%)	1.09 (0.84 - 1.40)	0.99 (0.78 - 1.26
**Parkinson's disease**				
- Absent	1,365 (2.1%)	63,600 (97.9%)	1.00 (reference)	1.00 (reference)
- Present	24 (2.1%)	1,101 (97.9%)	1.01 (0.67 - 1.51)	1.02 (0.68 - 1.52)
**Mental disorders necessitating psycholeptics**				
- Absent	1,102 (2.1%)	52,151 (97.9%)	1.00 (reference)	1.00 (reference)
- Present	287 (2.2%)	12,550 (97.8%)	1.07 (0.93 - 1.22)	1.08 (0.95 - 1.23)
**Mental disorders necessitating psychoanaleptics**				
- Absent	1,231 (2.1%)	58,030 (97.9%)	1.00 (reference)	1.00 (reference)
- Present	158 (2.3%)	6,671 (97.7%)	1.06 (0.89 - 1.26)	1.11 (0.94 - 1.31)

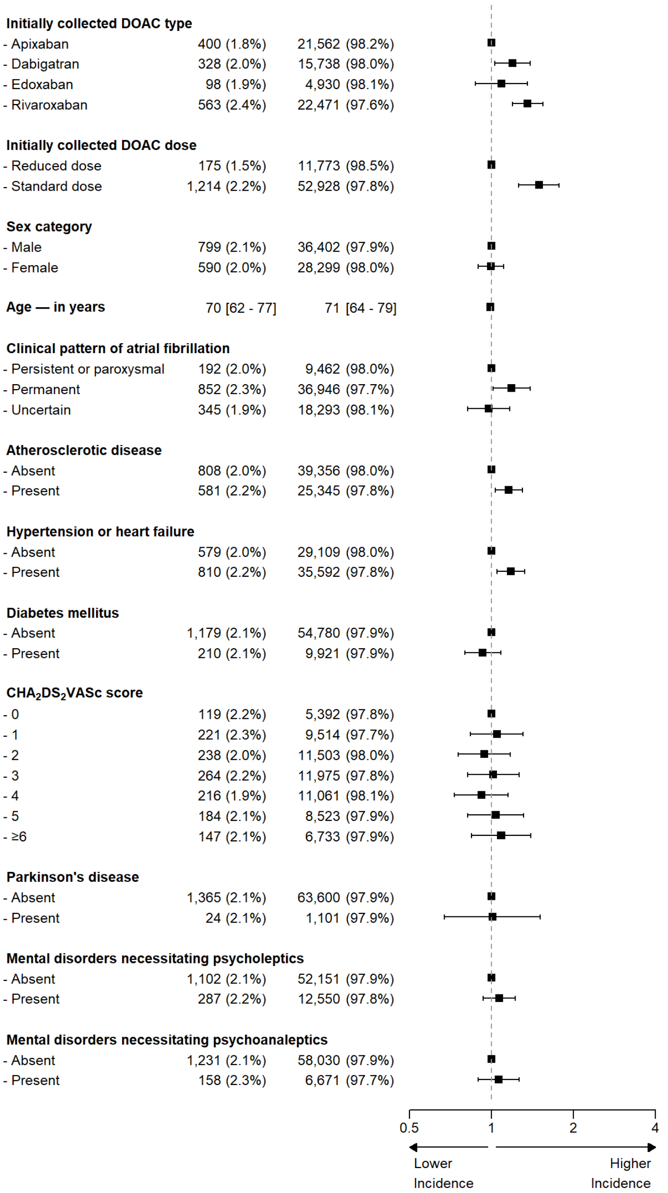

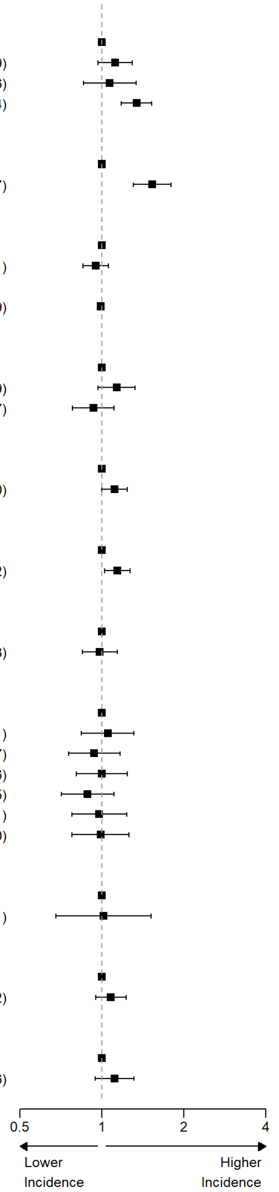

B. <u>Switching to a vitamin K antagonist</u>

This forest plot illustrates the adjusted and unadjusted associations between baseline characteristics and switching to a VKA within the first 12 months in the longitudinal analysis cohort (*n*=66,090). Baseline characteristics are presented in number of patients (% relative to stratum total), and the associations are in risk ratios.

CI confidence interval; *DOAC* direct oral anticoagulant; *RR* risk ratio; *VKA* vitamin K antagonist.

[a] The relationship between age and incident switching to a different DOAC was best fitted by linear term in both the univariable and multivariable model.

[b] Examples are sleeping, anxiety, and psychotic disorders

[c] Examples are depressive and bipolar disorders

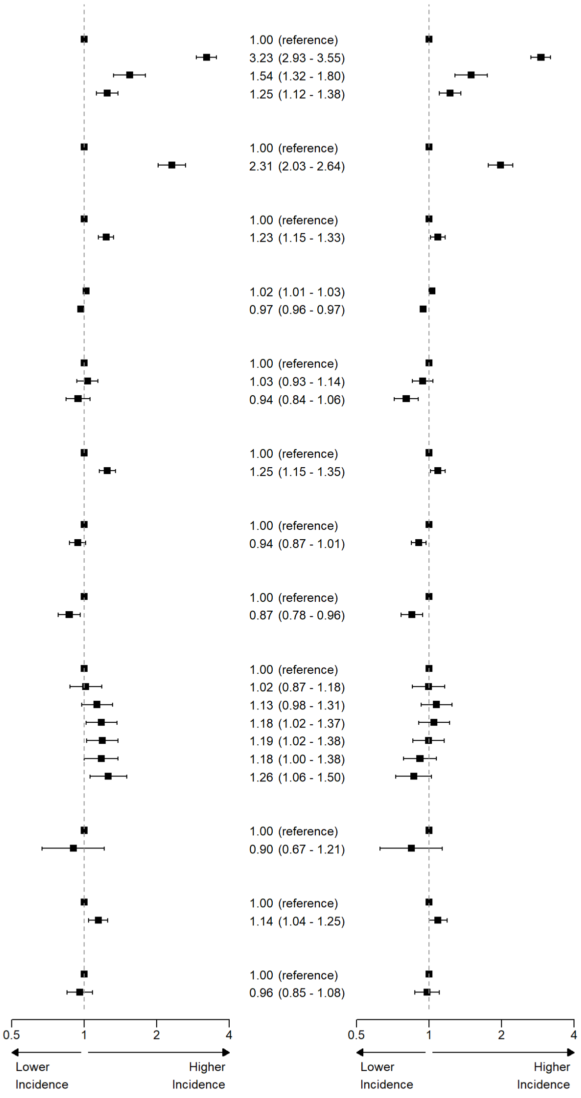

Predictors	Switched to a different DOAC (*n* = 3,057)	All others (*n* = 63,033)	Multivariable Robust Poisson models Adjusted RR (95% CI)	Univariable Robust Poisson models Unadjusted RR (95% CI)
**Initially collected DOAC type**				
- Apixaban	642 (2.9%)	21,320 (97.1%)	1.00 (reference)	1.00 (reference)
- Dabigatran	1,371 (8.5%)	14,695 (91.5%)	3.23 (2.93 - 3.55)	2.92 (2.66 - 3.21)
- Edoxaban	220 (4.4%)	4,808 (95.6%)	1.54 (1.32 - 1.80)	1.50 (1.28 - 1.74)
- Rivaroxaban	824 (3.6%)	22,210 (96.4%)	1.25 (1.12 - 1.38)	1.22 (1.10 - 1.36)
**Initially collected DOAC dose**				
- Reduced dose	306 (2.6%)	11,642 (97.4%)	1.00 (reference)	1.00 (reference)
- Standard dose	2,751 (5.1%)	51,391 (94.9%)	2.31 (2.03 - 2.64)	1.98 (1.76 - 2.23)
**Sex category**				
- Male	1,657 (4.5%)	35,544 (95.5%)	1.00 (reference)	1.00 (reference)
- Female	1,400 (4.8%)	27,489 (95.2%)	1.23 (1.15 - 1.33)	1.09 (1.01 - 1.17)
**Age[a] — in years**	70 [64 - 76]	71 [64 - 79]		
- First coefficient			1.02 (1.01 - 1.03)	1.03 (1.02 - 1.03)
- Second coefficient			0.97 (0.96 - 0.97)	0.95 (0.94 - 0.95)
**Clinical pattern of atrial fibrillation**				
- Persistent or paroxysmal	490 (5.1%)	9,164 (94.9%)	1.00 (reference)	1.00 (reference)
- Permanent	1,806 (4.8%)	35,992 (95.2%)	1.03 (0.93 - 1.14)	0.94 (0.85 - 1.04)
- Uncertain	761 (4.1%)	17,877 (95.9%)	0.94 (0.84 - 1.06)	0.80 (0.72 - 0.90)
**Atherosclerotic disease**				
- Absent	1,796 (4.5%)	38,368 (95.5%)	1.00 (reference)	1.00 (reference)
- Present	1,261 (4.9%)	24,665 (95.1%)	1.25 (1.15 - 1.35)	1.09 (1.01 - 1.17)
**Hypertension or heart failure**				
- Absent	1,447 (4.9%)	28,241 (95.1%)	1.00 (reference)	1.00 (reference)
- Present	1,610 (4.4%)	34,792 (95.6%)	0.94 (0.87 - 1.01)	0.91 (0.85 - 0.97)
**Diabetes mellitus**				
- Absent	2,650 (4.7%)	53,309 (95.3%)	1.00 (reference)	1.00 (reference)
- Present	407 (4.0%)	9,724 (96.0%)	0.87 (0.78 - 0.96)	0.85 (0.76 - 0.94)
**CHA_2DS_2VASc score**				
- 0	256 (4.6%)	5,255 (95.4%)	1.00 (reference)	1.00 (reference)
- 1	450 (4.6%)	9,285 (95.4%)	1.02 (0.87 - 1.18)	1.00 (0.85 - 1.16)
- 2	586 (5.0%)	11,155 (95.0%)	1.13 (0.98 - 1.31)	1.07 (0.93 - 1.24)
- 3	597 (4.9%)	11,642 (95.1%)	1.18 (1.02 - 1.37)	1.05 (0.91 - 1.22)
- 4	521 (4.6%)	10,756 (95.4%)	1.19 (1.02 - 1.38)	0.99 (0.86 - 1.16)
- 5	371 (4.3%)	8,336 (95.7%)	1.18 (1.00 - 1.38)	0.92 (0.78 - 1.08)
- ≥6	276 (4.0%)	6,604 (96.0%)	1.26 (1.06 - 1.50)	0.86 (0.73 - 1.02)
**Parkinson's disease**				
- Absent	3,013 (4.6%)	61,952 (95.4%)	1.00 (reference)	1.00 (reference)
- Present	44 (3.9%)	1,081 (96.1%)	0.90 (0.67 - 1.21)	0.84 (0.63 - 1.14)
**Mental disorders necessitating psycholeptics**				
- Absent	2,421 (4.5%)	50,832 (95.5%)	1.00 (reference)	1.00 (reference)
- Present	636 (5.0%)	12,201 (95.0%)	1.14 (1.04 - 1.25)	1.09 (1.00 - 1.19)
**Mental disorders necessitating psychoanaleptics**				
- Absent	2,746 (4.6%)	56,515 (95.4%)	1.00 (reference)	1.00 (reference)
- Present	311 (4.6%)	6,518 (95.4%)	0.96 (0.85 - 1.08)	0.98 (0.87 - 1.11)

Figure S7. Predictors of switching to a different direct oral anticoagulant within the first 12 months with age fitted with a restricted cubic spline function

This forest plot illustrates the adjusted and unadjusted associations between baseline characteristics and switching to a different DOAC within the first 12 months in the longitudinal analysis cohort (*n*=66,090), but with age fitted as a ratio variable. Baseline characteristics are presented in number of patients (% relative to stratum total), and the associations are in risk ratios.

CI confidence interval; *DOAC* direct oral anticoagulant; *RR* risk ratio.

[a] Knot locations were the 10th, 50th and 90th percentile of age [20]. The nonlinear relationship is presented in **Figure S8**.

[b] Examples are sleeping, anxiety, and psychotic disorders

[c] Examples are depressive and bipolar disorders

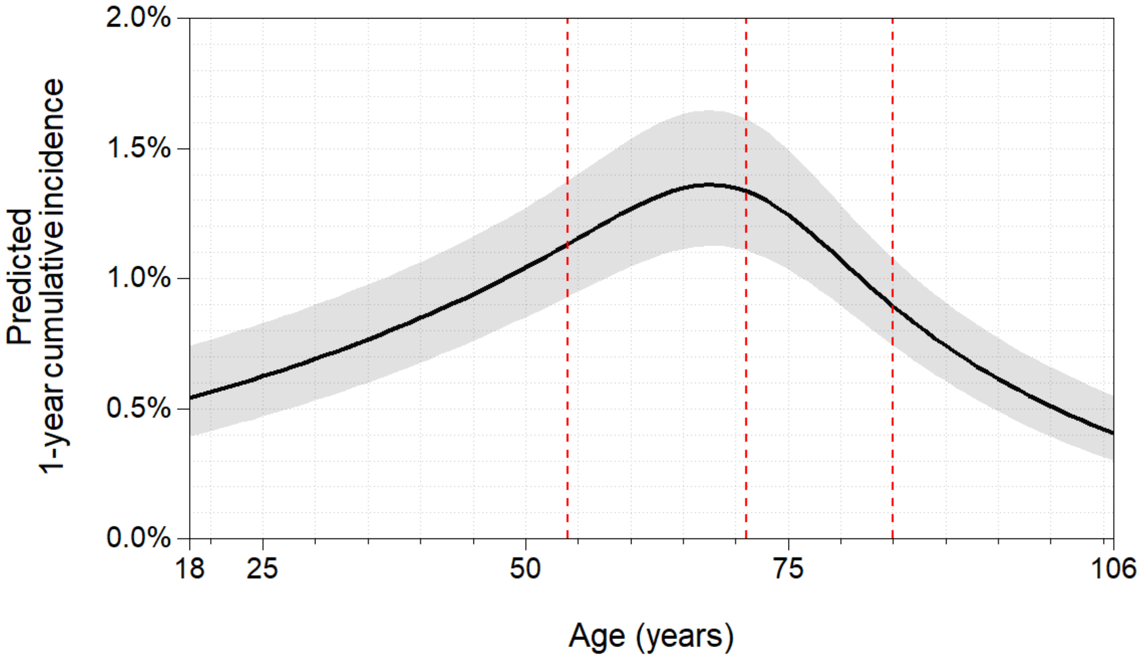

Figure S8. The adjusted nonlinear relationship between age and switching to a different direct oral anticoagulant as fitted with a restricted cubic spline function

This figure illustrates the predicted one-year cumulative incidence of switching to a different direct oral anticoagulant for different values of age. The dashed red lines indicate the knot locations of the restricted cubic spline function (i.e., 10th, 50th and 90th percentiles).

Figure S9. Adjusted and unadjusted predictors of incident switching events within the first 12 months, stratified by initially collected DOAC type

A. Switching to a different direct oral anticoagulant

Predictors	Switched to a different DOAC (*n* = 642)	All others (*n* = 21,320)	Multivariable Robust Poisson models Adjusted RR (95% CI)	Univariable Robust Poisson models Unadjusted RR (95% CI)
**Initially collected DOAC dose**				
- Reduced dose	21 (0.8%)	2,554 (99.2%)	1.00 (reference)	1.00 (reference)
- Standard dose	621 (3.2%)	18,766 (96.8%)	3.61 (2.32 - 5.61)	3.93 (2.55 - 6.06)
**Sex category**				
- Male	346 (2.9%)	11,626 (97.1%)	1.00 (reference)	1.00 (reference)
- Female	296 (3.0%)	9,694 (97.0%)	1.10 (0.94 - 1.28)	1.03 (0.88 - 1.19)
**Age[a] — in years**	70 [63 - 76]	72 [64 - 80]	0.99 (0.99 - 1.00)	0.99 (0.98 - 0.99)
**Clinical pattern of atrial fibrillation**				
- Persistent or paroxysmal	106 (3.3%)	3,060 (96.7%)	1.00 (reference)	1.00 (reference)
- Permanent	354 (2.8%)	12,234 (97.2%)	0.93 (0.75 - 1.15)	0.84 (0.68 - 1.04)
- Uncertain	182 (2.9%)	6,026 (97.1%)	0.88 (0.69 - 1.12)	0.88 (0.69 - 1.11)
**Atherosclerotic disease**				
- Absent	393 (3.0%)	12,662 (97.0%)	1.00 (reference)	1.00 (reference)
- Present	249 (2.8%)	8,658 (97.2%)	1.10 (0.93 - 1.31)	0.93 (0.79 - 1.09)
**Hypertension or heart failure**				
- Absent	312 (3.3%)	9,113 (96.7%)	1.00 (reference)	1.00 (reference)
- Present	330 (2.6%)	12,207 (97.4%)	0.88 (0.75 - 1.04)	0.80 (0.68 - 0.93)
**Diabetes mellitus**				
- Absent	565 (3.1%)	17,844 (96.9%)	1.00 (reference)	1.00 (reference)
- Present	77 (2.2%)	3,476 (97.8%)	0.74 (0.59 - 0.95)	0.71 (0.56 - 0.89)
**CHA_2DS_2VASc score**				
- 0	72 (4.2%)	1,626 (95.8%)	1.00 (reference)	1.00 (reference)
- 1	101 (3.4%)	2,899 (96.6%)	0.80 (0.60 - 1.08)	0.79 (0.59 - 1.07)
- 2	101 (2.8%)	3,547 (97.2%)	0.67 (0.50 - 0.90)	0.65 (0.49 - 0.88)
- 3	127 (3.1%)	3,933 (96.9%)	0.78 (0.58 - 1.04)	0.74 (0.56 - 0.98)
- 4	103 (2.7%)	3,745 (97.3%)	0.70 (0.52 - 0.94)	0.63 (0.47 - 0.85)
- 5	77 (2.4%)	3,068 (97.6%)	0.65 (0.47 - 0.91)	0.58 (0.42 - 0.79)
- ≥6	61 (2.4%)	2,502 (97.6%)	0.70 (0.49 - 0.98)	0.56 (0.40 - 0.79)
**Parkinson's disease**				
- Absent	633 (2.9%)	20,953 (97.1%)	1.00 (reference)	1.00 (reference)
- Present	9 (2.4%)	367 (97.6%)	0.88 (0.46 - 1.70)	0.82 (0.42 - 1.58)
**Mental disorders necessitating psycholeptics[b]**				
- Absent	509 (2.9%)	17,095 (97.1%)	1.00 (reference)	1.00 (reference)
- Present	133 (3.1%)	4,225 (96.9%)	1.11 (0.91 - 1.36)	1.06 (0.87 - 1.27)
**Mental disorders necessitating psychoanaleptics[c]**				
- Absent	577 (2.9%)	19,070 (97.1%)	1.00 (reference)	1.00 (reference)
- Present	65 (2.8%)	2,250 (97.2%)	0.94 (0.72 - 1.23)	0.96 (0.74 - 1.23)

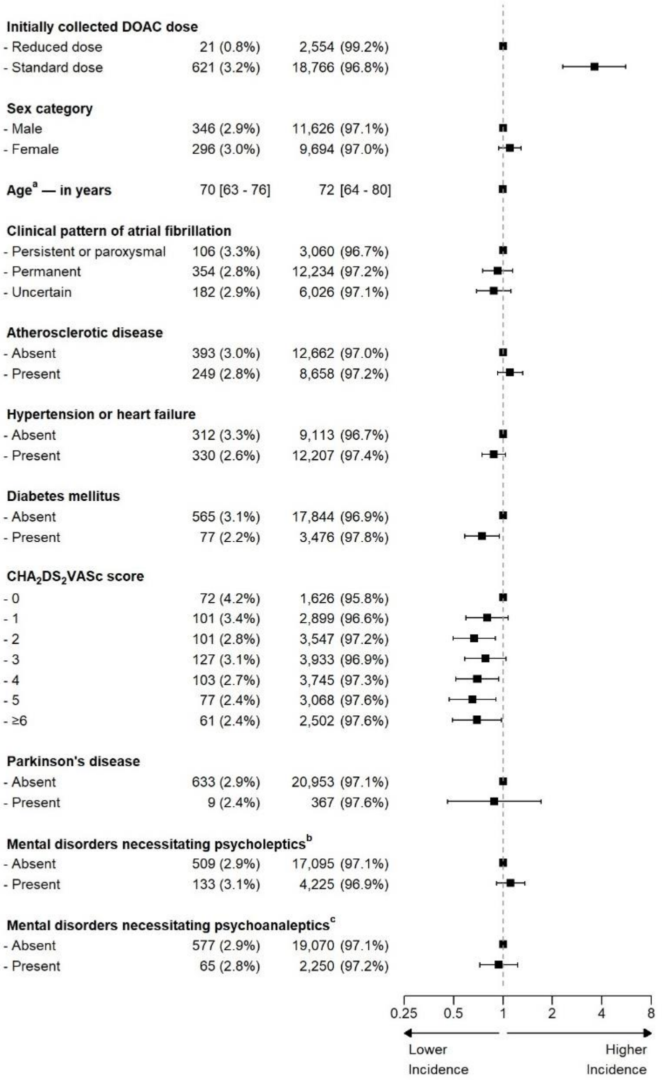

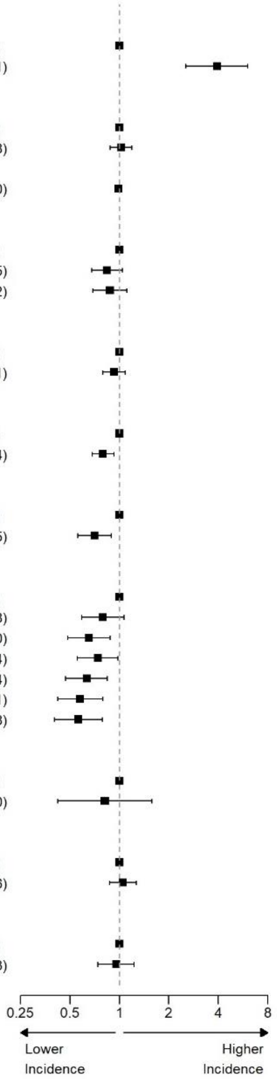

I. Apixaban

This forest plot illustrates the adjusted and unadjusted associations between baseline characteristics and switching to a different DOAC within the first 12 months for the patients who initially collected apixaban in the longitudinal analysis cohort (*n*=21,962). Baseline characteristics are presented in number of patients (% relative to stratum total), and the associations are in risk ratios.

CI confidence interval; *DOAC* direct oral anticoagulant; *RR* risk ratio.

[a] The relationship between age and incident switching was non-linear in the univariable model but linear in the multivariable model. We decided to report the results of both models using a linear fit; [b] Examples are sleeping, anxiety, and psychotic disorders; [c] Examples are depressive and bipolar disorders

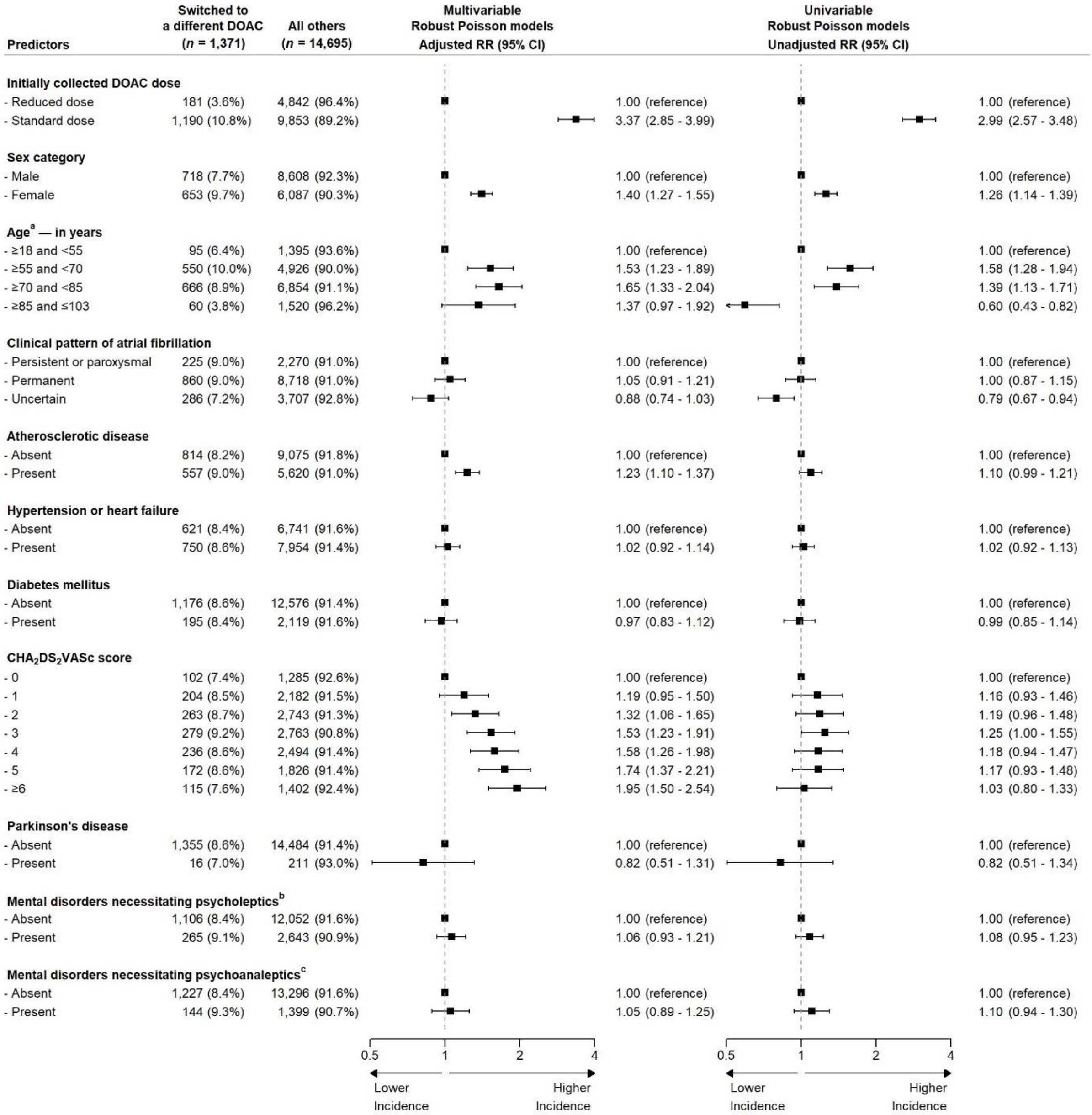

II. Dabigatran

This forest plot illustrates the adjusted and unadjusted associations between baseline characteristics and switching to a different DOAC within the first 12 months for the patients who initially collected dabigatran in the longitudinal analysis cohort (*n*=16,066). Baseline characteristics are presented in number of patients (% relative to stratum total), and the associations are in risk ratios.

CI confidence interval; *DOAC* direct oral anticoagulant; *RR* risk ratio.

[a] Categories were determined by the knot locations of the spline function with the optimal fit (i.e., 10th, 50th and 90th percentile) [20]; [b] Examples are sleeping, anxiety, and psychotic disorders; [c] Examples are depressive and bipolar disorders

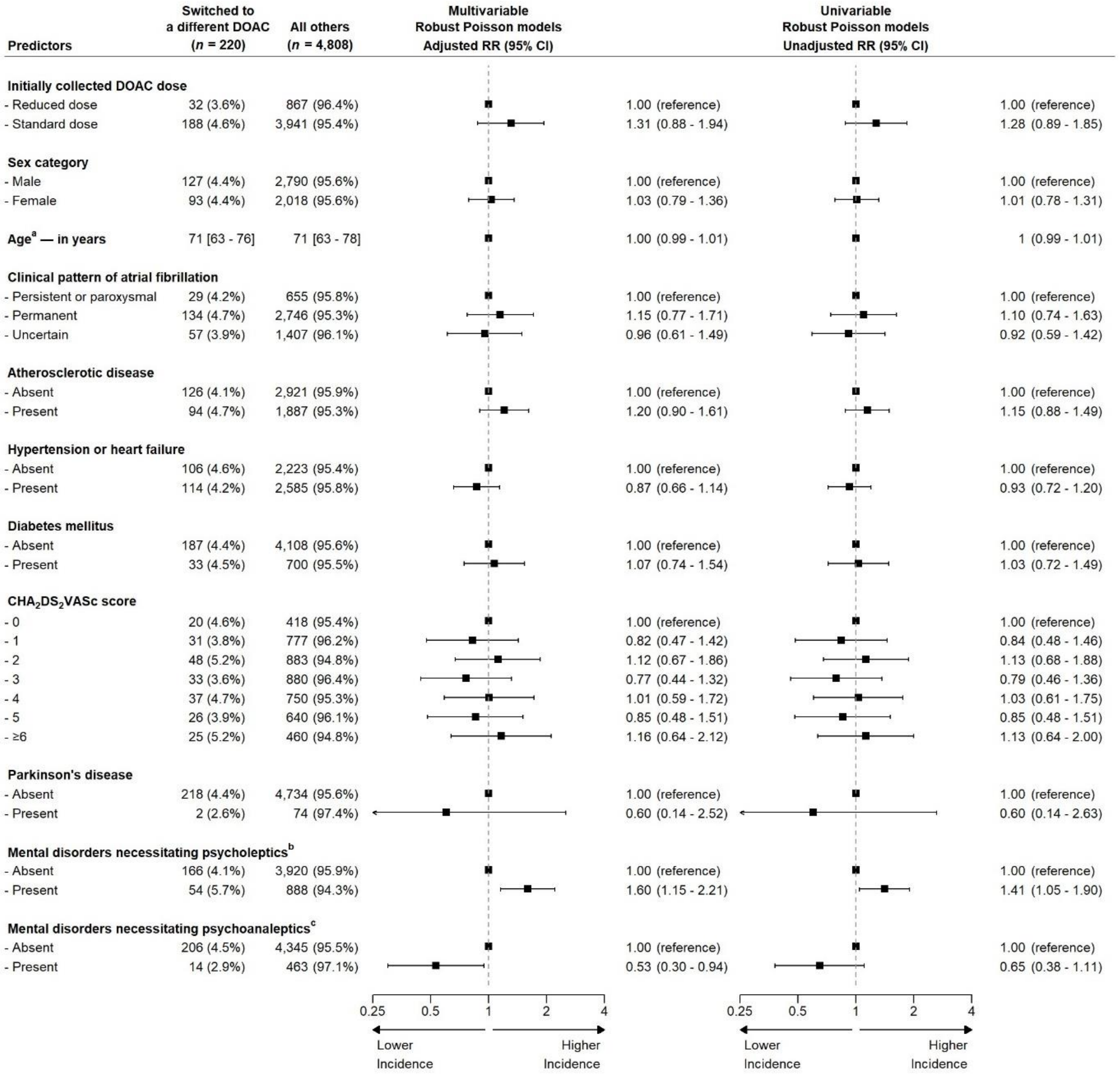

Predictors	Switched to a different DOAC (*n* = 220)	All others (*n* = 4,808)	Multivariable Robust Poisson models Adjusted RR (95% CI)	Univariable Robust Poisson models Unadjusted RR (95% CI)
**Initially collected DOAC dose**				
- Reduced dose	32 (3.6%)	867 (96.4%)	1.00 (reference)	1.00 (reference)
- Standard dose	188 (4.6%)	3,941 (95.4%)	1.31 (0.88 - 1.94)	1.28 (0.89 - 1.85)
**Sex category**				
- Male	127 (4.4%)	2,790 (95.6%)	1.00 (reference)	1.00 (reference)
- Female	93 (4.4%)	2,018 (95.6%)	1.03 (0.79 - 1.36)	1.01 (0.78 - 1.31)
**Age[a] — in years**	71 [63 - 76]	71 [63 - 78]	1.00 (0.99 - 1.01)	1 (0.99 - 1.01)
**Clinical pattern of atrial fibrillation**				
- Persistent or paroxysmal	29 (4.2%)	655 (95.8%)	1.00 (reference)	1.00 (reference)
- Permanent	134 (4.7%)	2,746 (95.3%)	1.15 (0.77 - 1.71)	1.10 (0.74 - 1.63)
- Uncertain	57 (3.9%)	1,407 (96.1%)	0.96 (0.61 - 1.49)	0.92 (0.59 - 1.42)
**Atherosclerotic disease**				
- Absent	126 (4.1%)	2,921 (95.9%)	1.00 (reference)	1.00 (reference)
- Present	94 (4.7%)	1,887 (95.3%)	1.20 (0.90 - 1.61)	1.15 (0.88 - 1.49)
**Hypertension or heart failure**				
- Absent	106 (4.6%)	2,223 (95.4%)	1.00 (reference)	1.00 (reference)
- Present	114 (4.2%)	2,585 (95.8%)	0.87 (0.66 - 1.14)	0.93 (0.72 - 1.20)
**Diabetes mellitus**				
- Absent	187 (4.4%)	4,108 (95.6%)	1.00 (reference)	1.00 (reference)
- Present	33 (4.5%)	700 (95.5%)	1.07 (0.74 - 1.54)	1.03 (0.72 - 1.49)
**CHA_2DS_2VASc score**				
- 0	20 (4.6%)	418 (95.4%)	1.00 (reference)	1.00 (reference)
- 1	31 (3.8%)	777 (96.2%)	0.82 (0.47 - 1.42)	0.84 (0.48 - 1.46)
- 2	48 (5.2%)	883 (94.8%)	1.12 (0.67 - 1.86)	1.13 (0.68 - 1.88)
- 3	33 (3.6%)	880 (96.4%)	0.77 (0.44 - 1.32)	0.79 (0.46 - 1.36)
- 4	37 (4.7%)	750 (95.3%)	1.01 (0.59 - 1.72)	1.03 (0.61 - 1.75)
- 5	26 (3.9%)	640 (96.1%)	0.85 (0.48 - 1.51)	0.85 (0.48 - 1.51)
- ≥6	25 (5.2%)	460 (94.8%)	1.16 (0.64 - 2.12)	1.13 (0.64 - 2.00)
**Parkinson's disease**				
- Absent	218 (4.4%)	4,734 (95.6%)	1.00 (reference)	1.00 (reference)
- Present	2 (2.6%)	74 (97.4%)	0.60 (0.14 - 2.52)	0.60 (0.14 - 2.63)
**Mental disorders necessitating psycholeptics[b]**				
- Absent	166 (4.1%)	3,920 (95.9%)	1.00 (reference)	1.00 (reference)
- Present	54 (5.7%)	888 (94.3%)	1.60 (1.15 - 2.21)	1.41 (1.05 - 1.90)
**Mental disorders necessitating psychoanaleptics[c]**				
- Absent	206 (4.5%)	4,345 (95.5%)	1.00 (reference)	1.00 (reference)
- Present	14 (2.9%)	463 (97.1%)	0.53 (0.30 - 0.94)	0.65 (0.38 - 1.11)

III. Edoxaban

This forest plot illustrates the adjusted and unadjusted associations between baseline characteristics and switching to a different DOAC within the first 12 months for the patients who initially collected edoxaban in the longitudinal analysis cohort (*n*=5,028). Baseline characteristics are presented in number of patients (% relative to stratum total), and the associations are in risk ratios.

CI confidence interval; *DOAC* direct oral anticoagulant; *RR* risk ratio.

[a] The relationship between age and incident switching was best fitted by a linear function in both the univariable and multivariable model; [b] Examples are sleeping, anxiety, and psychotic disorders; [c] Examples are depressive and bipolar disorders

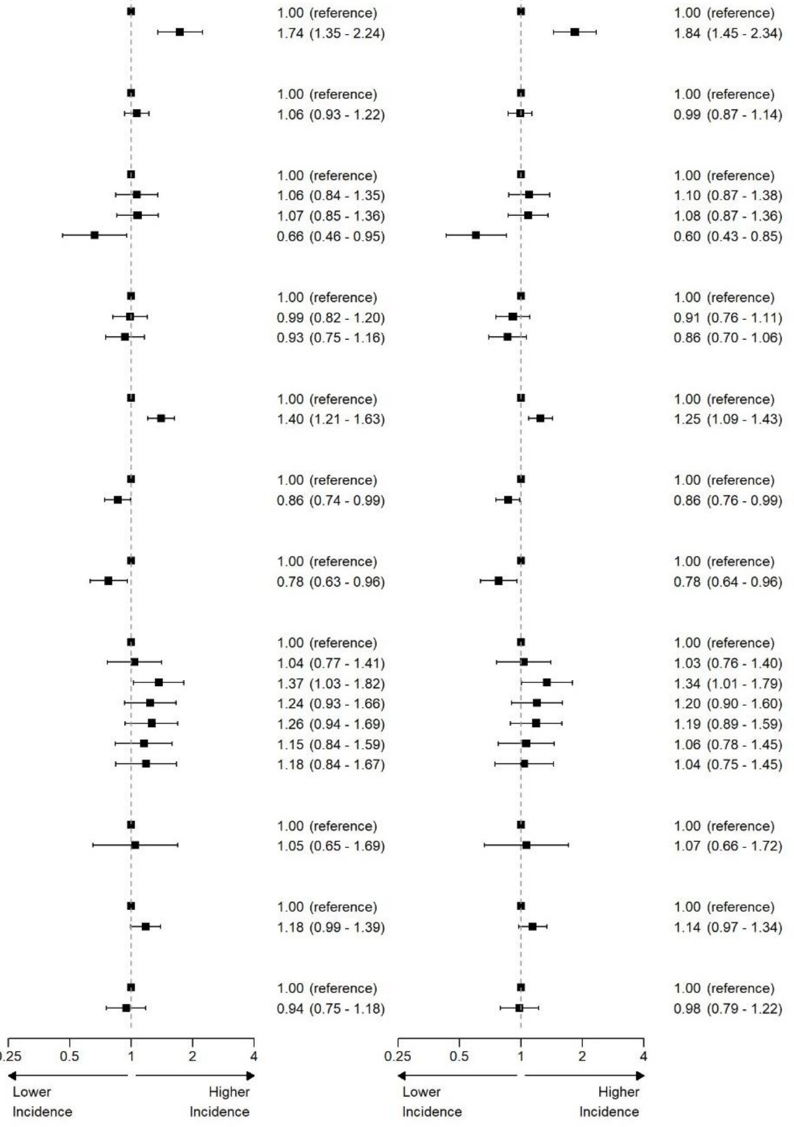

Predictors	Switched to a different DOAC (*n* = 824)	All others (*n* = 22,210)	Multivariable Robust Poisson models Adjusted RR (95% CI)	Univariable Robust Poisson models Unadjusted RR (95% CI)
**Initially collected DOAC dose**				
- Reduced dose	72 (2.1%)	3,379 (97.9%)	1.00 (reference)	1.00 (reference)
- Standard dose	752 (3.8%)	18,831 (96.2%)	1.74 (1.35 - 2.24)	1.84 (1.45 - 2.34)
**Sex category**				
- Male	466 (3.6%)	12,520 (96.4%)	1.00 (reference)	1.00 (reference)
- Female	358 (3.6%)	9,690 (96.4%)	1.06 (0.93 - 1.22)	0.99 (0.87 - 1.14)
**Age[a] — in years**				
- ≥18 and <55	91 (3.5%)	2,528 (96.5%)	1.00 (reference)	1.00 (reference)
- ≥55 and <70	287 (3.8%)	7,239 (96.2%)	1.06 (0.84 - 1.35)	1.10 (0.87 - 1.38)
- ≥70 and <85	396 (3.8%)	10,113 (96.2%)	1.07 (0.85 - 1.36)	1.08 (0.87 - 1.36)
- ≥85 and ≤106	50 (2.1%)	2,330 (97.9%)	0.66 (0.46 - 0.95)	0.60 (0.43 - 0.85)
**Clinical pattern of atrial fibrillation**				
- Persistent or paroxysmal	130 (3.9%)	3,179 (96.1%)	1.00 (reference)	1.00 (reference)
- Permanent	458 (3.6%)	12,294 (96.4%)	0.99 (0.82 - 1.20)	0.91 (0.76 - 1.11)
- Uncertain	236 (3.4%)	6,737 (96.6%)	0.93 (0.75 - 1.16)	0.86 (0.70 - 1.06)
**Atherosclerotic disease**				
- Absent	463 (3.3%)	13,710 (96.7%)	1.00 (reference)	1.00 (reference)
- Present	361 (4.1%)	8,500 (95.9%)	1.40 (1.21 - 1.63)	1.25 (1.09 - 1.43)
**Hypertension or heart failure**				
- Absent	408 (3.9%)	10,164 (96.1%)	1.00 (reference)	1.00 (reference)
- Present	416 (3.3%)	12,046 (96.7%)	0.86 (0.74 - 0.99)	0.86 (0.76 - 0.99)
**Diabetes mellitus**				
- Absent	722 (3.7%)	18,781 (96.3%)	1.00 (reference)	1.00 (reference)
- Present	102 (2.9%)	3,429 (97.1%)	0.78 (0.63 - 0.96)	0.78 (0.64 - 0.96)
**CHA_2DS_2VASc score**				
- 0	62 (3.1%)	1,926 (96.9%)	1.00 (reference)	1.00 (reference)
- 1	114 (3.2%)	3,427 (96.8%)	1.04 (0.77 - 1.41)	1.03 (0.76 - 1.40)
- 2	174 (4.2%)	3,982 (95.8%)	1.37 (1.03 - 1.82)	1.34 (1.01 - 1.79)
- 3	158 (3.7%)	4,066 (96.3%)	1.24 (0.93 - 1.66)	1.20 (0.90 - 1.60)
- 4	145 (3.7%)	3,767 (96.3%)	1.26 (0.94 - 1.69)	1.19 (0.89 - 1.59)
- 5	96 (3.3%)	2,802 (96.7%)	1.15 (0.84 - 1.59)	1.06 (0.78 - 1.45)
- ≥6	75 (3.2%)	2,240 (96.8%)	1.18 (0.84 - 1.67)	1.04 (0.75 - 1.45)
**Parkinson's disease**				
- Absent	807 (3.6%)	21,781 (96.4%)	1.00 (reference)	1.00 (reference)
- Present	17 (3.8%)	429 (96.2%)	1.05 (0.65 - 1.69)	1.07 (0.66 - 1.72)
**Mental disorders necessitating psycholeptics[b]**				
- Absent	640 (3.5%)	17,765 (96.5%)	1.00 (reference)	1.00 (reference)
- Present	184 (4.0%)	4,445 (96.0%)	1.18 (0.99 - 1.39)	1.14 (0.97 - 1.34)
**Mental disorders necessitating psychoanaleptics[c]**				
- Absent	736 (3.6%)	19,804 (96.4%)	1.00 (reference)	1.00 (reference)
- Present	88 (3.5%)	2,406 (96.5%)	0.94 (0.75 - 1.18)	0.98 (0.79 - 1.22)

IV. Rivaroxaban

This forest plot illustrates the adjusted and unadjusted associations between baseline characteristics and switching to a different DOAC within the first 12 months for the patients who initially collected rivaroxaban in the longitudinal analysis cohort (*n*=23,034). Baseline characteristics are presented in number of patients (% relative to stratum total), and the associations are in risk ratios.

CI confidence interval; *DOAC* direct oral anticoagulant; *RR* risk ratio.

[a] Categories were determined by the knot locations of the spline function with the optimal fit (i.e., 10th, 50th and 90th percentile);[20] [b] Examples are sleeping, anxiety, and psychotic disorders; [c] Examples are depressive and bipolar disorders

B. Switching to a vitamin K antagonist

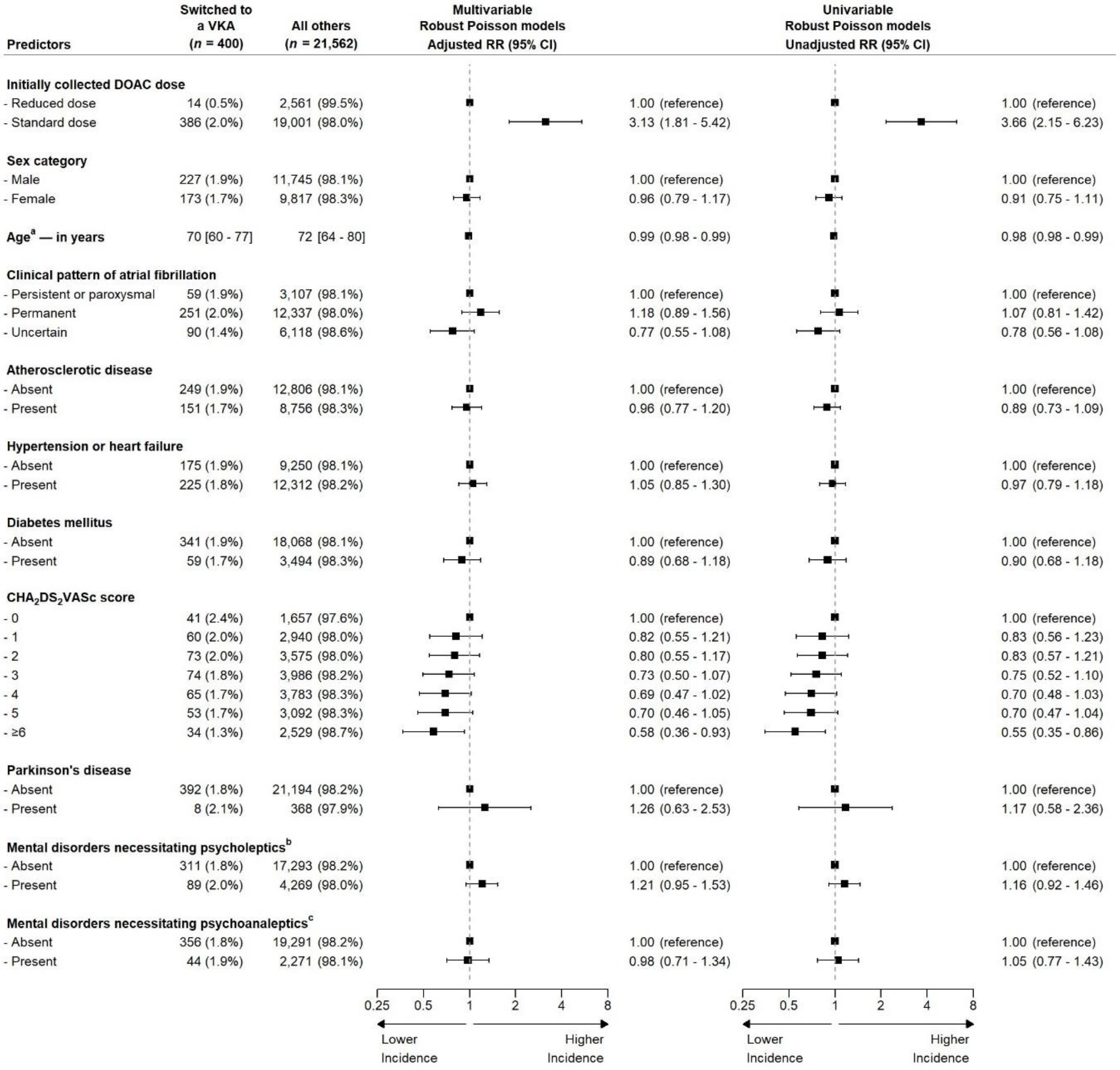

I. Apixaban

This forest plot illustrates the adjusted and unadjusted associations between baseline characteristics and switching to a VKA within the first 12 months for the patients who initially collected apixaban in the longitudinal analysis cohort (*n*=21,962). Baseline characteristics are presented in number of patients (% relative to stratum total), and the associations are in risk ratios.

CI confidence interval; *DOAC* direct oral anticoagulant; *RR* risk ratio.

[a] The relationship between age and incident switching was best fitted by a linear function in both the univariable and multivariable model; [b] Examples are sleeping, anxiety, and psychotic disorders; [c] Examples are depressive and bipolar disorders

Predictors	Switched to a VKA (*n* = 328)	All others (*n* = 15,738)	Multivariable Robust Poisson models Adjusted RR (95% CI)	Univariable Robust Poisson models Unadjusted RR (95% CI)
**Initially collected DOAC dose**				
- Reduced dose	33 (0.7%)	4,990 (99.3%)	1.00 (reference)	1.00 (reference)
- Standard dose	295 (2.7%)	10,748 (97.3%)	3.47 (2.37 - 5.09)	4.07 (2.84 - 5.82)
**Sex category**				
- Male	210 (2.3%)	9,116 (97.7%)	1.00 (reference)	1.00 (reference)
- Female	118 (1.8%)	6,622 (98.2%)	0.92 (0.73 - 1.16)	0.78 (0.62 - 0.97)
**Age[a] — in years**	68 [59 - 74]	71 [64 - 78]	0.98 (0.97 - 0.99)	0.97 (0.97 - 0.98)
**Clinical pattern of atrial fibrillation**				
- Persistent or paroxysmal	41 (1.6%)	2,454 (98.4%)	1.00 (reference)	1.00 (reference)
- Permanent	210 (2.2%)	9,368 (97.8%)	1.50 (1.07 - 2.10)	1.33 (0.96 - 1.86)
- Uncertain	77 (1.9%)	3,916 (98.1%)	1.29 (0.88 - 1.88)	1.17 (0.81 - 1.71)
**Atherosclerotic disease**				
- Absent	199 (2.0%)	9,690 (98.0%)	1.00 (reference)	1.00 (reference)
- Present	129 (2.1%)	6,048 (97.9%)	1.26 (0.99 - 1.59)	1.04 (0.83 - 1.29)
**Hypertension or heart failure**				
- Absent	148 (2.0%)	7,214 (98.0%)	1.00 (reference)	1.00 (reference)
- Present	180 (2.1%)	8,524 (97.9%)	1.16 (0.92 - 1.47)	1.03 (0.83 - 1.28)
**Diabetes mellitus**				
- Absent	280 (2.0%)	13,472 (98.0%)	1.00 (reference)	1.00 (reference)
- Present	48 (2.1%)	2,266 (97.9%)	1.00 (0.73 - 1.37)	1.02 (0.75 - 1.38)
**CHA_2DS_2VASc score**				
- 0	35 (2.5%)	1,352 (97.5%)	1.00 (reference)	1.00 (reference)
- 1	68 (2.8%)	2,318 (97.2%)	1.14 (0.76 - 1.71)	1.13 (0.76 - 1.69)
- 2	59 (2.0%)	2,947 (98.0%)	0.86 (0.57 - 1.30)	0.78 (0.51 - 1.18)
- 3	63 (2.1%)	2,979 (97.9%)	1.01 (0.67 - 1.53)	0.82 (0.55 - 1.23)
- 4	43 (1.6%)	2,687 (98.4%)	0.86 (0.55 - 1.35)	0.62 (0.40 - 0.97)
- 5	37 (1.9%)	1,961 (98.1%)	1.13 (0.71 - 1.80)	0.73 (0.46 - 1.16)
- ≥6	23 (1.5%)	1,494 (98.5%)	1.22 (0.70 - 2.12)	0.60 (0.36 - 1.01)
**Parkinson's disease**				
- Absent	323 (2.0%)	15,516 (98.0%)	1.00 (reference)	1.00 (reference)
- Present	5 (2.2%)	222 (97.8%)	1.15 (0.47 - 2.82)	1.08 (0.44 - 2.65)
**Mental disorders necessitating psycholeptics[b]**				
- Absent	274 (2.1%)	12,884 (97.9%)	1.00 (reference)	1.00 (reference)
- Present	54 (1.9%)	2,854 (98.1%)	0.95 (0.71 - 1.28)	0.89 (0.67 - 1.19)
**Mental disorders necessitating psychoanaleptics[c]**				
- Absent	294 (2.0%)	14,229 (98.0%)	1.00 (reference)	1.00 (reference)
- Present	34 (2.2%)	1,509 (97.8%)	1.09 (0.76 - 1.58)	1.09 (0.77 - 1.55)

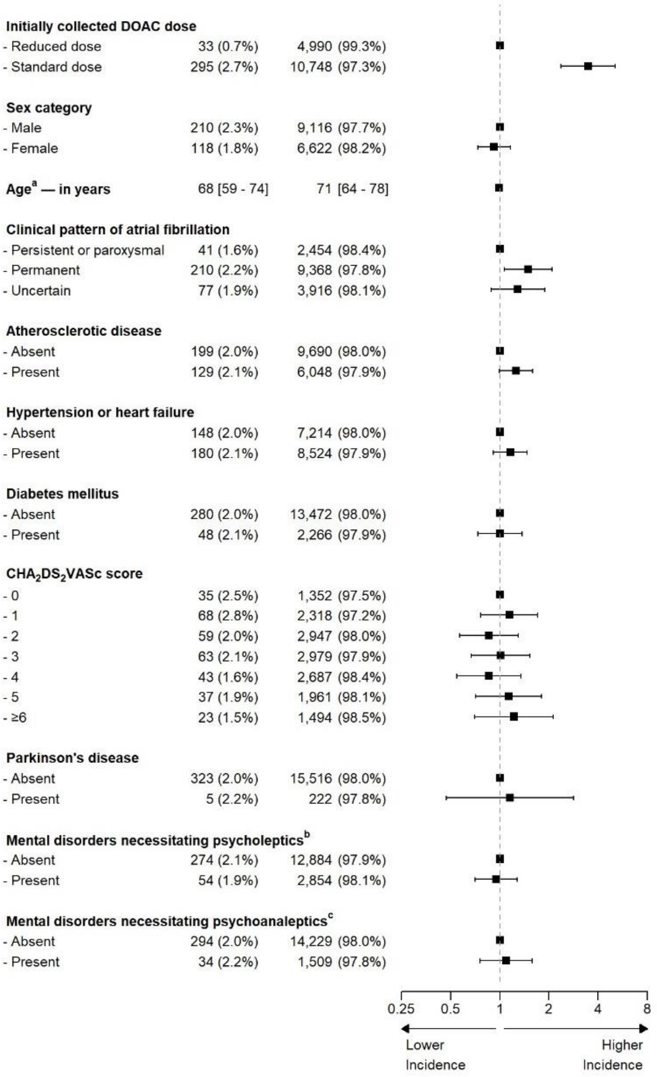

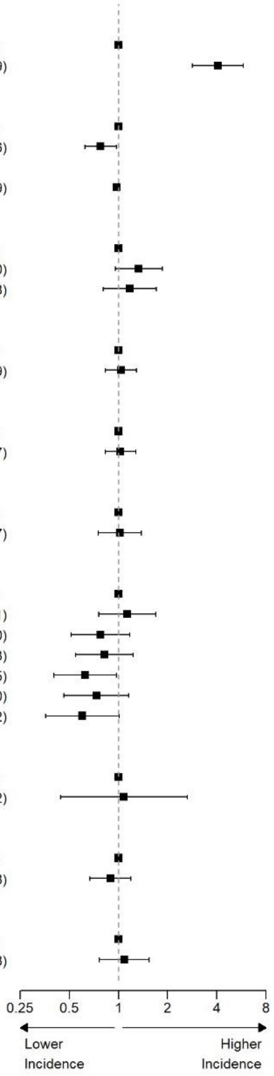

II. Dabigatran

This forest plot illustrates the adjusted and unadjusted associations between baseline characteristics and switching to a VKA within the first 12 months for the patients who initially collected dabigatran in the longitudinal analysis cohort (*n*=16,066). Baseline characteristics are presented in number of patients (% relative to stratum total), and the associations are in risk ratios.

[a] The relationship between age and incident switching was non-linear in the univariable model but linear in the multivariable model. We decided to report the results of both models using a linear fit; [b] Examples are sleeping, anxiety, and psychotic disorders; [c] Examples are depressive and bipolar disorders

Predictors	Switched to a VKA (*n* = 98)	All others (*n* = 4,930)	Multivariable Robust Poisson models Adjusted RR (95% CI)	Univariable Robust Poisson models Unadjusted RR (95% CI)
**Initially collected DOAC dose**				
- Reduced dose	27 (3.0%)	872 (97.0%)	1.00 (reference)	1.00 (reference)
- Standard dose	71 (1.7%)	4,058 (98.3%)	0.56 (0.36 - 0.88)	0.57 (0.37 - 0.89)
**Sex category**				
- Male	52 (1.8%)	2,865 (98.2%)	1.00 (reference)	1.00 (reference)
- Female	46 (2.2%)	2,065 (97.8%)	1.15 (0.78 - 1.70)	1.22 (0.83 - 1.81)
**Age[a] — in years**	69 [62 - 78]	71 [63 - 78]	0.99 (0.98 - 1.00)	1.00 (0.99 - 1.01)
**Clinical pattern of atrial fibrillation**				
- Persistent or paroxysmal	14 (2.0%)	670 (98.0%)	1.00 (reference)	1.00 (reference)
- Permanent	66 (2.3%)	2,814 (97.7%)	1.07 (0.60 - 1.93)	1.12 (0.63 - 1.98)
- Uncertain	18 (1.2%)	1,446 (98.8%)	0.61 (0.30 - 1.23)	0.60 (0.30 - 1.20)
**Atherosclerotic disease**				
- Absent	57 (1.9%)	2,990 (98.1%)	1.00 (reference)	1.00 (reference)
- Present	41 (2.1%)	1,940 (97.9%)	0.95 (0.61 - 1.47)	1.11 (0.74 - 1.65)
**Hypertension or heart failure**				
- Absent	35 (1.5%)	2,294 (98.5%)	1.00 (reference)	1.00 (reference)
- Present	63 (2.3%)	2,636 (97.7%)	1.44 (0.90 - 2.29)	1.55 (1.03 - 2.34)
**Diabetes mellitus**				
- Absent	83 (1.9%)	4,212 (98.1%)	1.00 (reference)	1.00 (reference)
- Present	15 (2.0%)	718 (98.0%)	0.90 (0.51 - 1.58)	1.06 (0.61 - 1.83)
**CHA_2DS_2VASc score**				
- 0	3 (0.7%)	435 (99.3%)	1.00 (reference)	1.00 (reference)
- 1	22 (2.7%)	786 (97.3%)	3.70 (1.11 - 12.33)	3.98 (1.20 - 13.22)
- 2	14 (1.5%)	917 (98.5%)	1.96 (0.56 - 6.82)	2.20 (0.63 - 7.61)
- 3	19 (2.1%)	894 (97.9%)	2.55 (0.75 - 8.62)	3.04 (0.90 - 10.22)
- 4	18 (2.3%)	769 (97.7%)	2.57 (0.76 - 8.77)	3.34 (0.99 - 11.28)
- 5	9 (1.4%)	657 (98.6%)	1.42 (0.38 - 5.29)	1.97 (0.54 - 7.25)
- ≥6	13 (2.7%)	472 (97.3%)	2.55 (0.73 - 8.98)	3.91 (1.12 - 13.66)
**Parkinson's disease**				
- Absent	97 (2.0%)	4,855 (98.0%)	1.00 (reference)	1.00 (reference)
- Present	1 (1.3%)	75 (98.7%)	0.67 (0.08 - 5.34)	0.67 (0.08 - 5.55)
**Mental disorders necessitating psycholeptics[b]**				
- Absent	79 (1.9%)	4,007 (98.1%)	1.00 (reference)	1.00 (reference)
- Present	19 (2.0%)	923 (98.0%)	1.05 (0.65 - 1.71)	1.04 (0.64 - 1.71)
**Mental disorders necessitating psychoanaleptics[c]**				
- Absent	91 (2.0%)	4,460 (98.0%)	1.00 (reference)	1.00 (reference)
- Present	7 (1.5%)	470 (98.5%)	0.65 (0.31 - 1.37)	0.73 (0.34 - 1.58)

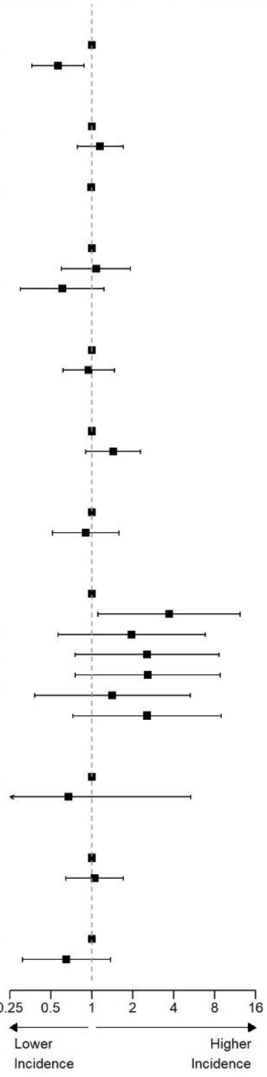

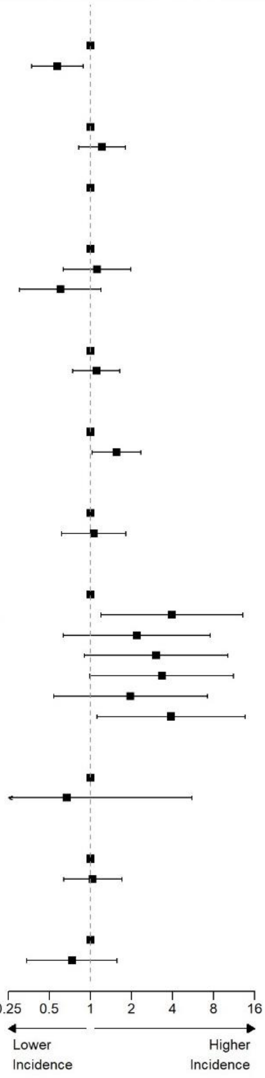

III. *Edoxaban*

This forest plot illustrates the adjusted and unadjusted associations between baseline characteristics and switching to a VKA within the first 12 months for the patients who initially collected edoxaban in the longitudinal analysis cohort (*n*=5,028). Baseline characteristics are presented in number of patients (% relative to stratum total), and the associations are in risk ratios.

[a] The relationship between age and incident switching to a different DOAC was best fitted by linear term in both the univariable and multivariable model; [b] Examples are sleeping, anxiety, and psychotic disorders; [c] Examples are depressive and bipolar disorders

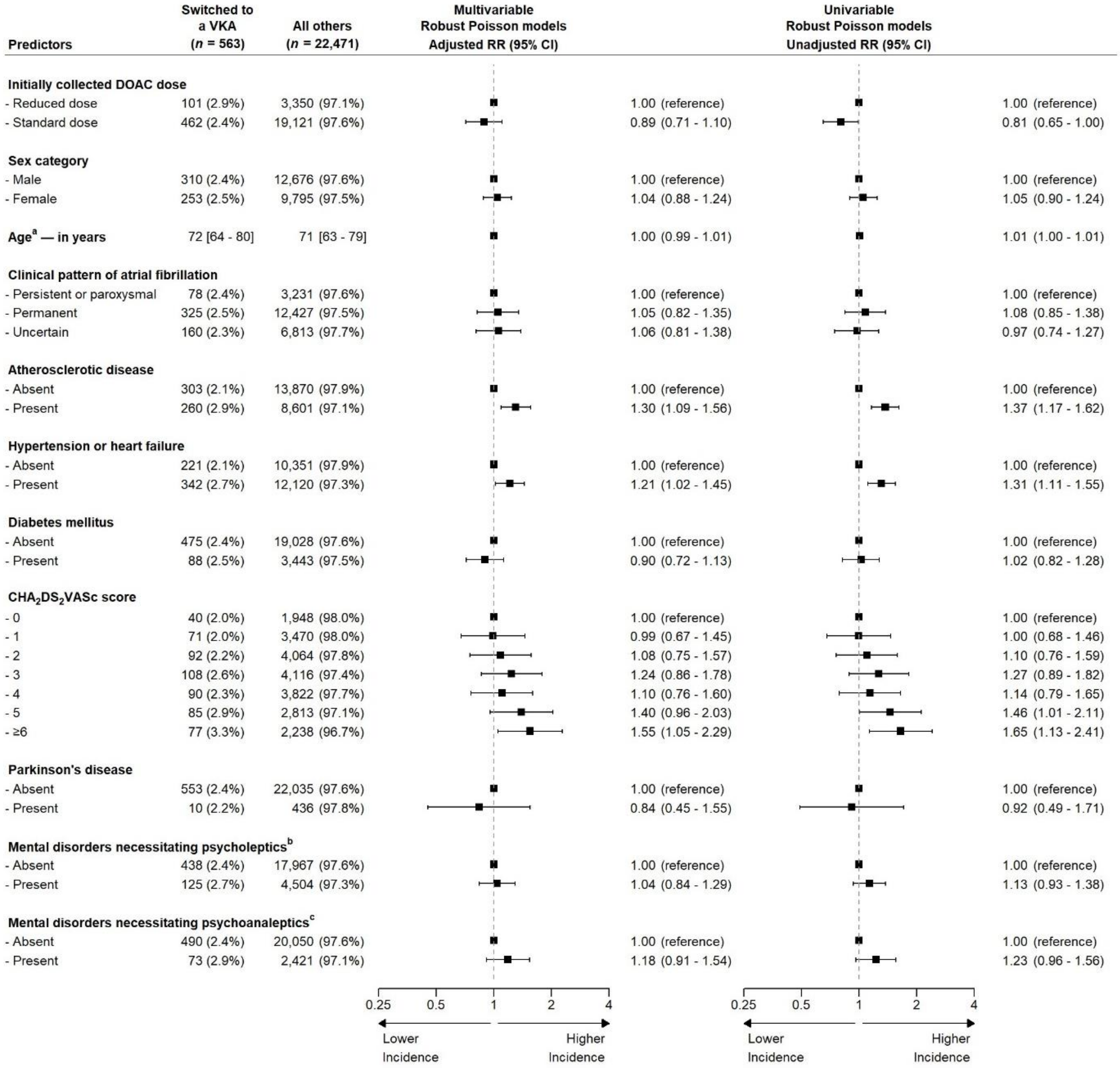

Predictors	Switched to a VKA (*n* = 563)	All others (*n* = 22,471)	Multivariable Robust Poisson models Adjusted RR (95% CI)	Univariable Robust Poisson models Unadjusted RR (95% CI)
**Initially collected DOAC dose**				
- Reduced dose	101 (2.9%)	3,350 (97.1%)	1.00 (reference)	1.00 (reference)
- Standard dose	462 (2.4%)	19,121 (97.6%)	0.89 (0.71 - 1.10)	0.81 (0.65 - 1.00)
**Sex category**				
- Male	310 (2.4%)	12,676 (97.6%)	1.00 (reference)	1.00 (reference)
- Female	253 (2.5%)	9,795 (97.5%)	1.04 (0.88 - 1.24)	1.05 (0.90 - 1.24)
**Age[a] — in years**	72 [64 - 80]	71 [63 - 79]	1.00 (0.99 - 1.01)	1.01 (1.00 - 1.01)
**Clinical pattern of atrial fibrillation**				
- Persistent or paroxysmal	78 (2.4%)	3,231 (97.6%)	1.00 (reference)	1.00 (reference)
- Permanent	325 (2.5%)	12,427 (97.5%)	1.05 (0.82 - 1.35)	1.08 (0.85 - 1.38)
- Uncertain	160 (2.3%)	6,813 (97.7%)	1.06 (0.81 - 1.38)	0.97 (0.74 - 1.27)
**Atherosclerotic disease**				
- Absent	303 (2.1%)	13,870 (97.9%)	1.00 (reference)	1.00 (reference)
- Present	260 (2.9%)	8,601 (97.1%)	1.30 (1.09 - 1.56)	1.37 (1.17 - 1.62)
**Hypertension or heart failure**				
- Absent	221 (2.1%)	10,351 (97.9%)	1.00 (reference)	1.00 (reference)
- Present	342 (2.7%)	12,120 (97.3%)	1.21 (1.02 - 1.45)	1.31 (1.11 - 1.55)
**Diabetes mellitus**				
- Absent	475 (2.4%)	19,028 (97.6%)	1.00 (reference)	1.00 (reference)
- Present	88 (2.5%)	3,443 (97.5%)	0.90 (0.72 - 1.13)	1.02 (0.82 - 1.28)
**CHA_2DS_2VASc score**				
- 0	40 (2.0%)	1,948 (98.0%)	1.00 (reference)	1.00 (reference)
- 1	71 (2.0%)	3,470 (98.0%)	0.99 (0.67 - 1.45)	1.00 (0.68 - 1.46)
- 2	92 (2.2%)	4,064 (97.8%)	1.08 (0.75 - 1.57)	1.10 (0.76 - 1.59)
- 3	108 (2.6%)	4,116 (97.4%)	1.24 (0.86 - 1.78)	1.27 (0.89 - 1.82)
- 4	90 (2.3%)	3,822 (97.7%)	1.10 (0.76 - 1.60)	1.14 (0.79 - 1.65)
- 5	85 (2.9%)	2,813 (97.1%)	1.40 (0.96 - 2.03)	1.46 (1.01 - 2.11)
- ≥6	77 (3.3%)	2,238 (96.7%)	1.55 (1.05 - 2.29)	1.65 (1.13 - 2.41)
**Parkinson's disease**				
- Absent	553 (2.4%)	22,035 (97.6%)	1.00 (reference)	1.00 (reference)
- Present	10 (2.2%)	436 (97.8%)	0.84 (0.45 - 1.55)	0.92 (0.49 - 1.71)
**Mental disorders necessitating psycholeptics[b]**				
- Absent	438 (2.4%)	17,967 (97.6%)	1.00 (reference)	1.00 (reference)
- Present	125 (2.7%)	4,504 (97.3%)	1.04 (0.84 - 1.29)	1.13 (0.93 - 1.38)
**Mental disorders necessitating psychoanaleptics[c]**				
- Absent	490 (2.4%)	20,050 (97.6%)	1.00 (reference)	1.00 (reference)
- Present	73 (2.9%)	2,421 (97.1%)	1.18 (0.91 - 1.54)	1.23 (0.96 - 1.56)

IV. Rivaroxaban

This forest plot illustrates the adjusted and unadjusted associations between baseline characteristics and switching to a VKA within the first 12 months for the patients who initially collected rivaroxaban in the longitudinal analysis cohort (*n*=23,034). Baseline characteristics are presented in number of patients (% relative to stratum total), and the associations are in risk ratios.

CI confidence interval; *DOAC* direct oral anticoagulant; *RR* risk ratio; *VKA* vitamin K antagonist.

[a] The relationship between age and incident switching was best fitted by a linear function in both the univariable and multivariable model; [b] Examples are sleeping, anxiety, and psychotic disorders; [c] Examples are depressive and bipolar disorders

References

1 von Elm E, Altman DG, Egger M, Pocock SJ, Gøtzsche PC, Vandenbroucke JP. The Strengthening the Reporting of Observational Studies in Epidemiology (STROBE) statement: guidelines for reporting observational studies. *J Clin Epidemiol*. 2008; **61**: 344-9. 10.1016/j.jclinepi.2007.11.008.

2 Steffel J, Collins R, Antz M, Cornu P, Desteghe L, Haeusler KG, Oldgren J, Reinecke H, Roldan-Schilling V, Rowell N, Sinnaeve P, Vanassche T, Potpara T, Camm AJ, Heidbüchel H, Lip GYH, Deneke T, Dagres N, Boriani G, Chao TF, Choi EK, Hills MT, Santos IS, Lane DA, Atar D, Joung B, Cole OM, Field M. 2021 European Heart Rhythm Association Practical Guide on the Use of Non-Vitamin K Antagonist Oral Anticoagulants in Patients with Atrial Fibrillation. *Europace*. 2021; **23**: 1612-76. 10.1093/europace/euab065.

3 Assessment report. INN/active substance: direct oral anticoagulants (DOACs). In: Committee for Medicinal Products for Human Use (CHMP), ed.: European Medicines Agency, 2020.

4 Connolly SJ, Ezekowitz MD, Yusuf S, Eikelboom J, Oldgren J, Parekh A, Pogue J, Reilly PA, Themeles E, Varrone J, Wang S, Alings M, Xavier D, Zhu J, Diaz R, Lewis BS, Darius H, Diener H-C, Joyner CD, Wallentin L. Dabigatran versus Warfarin in Patients with Atrial Fibrillation. 2009; **361**: 1139-51. 10.1056/NEJMoa0905561.

5 Hindricks G, Potpara T, Dagres N, Arbelo E, Bax JJ, Blomström-Lundqvist C, Boriani G, Castella M, Dan G-A, Dilaveris PE, Fauchier L, Filippatos G, Kalman JM, La Meir M, Lane DA, Lebeau J-P, Lettino M, Lip GYH, Pinto FJ, Thomas GN, Valgimigli M, Van Gelder IC, Van Putte BP, Watkins CL, Group ESCSD. 2020 ESC Guidelines for the diagnosis and management of atrial fibrillation developed in collaboration with the European Association for Cardio-Thoracic Surgery (EACTS): The Task Force for the diagnosis and management of atrial fibrillation of the European Society of Cardiology (ESC) Developed with the special contribution of the European Heart Rhythm Association (EHRA) of the ESC. *Eur Heart J*. 2021; **42**: 373-498. 10.1093/eurheartj/ehaa612.

6 Chan NC, Eikelboom JW, Weitz JI. Evolving Treatments for Arterial and Venous Thrombosis: Role of the Direct Oral Anticoagulants. *Circ Res*. 2016; **118**: 1409-24. 10.1161/CIRCRESAHA.116.306925.

7 Knuuti J, Wijns W, Saraste A, Capodanno D, Barbato E, Funck-Brentano C, Prescott E, Storey RF, Deaton C, Cuisset T, Agewall S, Dickstein K, Edvardsen T, Escaned J, Gersh BJ, Svitil P, Gilard M, Hasdai D, Hatala R, Mahfoud F, Masip J, Muneretto C, Valgimigli M, Achenbach S, Bax JJ, Group ESCSD. 2019 ESC Guidelines for the diagnosis and management of chronic coronary syndromes: The Task Force for the diagnosis and management of chronic coronary syndromes of the European Society of Cardiology (ESC). *European Heart Journal*. 2020; **41**: 407-77. 10.1093/eurheartj/ehz425.

8 Falck-Ytter Y, Francis CW, Johanson NA, Curley C, Dahl OE, Schulman S, Ortel TL, Pauker SG, Colwell CW, Jr. Prevention of VTE in orthopedic surgery patients: Antithrombotic Therapy and Prevention of Thrombosis, 9th ed: American College of Chest Physicians Evidence-Based Clinical Practice Guidelines. *Chest*. 2012; **141**: e278S-e325S. 10.1378/chest.11-2404.

9 Konstantinides SV, Meyer G, Becattini C, Bueno H, Geersing G-J, Harjola V-P, Huisman MV, Humbert M, Jennings CS, Jiménez D, Kucher N, Lang IM, Lankeit M, Lorusso R, Mazzolai L, Meneveau N, Ní Áinle F, Prandoni P, Pruszczyk P, Righini M, Torbicki A, Van Belle E, Zamorano JL. 2019 ESC Guidelines for the diagnosis and management of acute pulmonary embolism developed in collaboration with the European Respiratory Society (ERS): The Task Force for the diagnosis and management of acute pulmonary embolism of the European Society of. *European Heart Journal*. 2020; **41**: 543-603. 10.1093/eurheartj/ehz405.

10 Over het FK. [Internet] *Farmacotherapeutisch Kompas*. Cited on. https://www.farmacotherapeutischkompas.nl/algemeen/over-het-fk.

11 Geneesmiddelenoverzicht directwerkende orale anticoagulantia voor alle indicaties. [Internet] *Farmacotherapeutisch Compas*. Cited on.

https://www.farmacotherapeutischkompas.nl/bladeren/groepsteksten/directwerkende_orale_anticoagulantia.

12 Apixaban. [Internet] *Farmacotherapeutisch Kompas*. Cited on: September 10, 2022. https://www.farmacotherapeutischkompas.nl/bladeren/preparaatteksten/a/apixaban.

13 Dabigatran. [Internet] *Farmacotherapeutisch Kompas*. Cited on: September 10, 2022. https://www.farmacotherapeutischkompas.nl/bladeren/preparaatteksten/d/dabigatran.

14 Edoxaban. [Internet] *Farmacotherapeutisch Kompas*. Cited on: September 10, 2022. https://www.farmacotherapeutischkompas.nl/bladeren/preparaatteksten/e/edoxaban.

15 Rivaroxaban. [Internet] *Farmacotherapeutisch Kompas*. Cited on: September 10, 2022. https://www.farmacotherapeutischkompas.nl/bladeren/preparaatteksten/r/rivaroxaban.

16 Lawrence DB, Cai TT, Anirban D. Interval Estimation for a Binomial Proportion. *Statistical Science*. 2001; **16**: 101-33. 10.1214/ss/1009213286.

17 Zou G. A modified poisson regression approach to prospective studies with binary data. *Am J Epidemiol*. 2004; **159**: 702-6. 10.1093/aje/kwh090.

18 Mansournia MA, Nazemipour M, Naimi AI, Collins GS, Campbell MJ. Reflection on modern methods: demystifying robust standard errors for epidemiologists. *International Journal of Epidemiology*. 2021; **50**: 346-51. 10.1093/ije/dyaa260.

19 Cribari-Neto F, Souza TC, Vasconcellos KLP. Inference Under Heteroskedasticity and Leveraged Data. *Communications in Statistics - Theory and Methods*. 2007; **36**: 1877-88. 10.1080/03610920601126589.

20 Gauthier J, Wu QV, Gooley TA. Cubic splines to model relationships between continuous variables and outcomes: a guide for clinicians. *Bone Marrow Transplant*. 2020; **55**: 675-80. 10.1038/s41409-019-0679-x.

21 Harrell FE. General Aspects of Fitting Regression Models. In: Harrell JFE, ed. *Regression Modeling Strategies: With Applications to Linear Models, Logistic and Ordinal Regression, and Survival Analysis*. Cham: Springer International Publishing, 2015, 13-44.

22 Maydeu-Olivares A, García-Forero C. Goodness-of-Fit Testing. In: Peterson P, Baker E, McGaw B, eds. *International Encyclopedia of Education (Third Edition)*. Oxford: Elsevier, 2010, 190-6.

23 Baker CL, Dhamane AD, Mardekian J, Dina O, Russ C, Rosenblatt L, Lingohr-Smith M, Menges B, Lin J, Nadkarni A. Comparison of Drug Switching and Discontinuation Rates in Patients with Nonvalvular Atrial Fibrillation Treated with Direct Oral Anticoagulants in the United States. *Advances in Therapy*. 2019; **36**: 162-74. 10.1007/s12325-018-0840-8.

24 Baker CL, Dhamane AD, Rajpura J, Mardekian J, Dina O, Russ C, Rosenblatt L, Lingohr-Smith M, Lin J. Switching to Another Oral Anticoagulant and Drug Discontinuation Among Elderly Patients With Nonvalvular Atrial Fibrillation Treated With Different Direct Oral Anticoagulants. *Clinical and Applied Thrombosis/Hemostasis*. 2019; **25**: 1076029619870249. 10.1177/1076029619870249.

25 Pham PN, Brown JD. Real-world adherence for direct oral anticoagulants in a newly diagnosed atrial fibrillation cohort: does the dosing interval matter? *BMC Cardiovascular Disorders*. 2019; **19**: 64. 10.1186/s12872-019-1033-3.

26 Gopalakrishnan C, Schneeweiss S, Bartels DB, Zint K, Santiago Ortiz A, Huybrechts KF. Evaluating utilization patterns of oral anticoagulants in routine care. *Journal of Thrombosis and Haemostasis*. 2019; **17**: 1033-43. 10.1111/jth.14467.

27 Manzoor BS, Walton SM, Sharp LK, Galanter WL, Lee TA, Nutescu EA. High number of newly initiated direct oral anticoagulant users switch to alternate anticoagulant therapy. *Journal of thrombosis and thrombolysis*. 2017; **44**: 435-41. 10.1007/s11239-017-1565-2.

28 Chowdhury MZI, Turin TC. Variable selection strategies and its importance in clinical prediction modelling. *Fam Med Community Health*. 2020; **8**: e000262. 10.1136/fmch-2019-000262.

29 Kim JH. Multicollinearity and misleading statistical results. *Korean J Anesthesiol*. 2019; **72**: 558-69. 10.4097/kja.19087.

30 R Core Team (2022). R: A language and environment for statistical computing. *R Foundation for Statistical Computing, Vienna, Austria.* https://www.R-project.org/
